# Homozygous deletions implicate non-coding epigenetic marks in Autism spectrum disorder

**DOI:** 10.1038/s41598-020-70656-0

**Published:** 2020-08-20

**Authors:** Klaus Schmitz-Abe, Guzman Sanchez-Schmitz, Ryan N. Doan, R. Sean Hill, Maria H. Chahrour, Bhaven K. Mehta, Sarah Servattalab, Bulent Ataman, Anh-Thu N. Lam, Eric M. Morrow, Michael E. Greenberg, Timothy W. Yu, Christopher A. Walsh, Kyriacos Markianos

**Affiliations:** 1 Division of Genetics and Genomics, Department of Pediatrics, Boston Children’s Hospital, Boston, MA 02115 USA; 2 Divisions of Newborn Medicine and Manton Center for Orphan Disease Research, Department of Pediatrics, Boston Children’s Hospital, Boston, MA 02115 USA; 3 Department of Pediatrics, Harvard Medical School, Boston, MA 02115 USA; 4 Broad Institute of Harvard and MIT, Cambridge, MA 02115 USA; 5 Division of Infectious Diseases, Department of Pediatrics and Precision Vaccines Program, Boston Children’s Hospital, Boston, MA 02115 USA; 6 Department of Neurobiology, Harvard Medical School, Boston, MA 02115 USA; 7 Department of Molecular Biology, Cell Biology and Biochemistry and Department of Psychiatry and Human Behavior, Brown University, Providence, RI 02912 USA; 8 Howard Hughes Medical Institute, Boston Children’s Hospital, Boston, MA 02115 USA; 9 Department of Neurology, Harvard Medical School, Boston, MA 02115 USA; 10 Center for Data and Computational Sciences, Cooperative Studies Program, VA Boston Healthcare system, Boston, MA 02130 USA

**Keywords:** Gene regulation, Computational neuroscience

## Abstract

More than 98% of the human genome is made up of non-coding DNA, but techniques to ascertain its contribution to human disease have lagged far behind our understanding of protein coding variations. Autism spectrum disorder (ASD) has been mostly associated with coding variations via de novo single nucleotide variants (SNVs), recessive/homozygous SNVs, or de novo copy number variants (CNVs); however, most ASD cases continue to lack a genetic diagnosis. We analyzed 187 consanguineous ASD families for biallelic CNVs. Recessive deletions were significantly enriched in affected individuals relative to their unaffected siblings (17% versus 4%, *p* < 0.001). Only a small subset of biallelic deletions were predicted to result in coding exon disruption. In contrast, biallelic deletions in individuals with ASD were enriched for overlap with regulatory regions, with 23/28 CNVs disrupting histone peaks in ENCODE (*p* < 0.009). Overlap with regulatory regions was further demonstrated by comparisons to the 127-epigenome dataset released by the Roadmap Epigenomics project, with enrichment for enhancers found in primary brain tissue and neuronal progenitor cells. Our results suggest a novel noncoding mechanism of ASD, describe a powerful method to identify important noncoding regions in the human genome, and emphasize the potential significance of gene activation and regulation in cognitive and social function.

## Introduction

ASDs are a family of neurodevelopmental conditions characterized by atypical social interactions, communication, and repetitive and stereotyped interests. ASDs represent a spectrum of conditions of varying severity, and may or may not be accompanied by intellectual disability, epilepsy, or other features; underscoring this phenotypic heterogeneity, mutations in genes associated with classical monogenic neurological disorders can also cause autism^1–3^. The genetic contribution of ASD is well documented from twin studies^4–8^ and established contributions have been determined for: (A) de novo mutations, including CNVs and SNVs^9–18^; (B) inherited heterozygous CNVs^12^, (C) inherited recessive mutations^3,19–22^ and (D) somatic mutations^23,24^. Genetic studies have implicated synaptic proteins as well as chromatin remodeling factors^17,20^. In contrast, roles for noncoding mutations have been difficult to ascertain. A study of post-mortem brain samples from ASD cases and matched controls indicates differences in acetylation patterns (H3K27ac) in prefrontal and temporal cortex^25^ while recent whole gene sequencing in large family samples indicates contributions from de novo point mutations^26^ and inherited structural variants^27^ in promoter regions.

**Figure 1:**
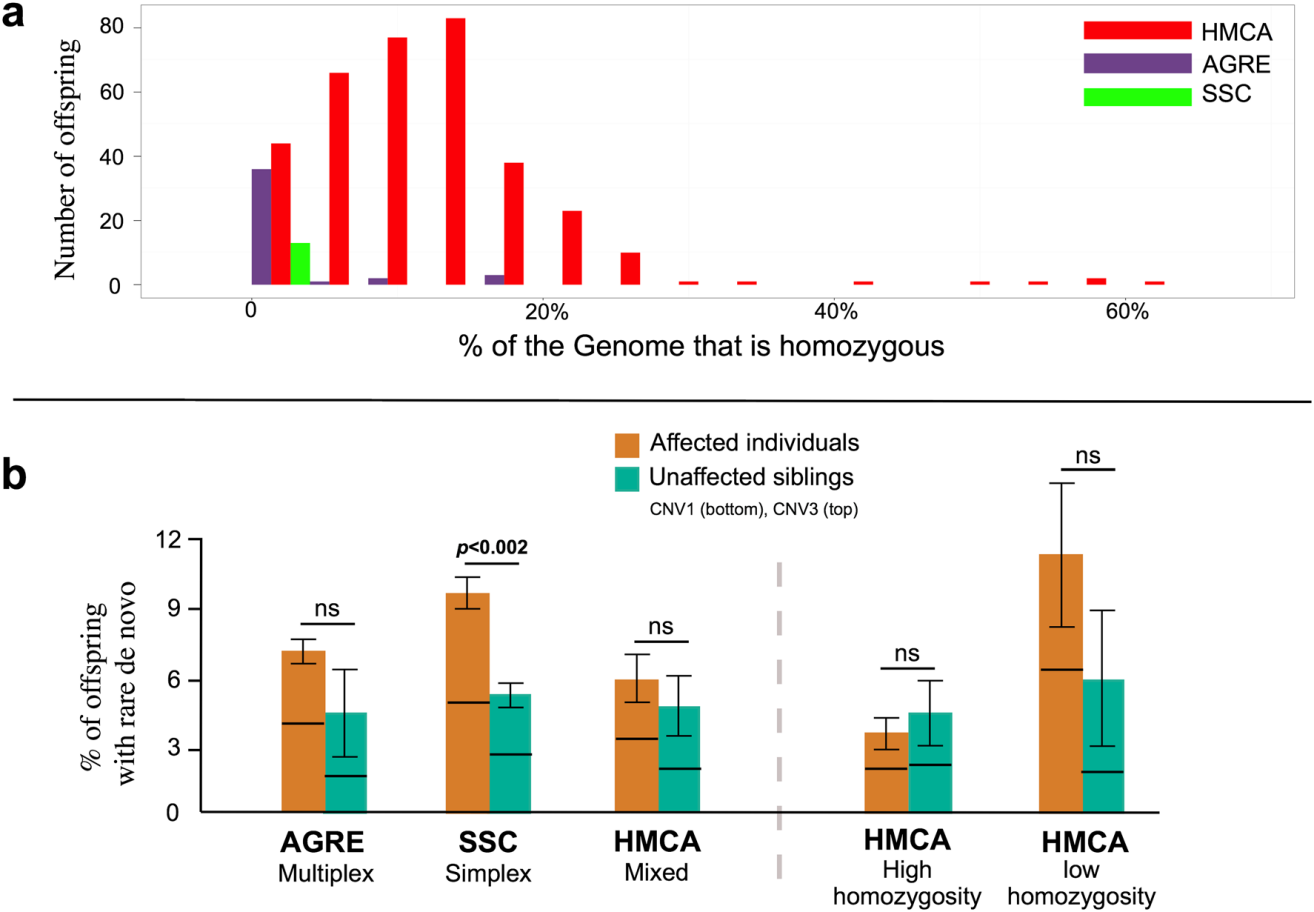
Homozygosity and de novo CNV rates in three different ASD collections. (**a**) Observed homozygosity in the HMCA, AGRE, and SSC: Distribution of recent homozygosity (homozygous intervals 5 cM or longer in autosomes) within individuals from each cohort. For display purposes, samples with no homozygosity are not shown. (**b**) Burden of rare de novo CNV events in three ASD collections. De novo copy number events are observed more frequently in affected individuals across all three cohorts, although within individual cohorts, it reaches statistical significance only in the SSC (Fisher test, one sided). Within the HMCA, high homozygosity families do not show an excess of de novo copy number mutation. Families with low homozygosity show a trend towards excess, but this does not reach significance likely due to sample size. Results are presented in a stacked bar plot (CNV1 bottom, CNV3 top, Probes ≥ 25). Numbers of samples and ratios for each comparison are shown in Table S2.

We analyzed CNVs and homozygosity from 187 families ascertained through the Homozygosity Mapping Collaborative for Autism, HMCA, an ASD cohort highly enriched for families that are consanguineous (Fig. 1a) and/or have multiple affected children (255 affected, 790 individuals genotyped with SNP chips, Table 1). We compared CNV patterns with 740 families from the Autism Genetic Resource Exchange cohort (AGRE, 2,985 individuals) and 1,027 families from the Simons Simplex Collection (SSC, 3,881 individuals). We set out to take advantage of the high degree of shared ancestry within families of the HMCA to analyze patterns and rates of homozygous CNV. Our analysis shows that homozygous deletions are significantly enriched in cases compared to controls in these families and surprisingly tend to implicate DNA regulatory sequences rather than coding exons. Since the functional impact of complete deletion of both alleles is so much more directly evident than single copy deletion, these data provide some of the strongest evidence to date that noncoding mutations are important in ASD risk.

**Table 1:** Summary of data sets used in this study: Homozygosity Mapping Collaborative of Autism (HMCA), Autism Genetic Resource Exchange (AGRE), Simons Simplex Collection (SSC) and HapMap (control samples).

	HMCA collection	AGRE	Simons Simplex	HapMap	Total
# of families	187	740	1,027	801	2,755
# of samples	790	2,985	3,881	1,251	8,907
affected individuals (offspring) [Unaffected siblings]	255 [169]	1,463 [94]	1,027 [798]	0 [856]	2,745 [1,917]
affected parents	13	4	0		
% of families with both parents	84%	85%	100%	20.10%	
% of consanguineous families	66%	0.40%	0%	1.50%	
% of multiplex families	22%	87%	0%	0%	
male/female ratio (affected) [Unaffected siblings]	3.63 [0.76]	3.69 [0.71]	6.55 [0.83]	n/a [1.03]	
SNP array technology	Affy 6.0 & 500 K	Affy 5.0	Illumina 1 M	Affy 6.0 & 500 K	

For each dataset, the table presents the fraction of families with: both parents, consanguinity, and 2 or more affected children (multiplex families). In the bottom of the table we show the male/female ratio for both affected individuals (offspring) and unaffected siblings. Additional information can be found in Methods (Description of datasets).

## Results

We developed a CNV detection, annotation, and analysis platform (see Methods, “Variant Explorer pipeline”, VExP) for application to the HMCA and AGRE cohorts, employing concordant calls between multiple algorithms to maximize specificity. For the SSC, existing published CNV calls^10^ were used. We identified, on average, 6.1 copy number losses and 3.4 gains per individual. Of these, 0.8 copy losses and 0.5 gains per individual were rare, defined as entirely absent from 1,251 HapMap controls processed by the same pipeline (Methods: D,E). Common and rare CNV burdens were not significantly different between collections, supporting the comparability of these datasets (algorithms ≥ 3, Tables S1a-b).

Although the HMCA was collected on the basis of self-reported consanguinity, observed levels of homozygosity vary substantially between families (Fig. 1a). To illustrate the impact of overall homozygosity we often subdivide the HMCA sample into high and low homozygosity families throughout the manuscript. Families were classified as highly homozygous if at least one child was homozygous for 2.5% or more of the autosomal genome (see Methods: F,G).

### De novo CNVs contribute to risk in families with limited shared ancestry

Prior to analysis of biallelic deletions we examined the presence of single copy variants in our families, a well-established cause of Autism, and compared results with the other two family collections. In the nonconsanguineous cohorts of the SSC and AGRE, results from our analytic pipeline replicated the expected enrichment of rare de novo CNV in cases versus controls^9,10^. The SSC^10^ demonstrated an excess of rare de novo CNVs, (9.5% versus 5.7%, *p* < 0.002; Figs. 1b & S2, Table S2, Probes >  = 25), while the AGRE cohort^15^, comprised primarily of nonconsanguineous families with multiple affected individuals, showed a modest difference between cases and controls (7.1% versus 5%). In the HMCA the de novo CNV rate was similar among affected and unaffected individuals (5.9 vs 5.3%, p = NS). A few de novo events were observed in HMCA cases that are likely to contribute to risk in some patients, including three 16p11.2 micro-deletions^16^. For this analysis we counted all families, including families with potentially pathogenic recessive point mutations identified through WES^3^ (Table S1c). None of the 11 families with candidate exonic mutations diagnosed through WES harbored rare de novo CNVs. Excluding the 11 families with potentially explanatory mutations would slightly elevate the reported de novo CNV rate. The sharp differences in de novo CNV rates in the SSC versus the AGRE and HMCA emphasizes the differential contribution of de novo CNV to ASD risk in distinct family structures.

### Biallelic deletions contribute to ASD in consanguineous families

**Figure 2:**
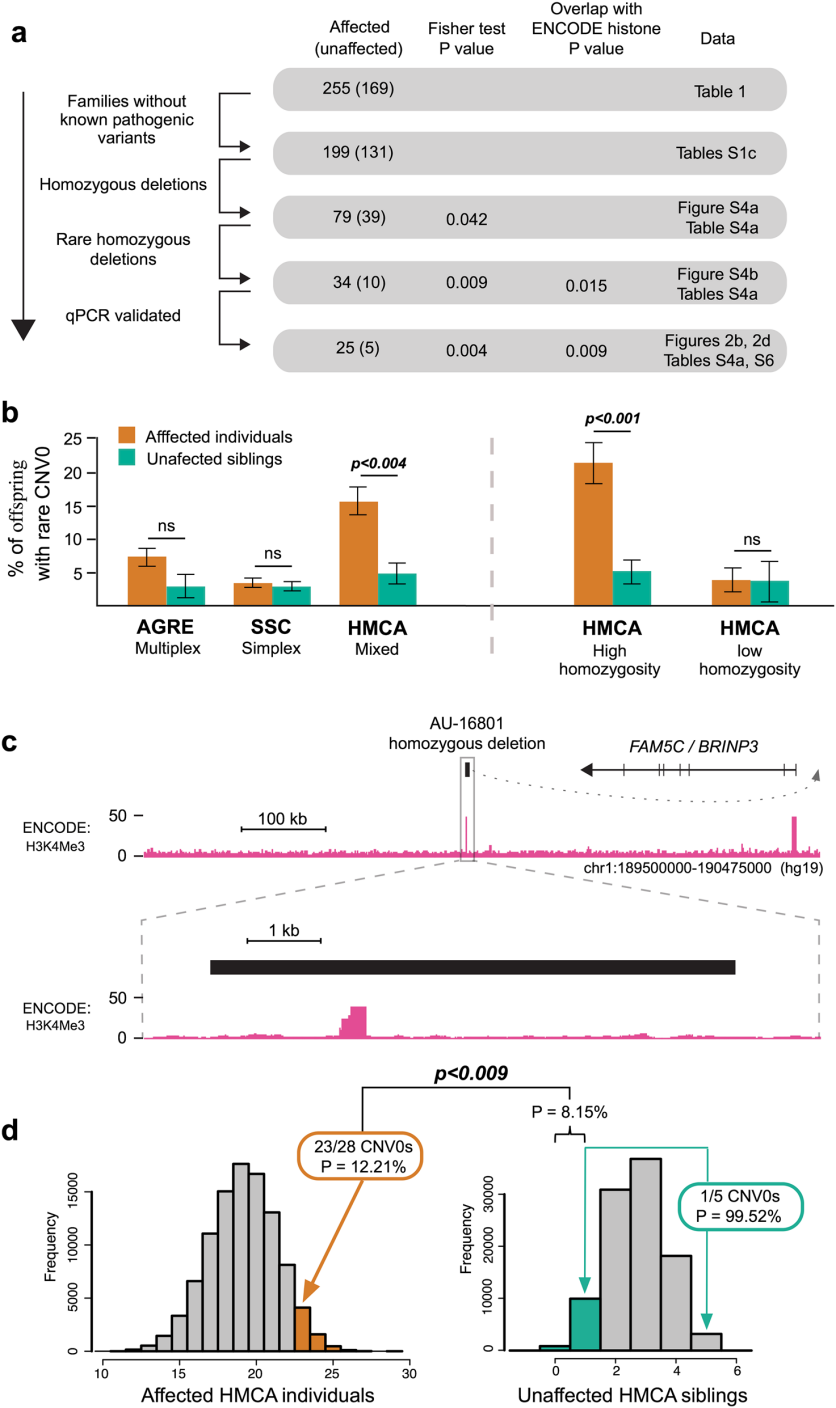
Homozygous deletions and relation to histone modification marks. (**a**) Selection of homozygous deletions (CNV0). We used a series of increasingly stringent selection criteria to compare CNV0 rate in affected individuals versus unaffected siblings and evaluate the overlap between biallelic deletions and ENCODE histone peaks. (**b**) Burden of rare homozygous deletions (CNV0) in three ASD collections (Table 1). Percentage of affected individuals and unaffected siblings with one or more rare biallelic events. Affected individuals show an elevated rate of biallelic deletions in all datasets. The difference is significant only in the HMCA collection (Fisher test, one sided) and is driven by consanguineous families (high homozygosity). The corresponding number of samples and ratios are shown in Table S4a. (**c**) Example of a non-coding biallelic deletion (AU-16801, Table 2). This particular homozygous deletion is approximately 7 kb in size, and it removes an H3K4Me3 histone modification mark in the vicinity of *BRINP3* / *FAM5C* gene. The ENCODE profile shown represents the cell lines profiles available from UCSC. **(d)**  Empirical distribution of the number of coincidences in HCMA families between biallelic deletions and 3 histone modification marks (H3K4Me1, H3K4Me3, and H3K27Ac) as defined by the ENCODE project. We randomize location of qPCR confirmed biallelic deletions. For events denoted in the HMCA families (Table 2), the joint probability to observe such an enrichment/depletion pattern is *p* < 0.009. ENCODE regions are defined using a score ≥ 20, and conclusions are robust regarding the threshold (Table S6). Simulations excluded sex chromosomes and low marker coverage regions (Methods: J,K).

HMCA families showed enrichment for rare homozygous deletions (CNV0) in cases versus controls. We examined the relative abundance of biallelic deletions in the HMCA using a series of increasingly stringent selection criteria (Fig. 2a). First, in an effort to reduce genetic heterogeneity, we excluded from this analysis 14 families that have previously identified mutations that are likely to be causative, including large, rare, de novo or inherited (heterozygous) CNVs and 11 families harboring previously described recessive exonic point mutations^3^ (Table S1c). The remaining 162 families (330 samples) were screened for biallelic deletions (CNV0) requiring concordance of at least 3 of 4 CNV algorithms. To filter out common CNVs, we then excluded any loci observed as CNV0 in 1,251 HapMap controls. Finally, we validated candidate deletions using qPCR, recovering a total of 33 experimentally validated “CNV0” events (66%, Table S3a, Methods: N). At every selection step, affected individuals showed higher rates of biallelic deletions than unaffected siblings, and the difference became more significant despite a progressive reduction in the number of events surviving selection (Fig. 2a). 28 CNV0s (25 patients) were found in 199 affected individuals (12.5%) and only 5 CNVs (5 samples) occurred in 131 unaffected siblings (3.8%). We refined and validated a subset of the CNV boundaries using standard PCR (Table S3b).

The excess of validated biallelic deletions among affected individuals in the HMCA was driven by families showing high levels of homozygosity within the HMCA (defined as > 2.5% of the diploid genome, see Methods: F). Overall, 17.2% of affected individuals showed CNV0 versus 4.1% of unaffected individuals in high homozygosity families (*p* < 0.001, Figs. 2b & S4, Table S4a). The excess of CNV0 in cases easily persisted after adjusting for excess homozygosity (*p* < 0.009, Table S4b). In families from the HMCA with lower overall homozygosity, there was no difference in CNV0 rates between cases and controls (3% versus 2.9%). In AGRE and SSC, nonconsanguineous cohorts, homozygous deletions were extremely rare, and did not significantly differ in prevalence, between cases and controls (Fig. 2b). The > fourfold excess of CNV0 in cases versus controls from consanguineous families of the HMCA is consistent with the expectation that removal of both alleles of the genome is highly damaging and likely to contribute to disease risk. Comparison of CNV0 rates among cases and controls (Table S4b) suggests that the majority of these CNV0 are causative and contribute to ASD risk in up to 8% of patients in consanguineous families.

### Homozygous deletions interrupting gene-coding regions

The most straightforward mechanism for homozygous deletions to confer disease risk would be to delete entire genes or exons. Of the 28 confirmed CNV0s from affected individuals 10 events disrupted 7 genes: *PABPC4L*, *C3orf58*, *AGR3*, *CD36*, *MSR1*, *BCAS1* and *MTMR3* (Table 2). Based on gnomAD (https://gnomad.broadinstitute.org), *CD36* tolerates a large number of Loss of Function (LoF) mutations, including homozygous LoF variants, therefore it is likely to be non-essential. *C3orf58*, also known as *DIA1*^21^, regulates phosphorylation of extracellular phosphoproteins^28^, but the large *C3orf58*-associated deletion also affects noncoding DNA sequences near *NHE9* (*SLC9A9,* Figure S5c), a gene regulated by neuronal depolarization^21^, and so these noncoding segments might also be essential. Deletions in *PABPC4L* have been associated with major depressive disease in a single study^29^. Inherited deletions disrupting *MSR1* were previously reported in two Autism trios^30^. The CNV interrupting *MSR1* is the only double deletion of the 28 discovered here that is listed in DECIPHER^31^, but is listed there as “Likely benign”, so its contribution to risk in this family is unproven. *BCAS1* has been identified as an ASD candidate gene based on its enrichment in an autism-associated protein interaction module and this finding was further validated by exome sequencing of an independent cohort of 505 ASD cases and 491 controls^32^. Two other genes (*AGR3*, *MTMR3*) have not been previously associated with ASD or neurodevelopmental disorders.

**Table 2:** List of all qPCR confirmed rare biallelic deletions (CNV0) among individuals with ASD in the HMCA collection.

# of CNV	chr	Start	Size (Kb)	Histone peak				Gene location			
				ENCODE project	Primary neuron	Road map neuron	Road map brain	Exonic	Intronic	On the left	On the right
1	1	189,959,475	6.9	Y							***FAM5C***
1	1	191,473,007	179.0	Y	Y	Y	Y			***FAM5C***	*RGS18, RGS21*
1	2	167,346,017	47.1	Y	Y	Y	Y			***SCN7A***	
1	2	184,794,451	8.0			Y				*DUSP19*, ***NCKAP1***, *NUP35*	***ZNF804A***
1	2	227,341,510	6.1	Y	Y	Y	Y			*NYAP2*	***IRS1***, *RHBDD1*
1	2	242,915,454	119.2	Y		Y	Y			*CXXC11,* ***D2HGDH***, *DTYMK*, *GAL3ST2*, *ING5*, ***NEU4***, *PDCD1*	
1	3	1,782,524	5.1							***CNTN6***	***CNTN4***
1	3	75,394,265	149.8	Y	Y	Y	Y			***CNTN3***	***ZNF717***
1	3	143,637,504	853.2	**Y**	Y	Y	Y	***C3orf58***		***SLC9A9***	
1	4	134,871,302	321.4	Y	Y	Y	Y	***PABPC4L***			
1	5	9,904,421	20.6	Y		**Y**				***TAS2R1***	***CCT5***, *CMBL, FAM173B, MARCH6*
1	6	154,121,271	10.1		Y		Y			***FBXO5***, *MTRF1L*, ***RGS17***	*IPCEF1,* ***OPRM1***
3	7	16,900,135	15.3	Y				*AGR3*		*AGR2*, ***BZW2***, ***TSPAN13***	
1	7	80,157,064	141.8	Y	Y	Y	Y	***CD36***		*GNAT3*	***SEMA3C***
1	7	159,049,219	13.1		No data		Y			***VIPR2***, ***WDR60***	
1	8	15,937,585	88.5	Y		Y		***MSR1***			
1	8	18,852,675	9.6	Y	Y	Y	Y		*PSD3*		
1	8	34,800,058	43.0	**Y**		Y	Y				***UNC5D***
1	10	81,512,254	85.7	Y	Y	Y	Y			*AK302451, EIF5AL1, SFTPA1, SFTPA2, ZCCHC24*	*PLAC9, SFTPD*, ***TMEM254***
1	12	112,432,874	5.6	Y		Y	Y		*TMEM116*	***ALDH2****, ****MAPKAPK5***	***ERP29,*** *HECTD4, NAA25, TRAFD1*
1	14	28,475,766	25.0	Y	Y	Y	Y				***FOXG1***
1	14	47,966,854	2.6						***MDGA2***		
2	20	52,643,162	20.2	Y	Y	Y	Y	*BCAS1*			***CYP24A1***, *PFDN4*
1	21	18,802,512	19.7	Y	Y	**Y**	Y				*BTG3*, ***CXADR***
1	22	30,336,496	30.3	Y	Y	Y	Y	*MTMR3*		*ASCC2, CABP7*, ***NF2***, , ***UQCR10***, ZMAT5	*HORMAD2*

The table notes overlap with histone peaks as defined by the ENCODE Project^33^, by ChIP-seq data from Primary–Neuron^34^, and both Brain and Neuron epigenomes from Roadmap Project (ChromHMM state model^36^. Neighboring genes are shown, and genes with bibliographic evidence linking them to neurodevelopmental disorders are noted in bold. Table S5a list rare homozygous deletions (CNV0) for unaffected siblings.

### Homozygous deletions implicate non-coding epigenetic marks in ASD

The remaining homozygous deletions (18/28) interrupted only noncoding DNA. This observation suggested that some of them may contribute to disease risk by disrupting regulatory elements important for temporal or spatial expression of nearby genes. To test this hypothesis, we cross-referenced 3 histone modification marks (H3K4Me1, H3K4Me3, and H3K27Ac) as defined by the ENCODE project^33^, that correspond to states of gene transcription and enhancer activity (Methods: K). Together, these peaks cover only 6.96% of the mappable genome. Nonetheless, in affected individuals, 23 of 28 validated events overlapped these peaks, versus 1 of 5 events in unaffected siblings (Tables 2 or S5a).

We assessed the statistical significance of the overlap of CNV0 with epigenetic marks using Monte Carlo simulations. We ran 100,000 simulations randomly placing deletions with a size distribution identical to the observed events on the autosomal genome. For this analysis we count as overlap the intersection of a CNV with epigenetic marks from any one of the epigenome profiles in the 9 cell lines available from ENCODE project. Centromeres and low marker density regions were excluded from this analysis (Methods: J). The simulation suggested that the coincidence of CNV0 with epigenetic marks was higher than expected among cases (23 observed, 16 expected), and suppressed among unaffected siblings (1 observed, 3 expected). The joint probability to observe a more extreme enrichment/depletion pattern is highly significant (*p* < 0.009, Fig. 2d). The significance of this result was robust to variation of the threshold used to define histone peaks (Table S6). We applied the same analysis to the few homozygous deletions observed in the SSC and AGRE families, but we did not observe such an enrichment/depletion pattern (Table S6). In addition to evidence from the ENCODE project, the importance of histone marks is supported by ChIP-seq experiments interrogating the same three modifications in human Primary Neurons^34^ as can be seen in Fig. 4a–d (Table 2). However, genome-wide coverage for this data set is not uniform and so it was not used for estimation of p-values (Methods: L).

**Figure 3:**
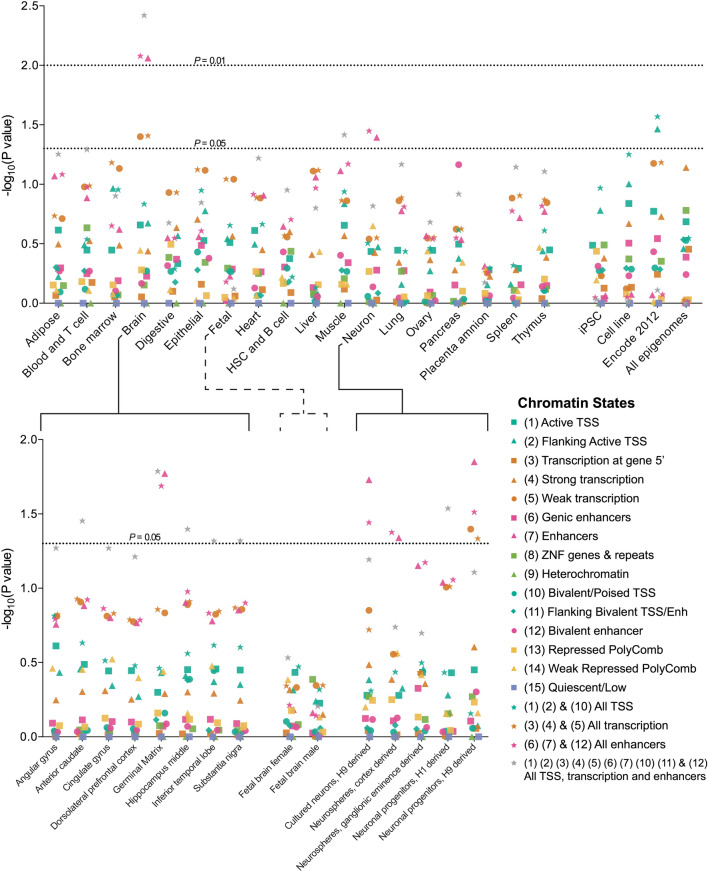
Overlap of homozygous deletions with regulatory regions defined by the Epigenome Roadmap Project. Illustrated are p-values for coincidence between non-coding homozygous deletions and epigenetic marks. Most significant correlations are observed among primary Brain cells and Neuronal profiles. We use 127 profiles provided by the Epigenome Roadmap Project (Table S7a) and the 15-state ChromHMM model to test enrichment/depletion of coincidences in affected/unaffected individuals (noncoding CNVs defined by KnownGene annotation). Similar results can be found in Supplemental Information using alternative gene annotations (RefGene and Ensembl, Figures S6a-b, Tables S7b-e).

We expanded our Monte Carlo analysis from the 9 ENCODE lines to 127 lines available from the Roadmap Epigenomics Project. This release offers expanded cell type diversity, most importantly primary brain cells and cultured neuronal cell lines and provides analysis tools that allow uniform processing and quality control across a large number of epigenomes (Table S7a). As before, we ran 100,000 simulations randomizing the position of the observed biallelic deletions (Methods: M). For this analysis the presence of epigenetic marks is defined by a widely used model, ChromHMM defined by the Epigenetics Roadmap Project^35^, with 5 chromatin marks (H3K4me3, H3K4me1, H3K36me3, H3K27me3, H3K9me3). This release includes 111 epigenomes^36^ plus 16 epigenomes from the earlier phase of the project (ENCODE12). Although we do not have one on one cell type correspondence (Table S7a), comparison between the previous ENCODE analysis (*p* < 0.009, Fig. 2d) and the ENCODE12 profiles confirms the previous result and identifies significant correlation (*p* < 0.02, Fig. 3).

When we compared different cell types, brain tissues and iPS derived neuronal cells showed the highest correlation between epigenetic marks and homozygous deletions (Fig. 3 and Table S7c). For clarity of presentation when multiple profiles are available for the same tissue, we present a union of all epigenomes from the same tissue (Table S7a). Primary brain tissues showed the most significant correlation (*p* < 0.004), predominantly for germinal matrix cells. We also looked at the relative contribution of different epigenetic states to our results. ChromHMM analysis highlighted the importance of enhancers: 17/28 CNV0s from affected individuals overlapped brain cell enhancers, while there was no enhancer overlap for CNV0s from unaffected samples (*p* < 0.009, Tables S7b-c). Significant correlations were also observed for enhancers in cultured neuron profiles (*p* < 0.019) especially in neuronal progenitor cells (*p* < 0.015). These observations provide strong evidence that disruption of gene regulatory elements contributes to risk in HMCA individuals, and suggestive evidence that at least some of the increased risk arises from disruption of neural enhancers.

### Genes near deletions

**Figure 4:**
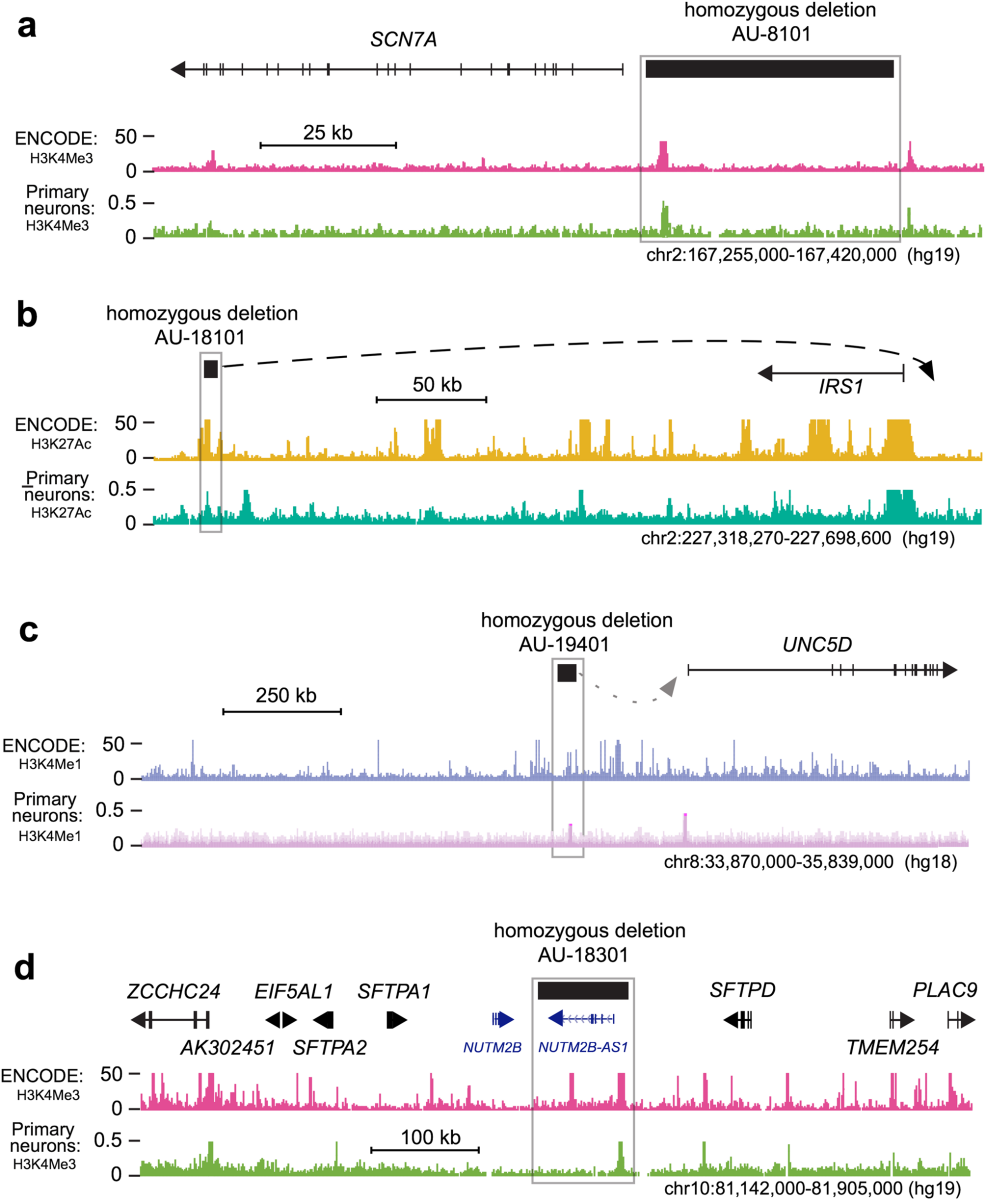
Examples of overlap between non-coding biallelic deletions and histone peaks as defined by the ENCODE project. In addition, we show histone peaks derived by ChIP-seq data from Primary Neuron culture. ENCODE or Primary Neuron profiles shown in the figures represent the union of all cell lines available. Additional examples are presented in Figures S5a-c. (**a**) Non-coding biallelic deletion for sample AU-8101. The homozygous deletion removes the *SCN7A* promoter as defined by RNA-Seq data. (**b**) Non-coding biallelic deletion for sample AU-18101. Published chromatin interaction data obtained from human fibroblasts demonstrate that one broadly active element directly interacts with the *IRS1* gene promoter. (**c**) Non-coding biallelic deletion for sample AU-19401 upstream of *UNC5D,* a gene encoding a receptor implicated in neuronal axon guidance and cell survival. (**d**) Non-coding biallelic deletion for sample AU-18301. The homozygous deletion interrupts a non-coding gene (*NUTM2B-AS1*), a broadly expressed antisense transcript on the opposite strand of *NUTM2B*.

Following our hypothesis that biallelic deletions might be disrupting regulatory elements, we examined the genes that neighbored validated homozygous deletions (Table 2). 70 genes were found within 500 kb of homozygous deletions, 11 within 500–900 kb, and one within 1 Mb (median 185 kb, mean 262 kb, see Methods: H,I, Table S8a). We gathered functional annotations from PubMed, UCSC genome-browser and human-brain-map. We checked if particular genes were previously associated with ASD or neurodevelopmental disorders. Among the identified transcripts is *FAM5C* (also known as *BRINP3*, encoding a BMP-retinoic acid-inducible neural specific protein 3). It is implicated in cell cycle control of mouse neural stem cells^37,38^ and located near two non-overlapping 7 and 179 kb biallelic deletions found in two unrelated families (AU-16800, AU-18000, Figs. 2c & S5a). *UNC5D*, a receptor implicated in neuronal axon guidance and cell survival^39^, lies downstream of a segregating 43 kb CNV0 (AU-19400, Fig. 4c). *UNC5D* was a gene of interest reported in a prior homozygosity study in the SSC^40^ and a single deletion in the same region was also reported in an independent ASD collection^41^.

Close examination of the 13 CNVs overlapping only histone marks and not disrupting protein-coding exons (Table 2), shows evidence of impact on promoter function or pseudogenes and long noncoding RNA transcripts (lncRNAs). Such functional elements are known to be associated with epigenetic marks. One CNV0 is upstream of *SCN7A* (Fig. 4a, previously reported^21^), encoding a sodium channel that controls oxytocin and vasopressin release^42^, and based on signatures of neural histone modifications and RNA-Seq data^36^, removes its promoter. Potential roles of lncRNAs in ASD have been difficult to define in general, though the moesin pseudogene 1 antisense transcript is an example that has been proposed to contribute to the effects of the common SNP, rs4307059 on chromosome 5p14.1, in a region linked to ASD^43^. In our analysis, three CNVs directly impact expressed lncRNAs (*ENSG00000234172*, *ENSG00000233806*, *ENSG00000232560*), and two overlap highly expressed processed pseudogenes (*SERTAD4-AS1*, *NUTM2B-AS1*). In contrast, unaffected siblings do not impact expressed lncRNAs. Antisense and lncRNA transcripts may regulate the expression of many genes independent of proximity on the chromosome. Of particular interest is *ENSG00000233806* (AU-19503, Figure S5b), which is highly expressed in neural tissues with very little expression in non-neuronal tissues, suggesting a neural-specific regulatory role^36^. Another CNV interrupts *NUTM2B-AS1* (AU-18301, Fig. 4d), a broadly expressed antisense transcript on the opposite strand of *NUTM2B*, a coding gene of unknown function.

Beyond CNVs affecting transcriptionally active loci, we also identified 8 CNV0s affecting predicted regulatory elements, including several active in neural tissues from the Epigenomics Roadmap datasets^36,44^. While the targets of such elements are difficult to predict, existing chromatin interaction data from human fibroblasts demonstrates that one broadly active element directly interacts with the *IRS1* gene promoter^36,44^ (Fig. 4b), thereby likely regulating its transcriptional activity. *IRS1* encodes a signaling protein that is part of the insulin-like growth factor (IGF) signaling pathway in many tissues^17^. Together, the loss of regulatory elements, lncRNAs and promoters might help explain the potential roles of CNVs which do not remove protein coding regions of the genome.

### Relationship to TADs

An alternative regulatory mechanism that can be disrupted by double deletions is the 3D organization of the genome^45^. We examined boundary disruption of Topologically Associated Domains (TADs). Cell type specific TADs have not been assessed in most brain regions and/or developmental time points. Cognizant of these limitations, we collected curated TAD profiles (94 total, 3D Genome Browser^46^). We looked for cases where the entire TAD boundary region is removed by a double deletion, an event most likely to disrupt 3D genome organization. We find that 3/28 events among affecteds and 0/5 events among unaffecteds disrupt TAD boundaries (Table S9). However, we are not confident that the 3 events have functional consequences as two of the 3 CNV0s also disrupt coding sequence with plausible functional consequences (*C3orf58*, *PABPC4L*). Using a permissive definition of TAD boundary disruption, removal of just the start or stop site of a TAD but not necessarily the entire boundary, we find 12/28 double deletions disrupt TADs among affecteds while the rate is 0/5 among unaffecteds (Table S9). The result is intriguing because we observe potential TAD disruption only among affecteds and intersection in multiple tissues, including one hippocampus profile. However, due to limitations outlined above and the small number of observations, it is difficult to draw robust conclusions.

### High connectivity between ASD associated genes in independent data sets

**Figure 5:**
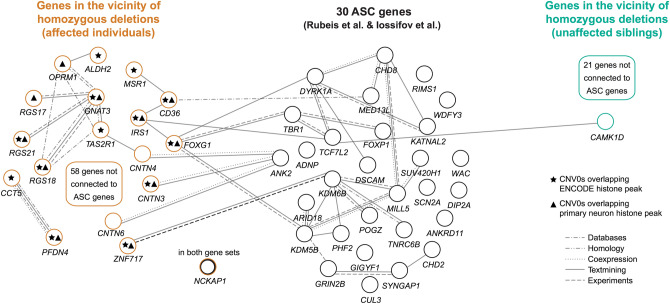
Protein–Protein Interactions between genes in proximity to homozygous deletions and 30 ASC genes^16,20^. STRING identifies interactions between 21/30 ASC genes and 16/76 genes (11 affected individuals) in the neighborhood of qPCR validated biallelic deletions from Table 2 (*p* < 6e-5, see Figure S7c). In contrast, STRING predicts only one interaction between ASC genes and the 22 genes from 5 unaffected siblings (*p* = NS, Figure S7d). For display clarity, disconnected genes from individuals are excluded from the figure.

We finally examined the gene set defined by proximity to homozygous deletions for enrichment in protein–protein interactions using STRING (https://string-db.org). Genes neighboring homozygous deletions in affected subjects demonstrated significantly more protein–protein inter-connectivity (*p* < 0.03, Figures S7a-b, permutation p-values is derived directly by STRING) than neighboring genes identified from unaffected siblings (*p* = NS). Because this observation does not necessarily imply a mechanistic relationship to autism, we cross-analyzed protein–protein interactions between our gene list and 30 high-confidence ASD genes identified through whole exome sequencing^16,20^. The gene list from affected individuals demonstrated significantly more connectivity with the 30 ASD genes (*p* < 6*10^–5^) than those from unaffected siblings (*p* = NS, Figs. 5 and S7c-d). One gene, *NCKAP1*, is present in both gene sets. The sharp contrast in connectivity suggests concordance for two very different approaches to ASD gene discovery: biallelic deletions and de novo SNVs from WES. Furthermore, nearly all of the homozygous deletions that were near connected genes disrupted ENCODE and Primary Neuron histone modification peaks (Fig. 5).

## Discussion

While the importance of regulatory elements has been previously anticipated^17^, identifying disease-impactful non-coding mutations is more difficult than identifying coding mutations. Studying pedigrees with high rates of consanguinity allows us to study biallelic deletions and take advantage of the favorable signal to noise ratio provided by complete loss of coding or regulatory regions. The importance of biallelic deletions is sustained by two lines of evidence: 1) a significantly higher rate of homozygous deletions in affected individuals relative to their unaffected siblings (*p* < 0.004 in all HMCA, *p* < 0.001 among highly consanguineous families, Fig. 2b & Table S4a), and 2) a striking enrichment/depletion pattern of intersection between homozygous deletions and ENCODE control regions in affected/unaffected individuals (*p* < 0.009, Fig. 2d and Table S6). The excess burden of biallelic deletions establishes unambiguous evidence for their contribution to recessive ASD, while ENCODE analysis provides insight into their genetic mode of action, with significant overlap between homozygous deletions and chromatin peaks annotated from primary brain (*p* < 0.004, Fig. 3, Table S7c) and enhancers in neuronal progenitor cells (*p* < 0.015). While our statistical analysis has focused mainly on contributions of enhancer deletions to risk, based on their uniform markings and unambiguous definition, some of the CNV0 described here may contribute to risk by other mechanisms, such as deletions of noncoding RNAs, or disruption of topological associated domains, which have recently been described to have important consequences on gene expression in the setting of rare structural variants^45^.

Comparisons of rates and types of CNV between consanguineous and nonconsanguineous families show complementary patterns of CNV, depending upon family structure. Biallelic deletions were most consistently enriched in high consanguinity families (Fig. 2b and Table S4a) whereas de novo CNVs were most enriched in cases in the outbred families of the SSC (Fig. 1b and Table S2). There is no reason to suspect that this difference reflects suppression of de novo CNV events in consanguineous families. Instead, since neurological disorders are increased overall in consanguineous families, attributable to increased recessive disease^47^, sporadic events are more likely merely to be less common as a fraction of the whole.

Noncoding deletions, identified through analyses like those presented here, may provide an important foothold to begin to understand the role of patterned gene activation/regulation in cognitive and social function. ASD as a diagnosis appears to be especially sensitive to gene dosage, given well-established contributions of de novo or inherited heterozygous CNV and SNVs (which typically act via haploinsufficiency), hypomorphic recessive mutations, and studies implicating neuronal activity-regulated genes^3,21,22,48^. Biallelic noncoding mutations may provide mechanistic insights into the cis-regulatory mechanisms by which dosage alterations lead to ASD.

## Methods

### Description of datasets

This study integrated three different ASD data sets and the International HapMap project control samples (Table 1). All samples have passed quality control (Methods: A,B,C).

- 1) HMCA: 187 consanguineous ASD families from the Middle East (255 affected offspring, 169 unaffected siblings, 790 samples, 22% of families have more than one affected child and 66% families are consanguineous) using Affymetrix 6.0 and 500 K SNP microarrays (71 and 184 affected samples respectively and 37 and 132 unaffected samples) performed at the Broad Institute and Dana Farber Cancer Institute respectively. This cohort was recruited by the HMCA (Homozygosity Mapping Collaborative of Autism), an international multicenter effort to identify consanguineous families enriched for recessive causes of ASD. Individuals were included in this study following provision of written informed consent according to protocols approved by the institutional review boards of Boston Children’s Hospital, Beth Israel Deaconess Medical Center, and local institutions. Most families were recruited in the Middle East and Turkey. Inclusion criteria included a diagnosis of Autism or ASD by a neurologist, child psychiatrist, or psychologist and families with consanguinity and/or multiple affected individuals. Diagnostic and Statistical Manual of Psychiatric Disease IV-Revised (DSMIV-R) criteria were confirmed in all individuals with an Autism diagnosis by a team of Boston Children’s Hospital-affiliated clinicians (clinical psychology, genetics, developmental medicine, and neurology). This dataset is available in the National Database for Autism Research (https://www.ncbi.nlm.nih.gov/gap).
- 2) Simons Simplex Collection: 1,027 ASD families, each comprised of a single unaffected parents, and, in most kindreds, an unaffected sibling (1,027 affected offspring, 798 unaffected siblings, 3,881 samples). This cohort is non-consanguineous and was genotyped using Illumina 1Mv1 and 1Mv3 SNP microarrays. All samples had confirmed ASD diagnoses, including Autism (89.5%), pervasive development mental disorder (8.5%) and Asperger syndrome (2%). Additional information can be found in www.sfari.org.
- 3) AGRE Collection: 740 ASD families were genotyped in Affymetrix 5.0 microarrays (1,463 affected offspring, 94 unaffected siblings, 2,985 samples). For this collection recruitment focused on inherited causes of ASD and the majority of the families include 2 or more affected children (87%, Table 1). One disadvantage of this dataset is that it only has 94 unaffected offspring versus 1,463 affected, making difficult comparisons of cases versus controls. More information can be found in www.autismspeaks.org.
- 4) HapMap control data: 1,301 samples hybridized with Affymetrix 6.0 (version Phase III, including the original 270 samples used in Phase I and II). We also included 270 samples using Affymetrix 500 K microarrays (Phase I). After quality control, 1,251 unique samples were used in our study (Table 1). Cohorts are composed of trios and singletons and they come from 11 populations around the world (https://www.sanger.ac.uk/resources/downloads/human/hapmap3.html).

### Bioinformatic analyses

A custom pipeline (“Variant Explorer Pipeline”, VExP) was employed to automate quality control, relationship checking, linkage, homozygosity and CNV calling, as well as joint analyses of all 8,907 samples (Table 1). Poorly hybridized samples and families with pedigree relationships inconsistent with observed genotypes were removed for this study. Automation of the process minimizes the error inherent in manual curation of large data sets and greatly speeds up interpretation of the results. Details are described below and VExP is available upon request and will be available in the data repository reference.

- (A) Chip quality control: Samples with chip quality values exceeding default thresholds in both Genotyping Console (QC < 0.4, MAPD > 0.4) and BirdSuite (for Affymetrix 6.0 SNP microarrays call rate < 97 and for Affymetrix 5.0 and 500 K call rate < 95) were removed from the analysis. Furthermore, outliers in terms of the number of CNVs or overall CNV coverage of the genome were eliminated (for Affymetrix 6.0 SNP microarrays we use # of CNVs < 200 and for Affymetrix 5.0 and 500 K # of CNVs < 125). Thresholds were calibrated using publicly available HapMap data genotyped with the same SNP array technology.
- (B) Gender: VExP counts the number of heterozygous/homozygous SNPs in the X and Y chromosome to determine gender. Samples with gender assignment errors were eliminated as potential labeling errors.
- (C) Pedigree structure: Pedigree errors can affect de novo CNV, homozygosity and linkage analysis. The pipeline performed a rigorous relationship test for each family in all datasets; individuals with inconsistent genotypes were reassigned when appropriate, otherwise removed from further consideration. VExP counts the number of shared genotypes for siblings and alleles for parent–child pairs in a test similar to the procedure employed by PLINK.
- (D) Classification of rare CNVs: To distinguish between common, thus likely innocuous, and rare CNVs, we compiled a variant catalog using 1,251 samples from the International HapMap project. An event is classified as previously observed if there is more than 50% overlap with an event appearing one or more times in the HapMap controls. The pipeline automatically classifies new findings against known variants matching not only locus but also copy number call (0, 1, 3 or 4). If a CNV appears as CNV1 but never as CNV0 in HapMap, the biallelic deletion will qualify as rare. This approach differs from typical CNV classification where variation is annotated simply as copy gain or copy loss; however, it is crucial for the identification of recessive biallelic deletions.
- (E) Copy number calling: For CNV analysis, four calling algorithms were used to increase specificity: BirdSuite (Version 1.5.5), PennCNV (Version Feb27-2011), Nexus (Version 7.5) and Affymetrix Genotyping Console (Version 4.1).
- (F) Classification of families based on observed homozygosity: The pipeline uses the available genotypes to compute actual, as opposed to self-reported, homozygosity and classify families into high homozygosity and low homozygosity categories. Throughout this manuscript, families were classified as highly homozygous if at least one child was homozygous for 2.5% or more of the autosomal genome. The threshold, using genetic rather than physical distances, is permissive enough to include 95% of the progeny from first cousin marriages (Figure S1). Furthermore, only homozygosity runs 5 cM or longer were considered to enrich for recent ancestry and avoid the effect of residual population homozygosity that is likely innocuous and tolerated by the natural selection. The 5 cM threshold is small enough to admit ~ 95% of homozygous segments present in 2^nd^ cousin marriages (Figure S1). It rejects just 50% of the segments inherited from a founder that lived 16 generations ago.
- (G) Homozygosity regions: The pipeline uses a sliding window approach, 100 SNPs, and retained segments with a minimum of 98% homozygosity. It retains segments where observed homozygosity exceeds 5 cM. We use genetic, as opposed to physical distance, for all calculations. To calculate overall homozygosity, we sum all segments exceeding 5 cM.
- (H) Gene model: The Variant Explorer pipeline relies on 3 gene annotations for definition of exon and UTR boundaries: UCSC KnownGene, RefGene and Ensembl (Table S10). We use KnownGene as our default gene annotation (Tables 2, S5a-b and S8a-b).
- (I) Genes neighboring biallelic deletions (Genomic distance approach): Tables 2 and S5a-b present genes in the neighborhood of biallelic deletions. These are the genes most likely to be affected by biallelic deletions. Selection is based on a simple, widely used criterion, genomic distance. The gene lists were compiled using the following three criteria: a) Only coding genes are considered. b) The list includes only the nearest gene plus any genes within a 150 kb window of the nearest coding region. Genes in close proximity are likely to be affected by the same chromosomal conformation changes. c) Genes cannot be more than 1 Mb + 150 kb window away from the CNV. We acknowledge that the structural folding of the DNA could account for unforeseen interactions between the deleted regions and other genes located outside of our cutoff distance. The ~ 1 Mb window we used is consistent with results from chromosome conformation capture experiments^13,49^. We note that the definition of neighboring genes will affect only the STRING connectivity analysis.
- (J) Monte Carlo Simulation: We sought to formally evaluate statistical significance for coincidence between biallelic deletions and epigenetic marks. We generated 100,000 data sets, each with a set of biallelic deletions identical in number and length to the observed data set but with randomly placed start positions. To compensate for inherent in-homogeneities in genotyping array coverage, we eliminated from our simulation genomic regions of low probe density (defined as intervals in which the distance between adjacent markers was > 100 kb), yielding a mappable autosomal genome of 2.6 Gb (Figures S6c-d and Table S11). For every simulation we counted the number of CNV intervals intersecting chromatin peaks. The "# of coincidences" distribution, 100,000 outcomes, is shown as a histogram in Fig. 2d. The basic procedure, position randomization and counting of intersections, was the same for the initial ENCODE (9 cell lines) and subsequent Epigenome Roadmap Project analysis (ERP, 127 cell lines). There were some important differences in the two simulations. For ENCODE analysis we used individual profiles for each one of the epigenetic marks (H3K4Me1, H3K4Me3 & H3K27Ac) encoded as enrichment intensity values, one per 25 base pairs of sequence (chromosome 1 to 22). We called peak intervals using a fixed threshold (≥ 20, Fig. 2d and Table S6) and we performed the Monte Carlo analysis multiple times using different thresholds to evaluate sensitivity of results to threshold choice. As can be seen in Table S6, the conclusions are robust for thresholds ranging from 20 to 40. We count as a coincidence the intersection of a CNV interval with a peak interval from any one of the three marks. For the Roadmap data set, 127 lines, we use profiles ChromHMM states^35^ as defined by Epigenome Roadmap Project^36^. The model defines the presence/absence of epigenetic marks as a binary outcome after a probabilistic evaluation: comparing ChIP-seq and whole-cell extract control sequencing. The analysis is performed in 200 bp bins and only the highest probability state is reported for each location. We used coding gene definitions from UCSC (KnownGene database), RefGene and Ensembl (Tables S7c-e) and exclude CNVs interrupting coding sequence.
- (K) ENCODE project^33^: The Encyclopedia of DNA Elements (ENCODE) Consortium is an international collaboration of research groups funded by the National Human Genome Research Institute (NHGRI). The goal of ENCODE is to build a comprehensive parts list of functional elements in the human genome, including elements that act at the protein and RNA levels, and regulatory elements that control cells and circumstances in which a gene is active. We examined ChIP-seq data gathered from 9 cell lines (GM12878, H1-hESC, HepG2, HMEC, HSMM, HUVEC, K562, NHEK and NHLF) and 3 histone modifications (H3K4Me1, H3K4Me3, and H3K27Ac). Epigenetic profiles were downloaded from ENCODE-UCSC (genome.ucsc.edu/ENCODE/downloads.html).
- (L) Primary neuron data^34^ were never used for formal computation of p-values due to the technical difficulties introduced by primary culture experiments: limited input DNA and heterogeneity in the cell culture produces uneven coverage and noise in the genome profile. Furthermore, there were not enough biological replicates to make comprehensive statistical comparisons. Thus, we used primary neuron profiles only as secondary support. Epigenetic profiles can be downloaded from the NCBI data repository (https://www.ncbi.nlm.nih.gov/geo/query/acc.cgi?acc=GSE78688).
- (M) ChromHMM: We evaluated co-location of epigenetic marks and homozygous deletions using the 15-state ChromHMM model v1.10 defined by the Epigenetics Roadmap Project. It is defined over 127 epigenomes with complete coverage for 5 marks (H3K4me3, H3K4me1, H3K36me3, H3K27me3, H3K9me3, Table S7a). The model provides a profile of genomic locations important for genome regulation. Furthermore, it captures combinatorial interactions between different chromatin marks in their spatial context (chromatin states). The model defines the presence/absence of epigenetic marks as a binary outcome after a probabilistic evaluation: comparing ChIP-seq and whole-cell extract control sequencing. The analysis is performed in 200 bp bins and only the highest probability state is reported for each location. This is in contrast to the initial ENCODE analysis (9 lines) where enrichment scores were used directly to define the location of epigenetic marks. We note that we did not perform a formal multiple testing correction since many of the tissues overlap and are highly correlated, making the exact number of “tests” not simple to determine; thus, these results should be considered only preliminary and important to confirm in larger sample sizes as they become available. They nonetheless provide guidance as to relevant tissues types.
- (N) CNV validation and family segregation using quantitative PCR (qPCR) and droplet-digital PCR (ddPCR): Predicted homozygous deletions were subject to experimental validation by qPCR. To characterize validated biallelic deletions more carefully, we analyzed their familial segregation patterns using ddPCR (Figure S3). The boundaries of 10 deletions were refined using standard qPCR (Table S3b). PCR probes (for qPCR analysis) and/or TaqMan assays (for droplet-digital PCR analysis) were designed to target the predicted deletion interval and flanking DNA as controls. SYBR Green qPCR reactions were conducted using conventional methods. Droplet-digital TaqMan assays were assembled using 2X ddPCR Mastermix (Bio-Rad), 20X TaqMan primers and probe assay mix (final concentrations of 900 nM each primer and 250 nM probe), and 1 μl template (10 ng/μl concentration) in a total volume of 20 μl. Reaction mixtures were then loaded into an eight-channel disposable droplet generator cartridge (Bio-Rad) together with 60 μl of droplet generation oil (Bio-Rad), placed in the droplet generator (Bio-Rad), and then transferred to a 96-well PCR plate. Thermal cycling was performed: 95 °C 10 min, 94 °C 30 s and 60 °C 60 s (40 cycles), 98 °C 10 min, and 4 °C hold. Samples were then processed on a droplet reader (Bio-Rad) and analyzed with QuantaSoft analysis software (Bio-Rad). 33 of 50 predicted events (28 of 40 affected, 5 of 10 unaffected, Table S3a) validated using these methods. As shown in Figure S3, validation was high when a CNV was called by ≥ 3 algorithms. For all events called by 2 or more algorithms, the replication rate was 26/39 (66%) for CNV1 and 23/26 (88%) for biallelic deletions. With 3 or more algorithms validation rate increases to 90% and 95% respectively. As expected, biallelic deletions had a higher replication rate than single copy loss events. The criteria stated here, ≥ 3 algorithms and at least five probes supporting discovery were used to define biallelic deletions throughout the manuscript.

## Supplementary information

Supplementary file1
