# Supplementary material for: Homozygous deletions implicate non-coding epigenetic marks in Autism spectrum disorder
Source: Sci Rep. 2020 Aug 20;10:14045. doi: 10.1038/s41598-020-70656-0 (PMC7441318; doi:10.1038/s41598-020-70656-0)
Supplement: Supplementary file 1 — Supplementary file1 [file 41598_2020_70656_MOESM1_ESM.pdf]

“Homozygous deletions implicate non-coding epigenetic marks in Autism spectrum disorder “

Klaus Schmitz-Abe, Guzman Sanchez-Schmitz, Ryan N. Doan, R. Sean Hill, Maria Chahrour, Bhaven K. Mehta, Sarah Servattalab, Bulent Ataman, Anh-Thu N. Lam, Eric M. Morrow, Michael E. Greenberg, Timothy W. Yu, Christopher A. Walsh and Kyriacos Markianos

Klaus.Schmitz-Abe@childrens.harvard.edu

Supplemental Information (Figures)

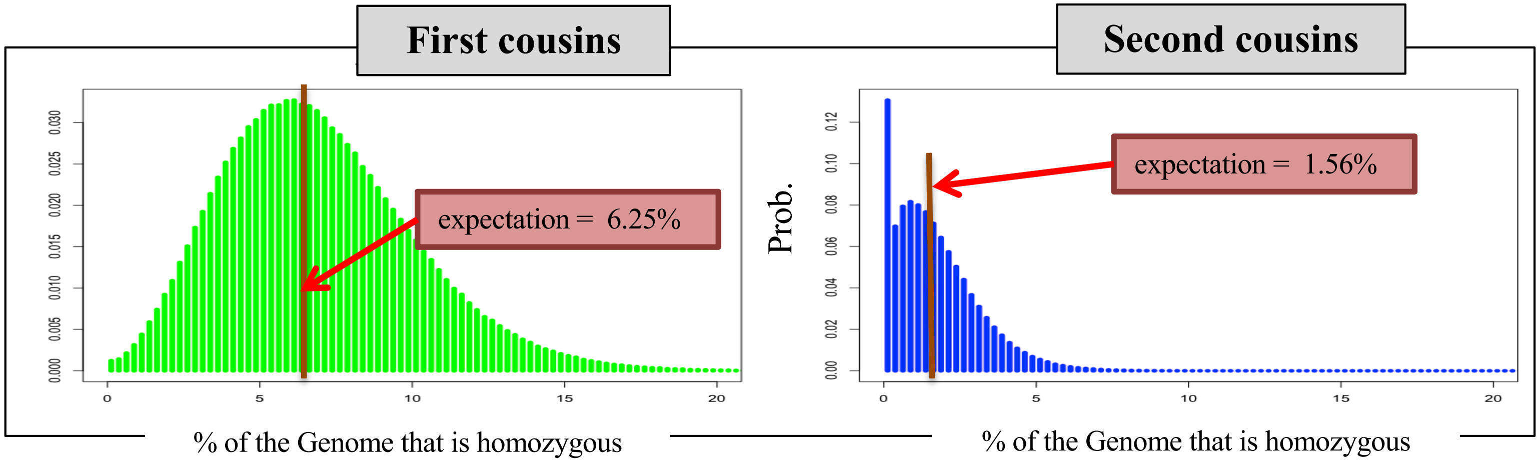

Figure S1: Homozygosity analysis. Theoretical expectation of the percentage of the genome that is homozygous for first and second cousins marriages [Boehnke 1994, Thomas 2007].

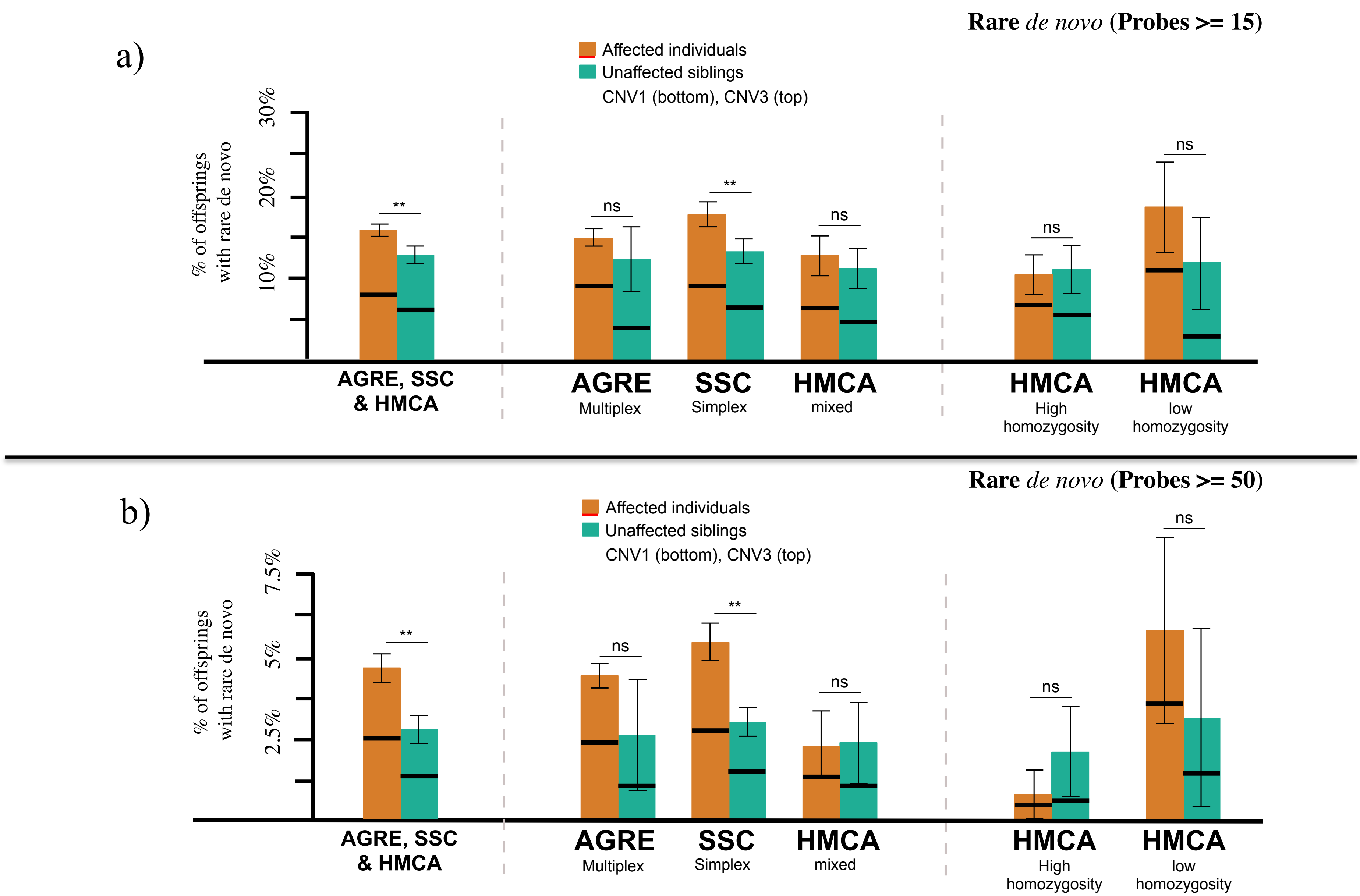

Figure S2: Burden of rare *de novo* events in three ASD collections (CNV1 bottom, CNV3 top). A CNV is considered rare when there is no more than 50% overlapping with any CNV in our HapMap catalogue (Methods: D,E, Variant Explorer pipeline). Statistical evidence and the corresponding number of samples and ratios are shown in Table S2. Error bars represent sampling error.

a) Rare *de novo* deletions and duplications (CNV1 & CNV3, Probes >=15).

b) Rare *de novo* deletions and duplications (CNV1 & CNV3, Probes >=50).

a)

b)

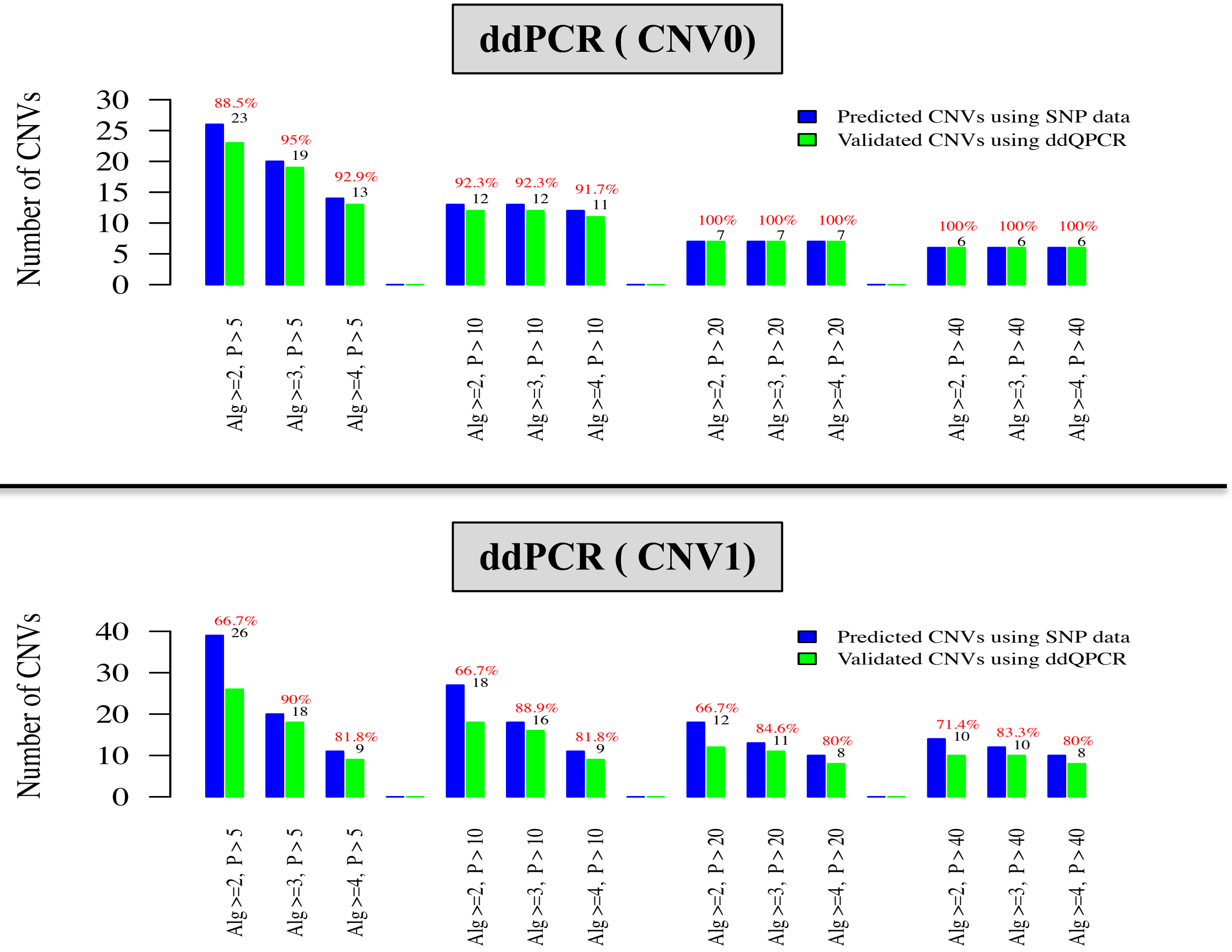

<u>Figure S3</u>: Validation rates using digital droplet PCR (ddPCR). We show validation rates as a function of the number of probes (more than 5, 10, 20, 40), and algorithms identifying the event (≥2, ≥3, =4). As expected, the biallelic deletions produced are easier to identify than those single deletions with a small number of probes. The number of events validated are shown in black and the validation rate in red (Methods: N).

a) Biallelic deletions (CNV0), 26 in total.

b) Single deletions (CNV1), 39 in total.

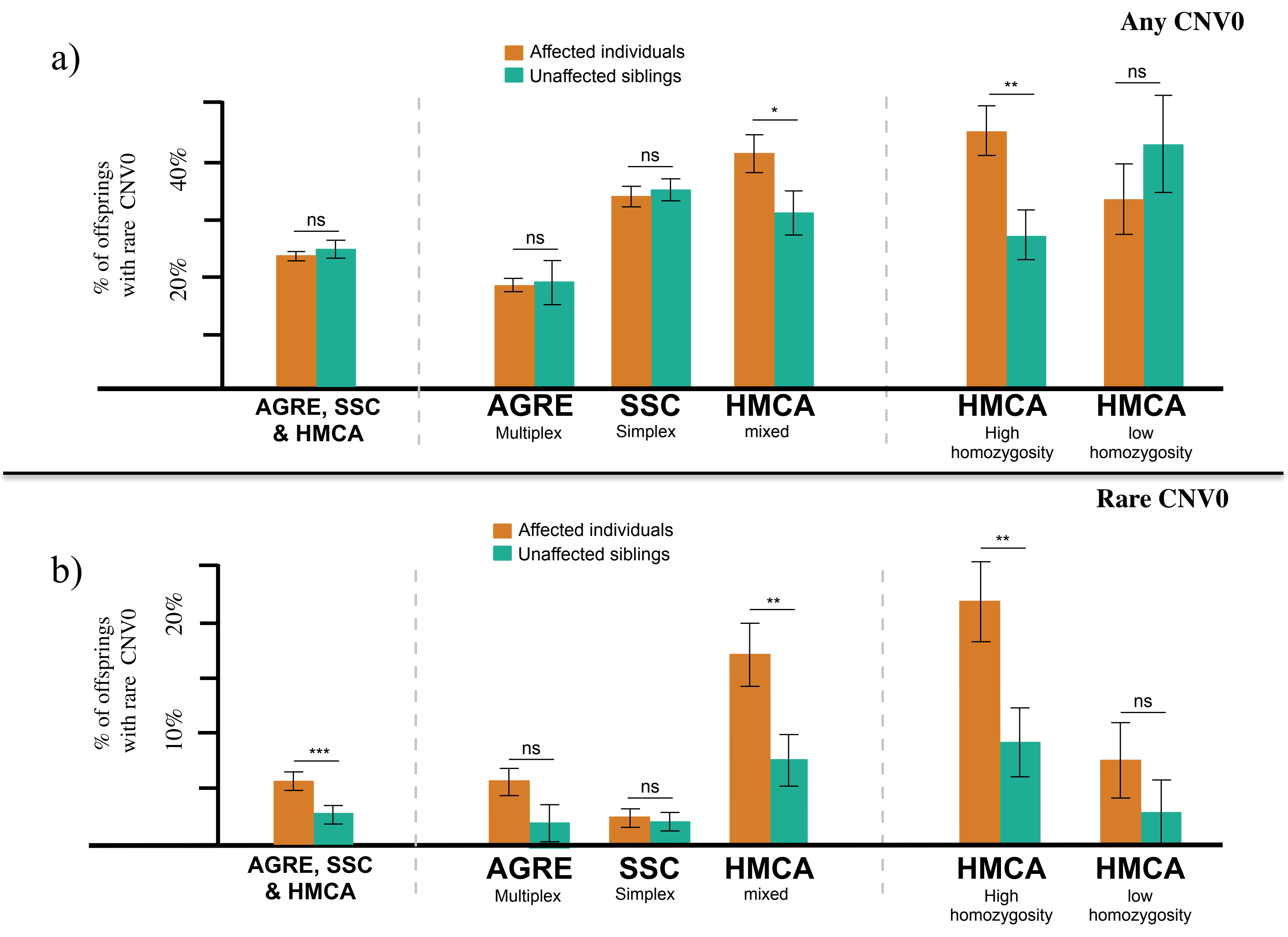

<u>Figure S4</u>: Burden of common and rare biallelic deletions (CNV0) in three ASD collections prior qPCR validation. Statistical evidence and the corresponding number of samples and ratios are shown in Table S4a. Error bars represent sampling error.

a) Any biallelic deletions (common and rare).

b) Rare biallelic deletions.

a)

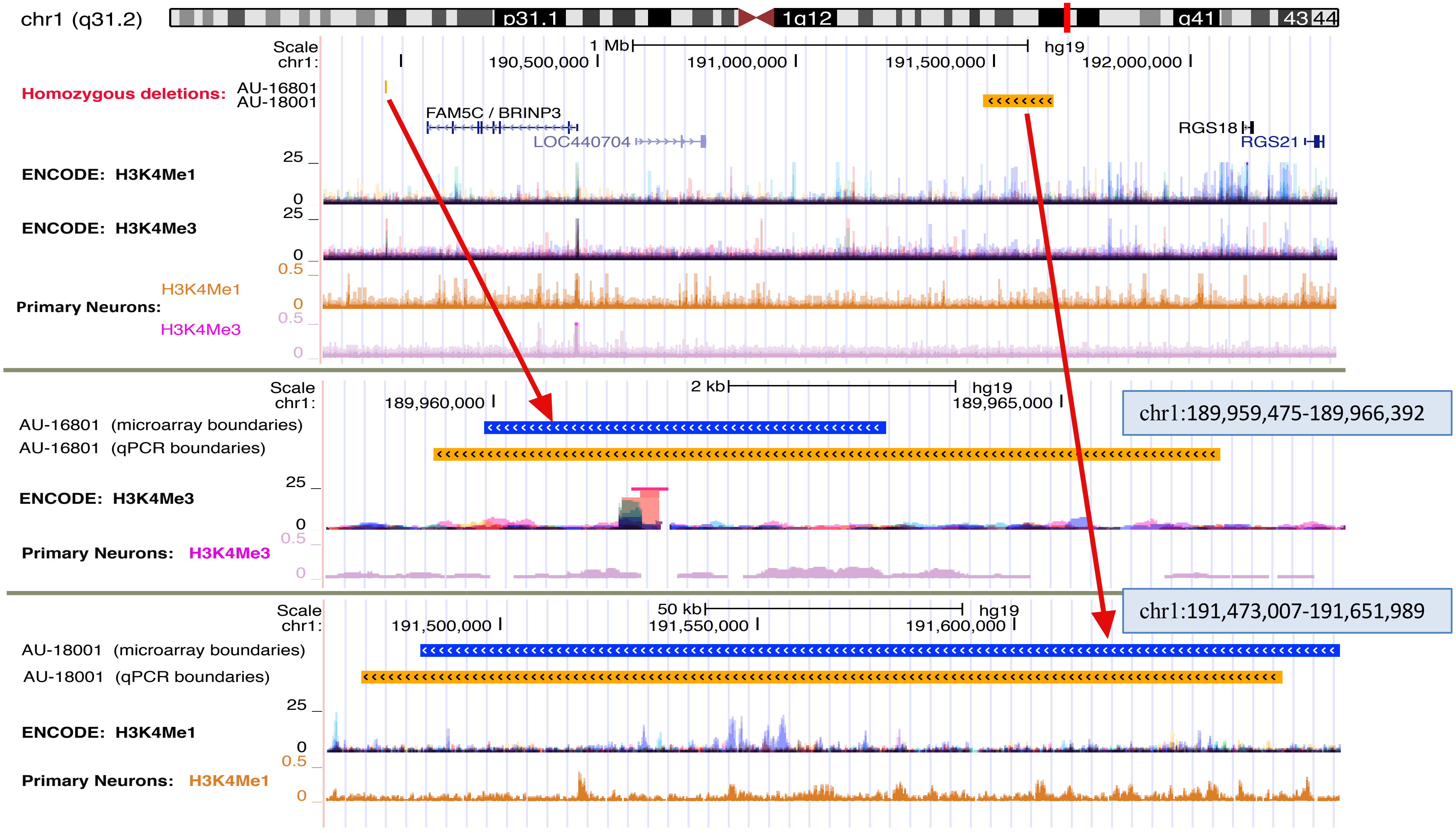

<u>Figures S5a-c</u>: Examples of genes implicated by CNV0s (Table 2). ENCODE or Primary Neuron profiles shown in the figures represents the union all cell lines available.

a) Non-coding biallelic deletions for samples AU-16801 & AU-18001 (***FAM5C/BRINP3***).

b) Non-coding biallelic deletion for sample AU-19503 (***D2HGDH, NEU4, PDCD1***).

c) Exonic biallelic deletion for sample AU-3101 (***C3or58, SLC9A9,*** Morrow et al., 2008).

b)

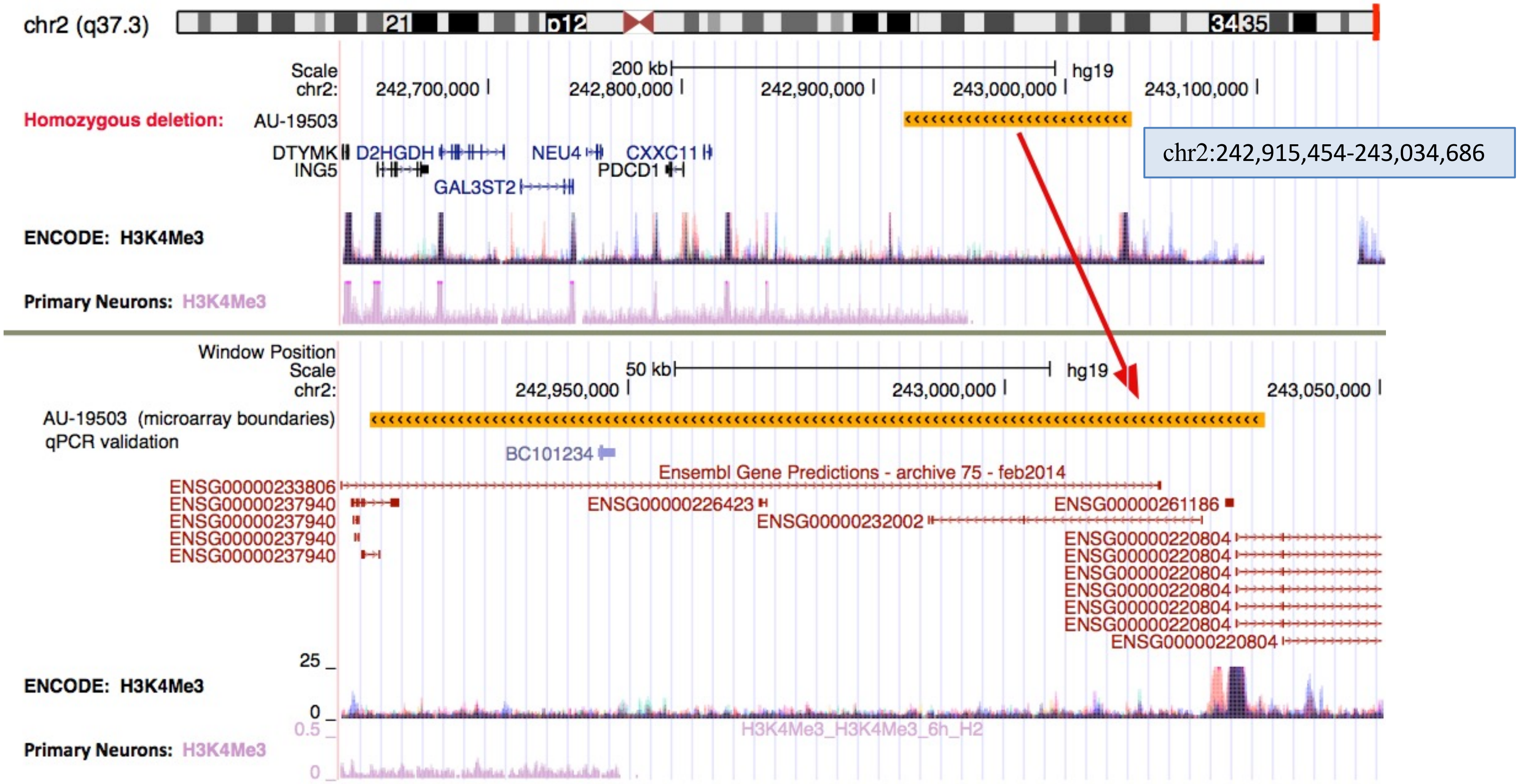
chr2 (q37.3)
Scale
chr2:
200 kb
hg19
242,700,000
242,800,000
242,900,000
243,000,000
243,100,000
Homozygous deletion:
AU-19503
chr2:242,915,454-243,034,686
DTYMK
D2HGDH
NEU4
CXXC11
ING5
PDCD1
GAL3ST2
ENCODE: H3K4Me3
Primary Neurons: H3K4Me3
Window Position
Scale
chr2:
50 kb
hg19
242,950,000
243,000,000
243,050,000
AU-19503 (microarray boundaries)
qPCR validation
BC101234
Ensembl Gene Predictions - archive 75 - feb2014
ENSG00000233806
ENSG00000237940
ENSG00000237940
ENSG00000237940
ENSG00000237940
ENSG00000226423
ENSG00000261186
ENSG00000232002
ENSG00000220804
ENSG00000220804
ENSG00000220804
ENSG00000220804
ENSG00000220804
ENSG00000220804
ENSG00000220804
25
ENCODE: H3K4Me3
0
0.5
H3K4Me3_H3K4Me3_6h_H2
Primary Neurons: H3K4Me3
0

c)

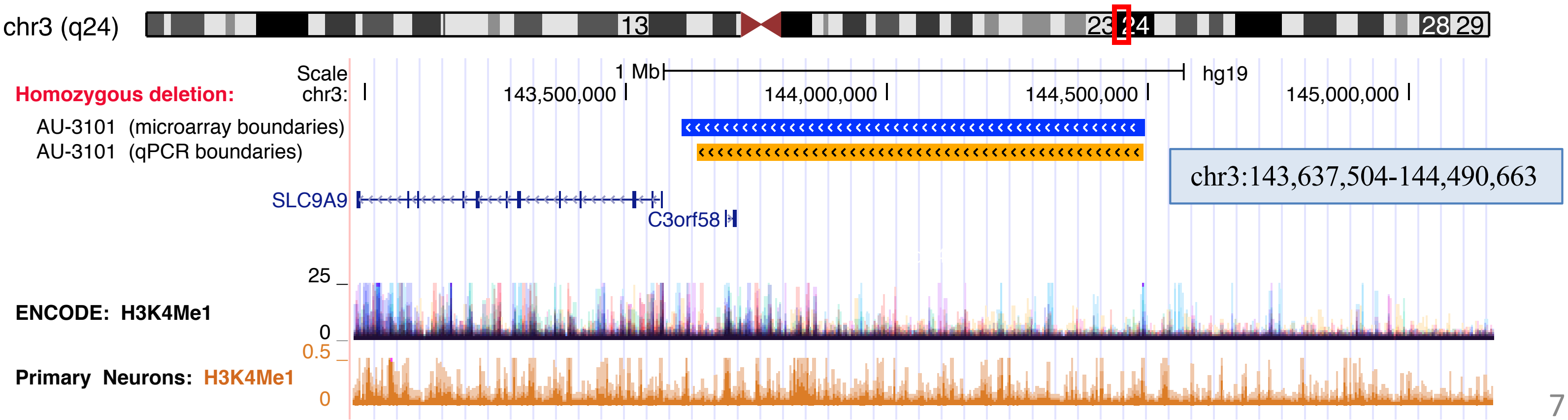
chr3 (q24)
13
23
24
28
29
Scale
chr3:
1 Mb
hg19
143,500,000
144,000,000
144,500,000
145,000,000
Homozygous deletion:
AU-3101 (microarray boundaries)
AU-3101 (qPCR boundaries)
chr3:143,637,504-144,490,663
SLC9A9
C3orf58
25
ENCODE: H3K4Me1
0
0.5
Primary Neurons: H3K4Me1
0

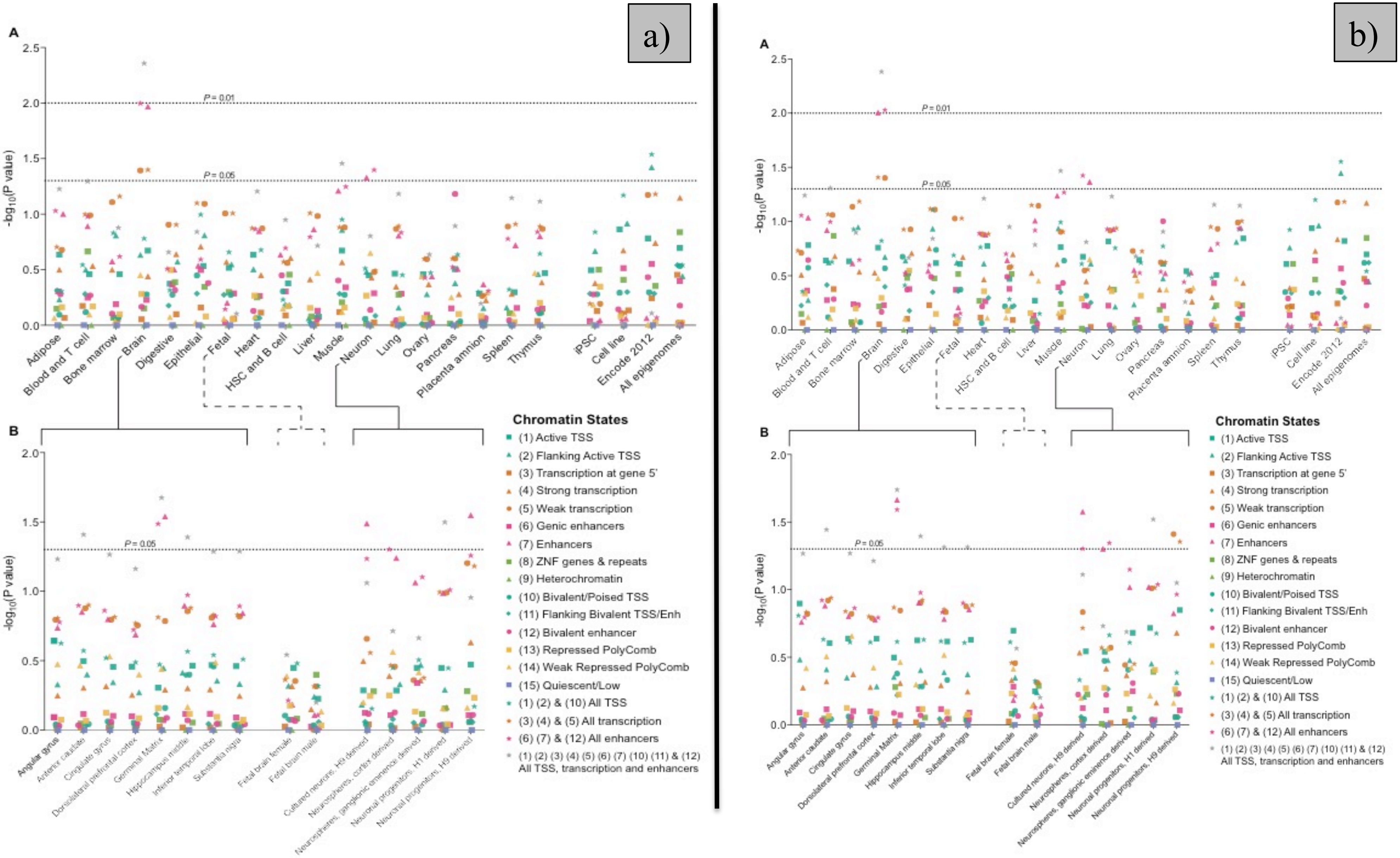

Figures S6a-b: P-values for coincidence between homozygous deletions and epigenetic marks. We use 127 profiles provided by the Epigenome Roadmap Project to test enrichment/depletion of coincidences in affected/unaffected individuals. Epigenetic marks are represented as states of the 15-state ChromHMM model (Ernst J. et al., 2015). For more details about this figure, please see description in Figure 3, Tables S7a-e or Methods: J,M.

a) **Noncoding CNVs defined by RefGene annotation** (Table S7d): 19 CNVs for affected samples and 5 CNVs for unaffected samples.

b) **Noncoding CNVs defined by Ensembl Gene annotation** (Table S7e): 18 CNVs for affected samples and 5 CNVs for unaffected samples.

c) Affymetrix 6.0

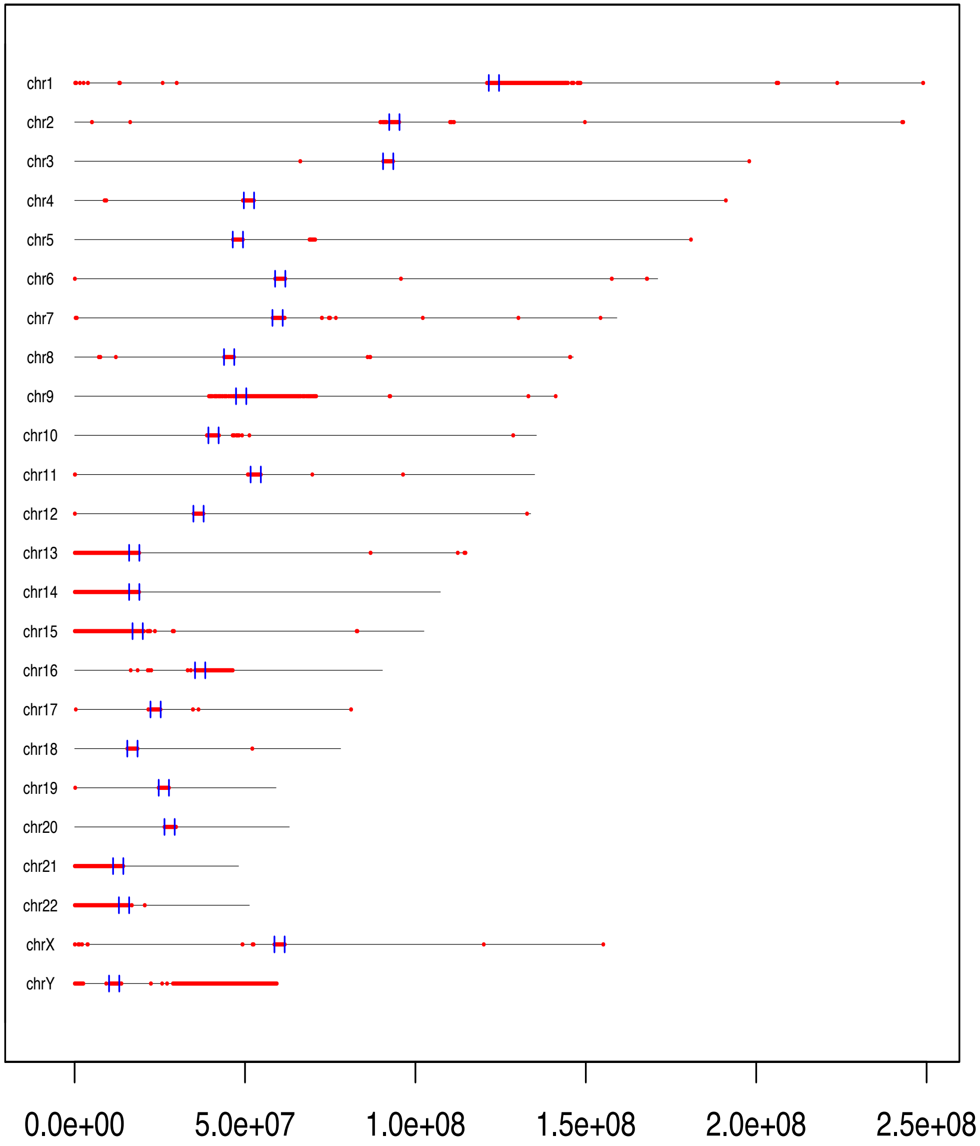

d) Affymetrix 500K

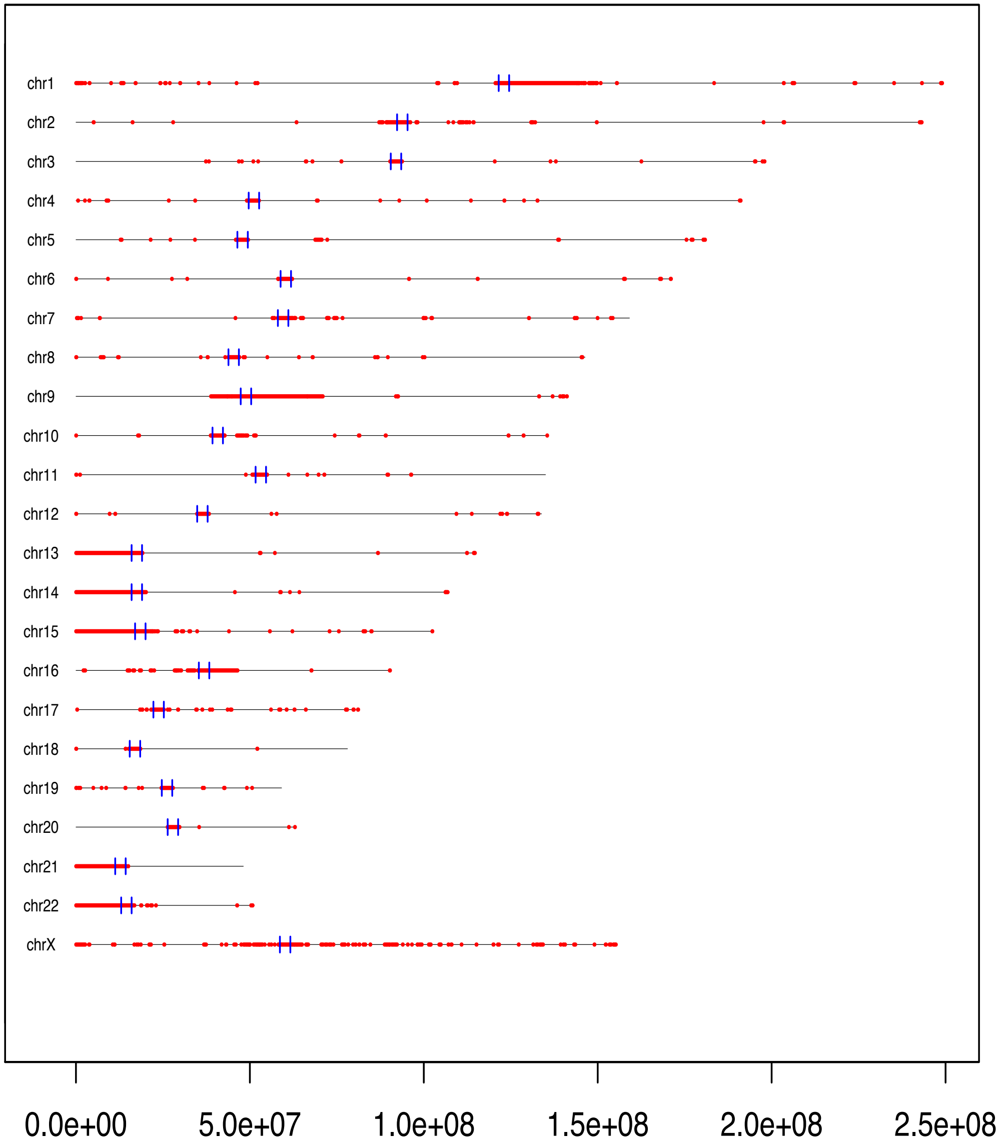

Figures S6c-d: Low marker density regions in SNP arrays (hg19). Affymetrix 500K regions highlighted in red were excluded for Monte-Carlo simulation (Figures 2d & 3 & S6a-b). Blue vertical marks denote centromeres of each chromosome. The Affymetrix 6.0 regions are a subset of the Affymetrix 500K exclusion list (Table S10).

c) **SNP array Affymetrix 6.0:** 175 genomic regions, chromosomes 1-22 (255.12Mb)

d) **SNP array Affymetrix 500K:** 478 genomic regions, chromosomes 1-22 (308.98Mb)

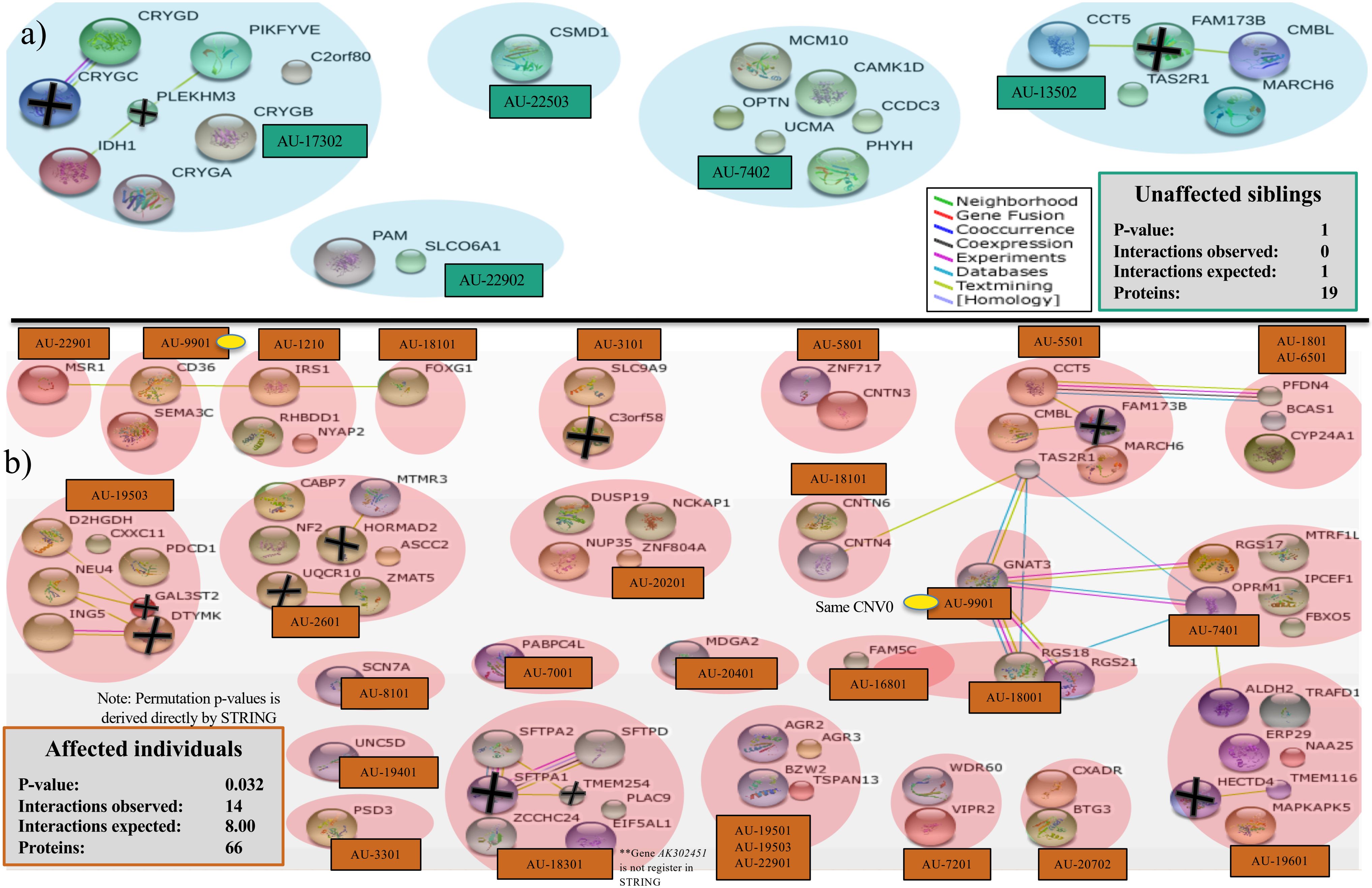

<u>Figures S7a-b</u>: Predicted Protein-Protein Interactions between samples with CNV0s (STRING analysis). To avoid the contribution of interaction from genes clustered near CNVs, we calculated the enrichment of interactions only between subjects. Permutation p-values is derived directly by STRING. Central genes with no external interaction (X) were excluded.

a. **Unaffected siblings:** The network using all 22 genes from unaffected siblings with biallelic deletions (Table S5a, 5 CNV0s) is NOT enriched in interactions (p= NS).

b. **Affected individuals:** The network using all 76 genes from affected individuals with biallelic deletions (Tables 2 or S5a, 28 CNV0s) is enriched in interactions (p<0.03).

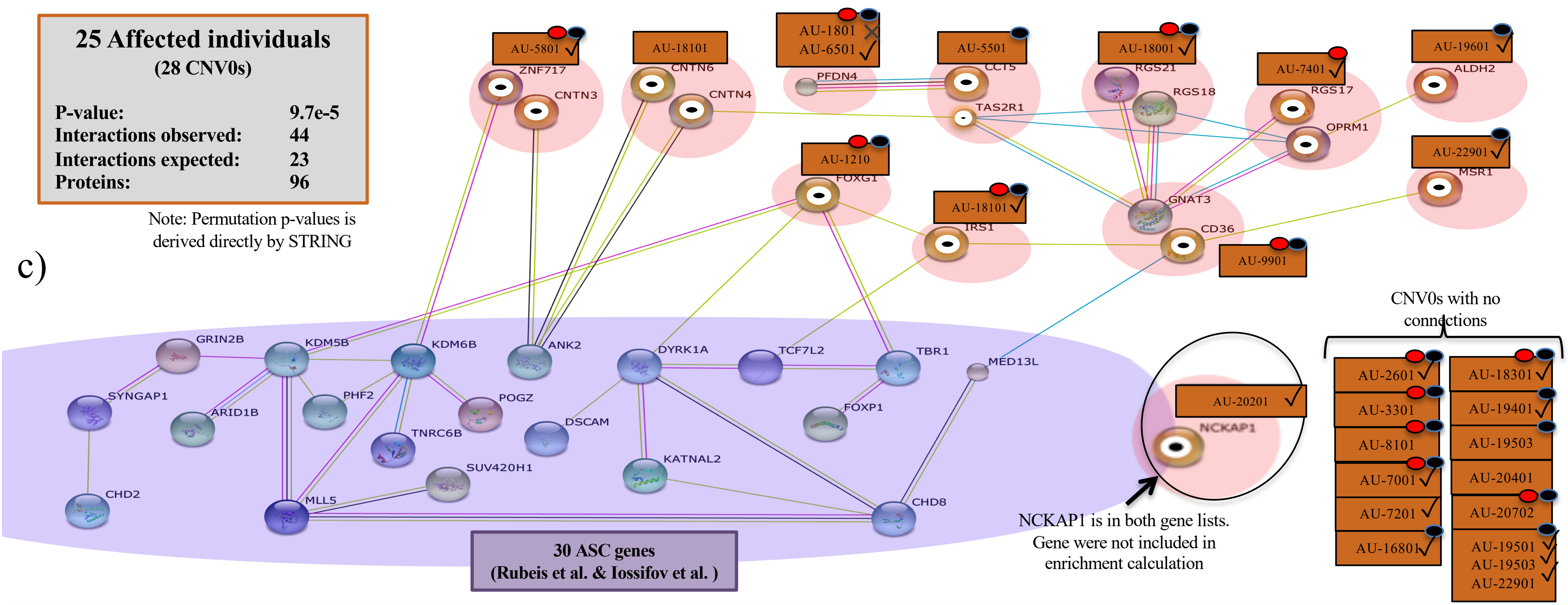

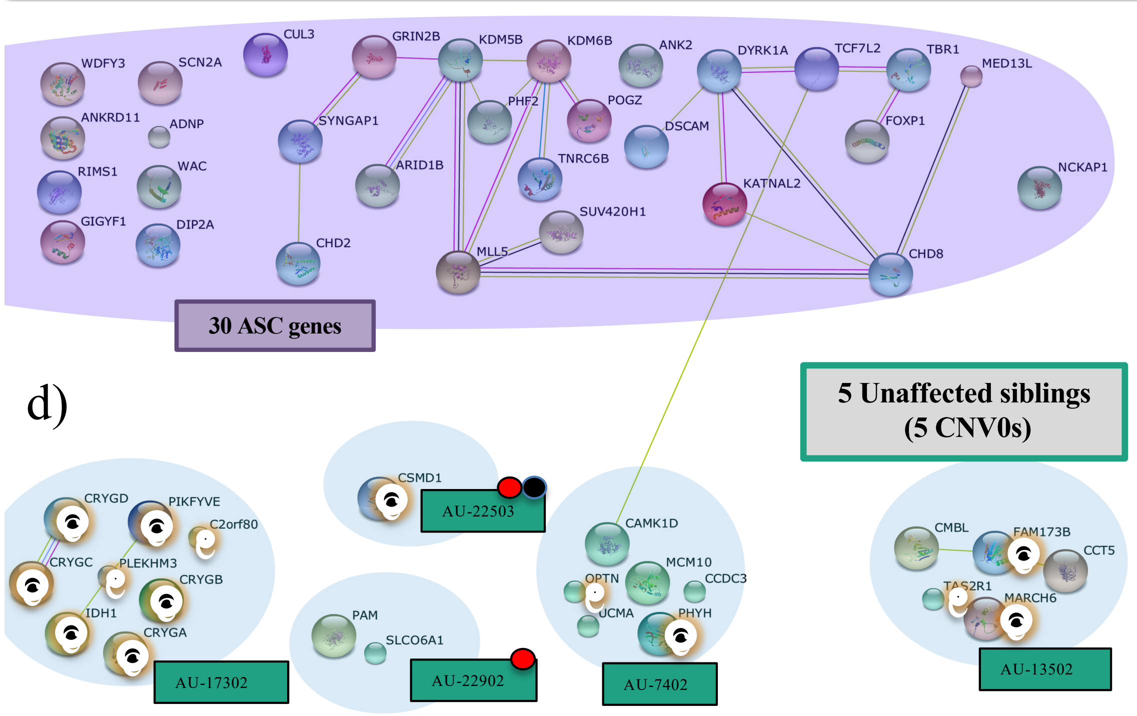

Figures S7c-d: Enrichment calculation for Protein-Protein interactions between samples with CNV0s and the 30 ASC genes (De Rubeis et al. & Iossifov et al.).

c) **Affected individuals:** STRING predicts 44 direct interactions using the 30 ASC genes and 76 genes from affected individuals with qPCR validated biallelic deletions (Figure S7b or Tables 2 or S5a, chromosomes 1-22). The expected number of direct interactions is 23. The network is enriched in interactions (p<6e-5). For display purposes, disconnected genes are excluded from the figure.

d) **Unaffected siblings:** STRING predicts only one interaction between the 30 ASC genes and the 22 genes from unaffected siblings with qPCR validated biallelic deletions (p=NS, Figure S7a or Table S5a, chromosomes 1-22).

- Biallelic deletion with **ENCODE** histone peaks
- Biallelic deletion with **Primary Neuron** histone peaks
- √ Biallelic deletions **segregating** with affection status within families
- Gene **associated with autism** and/or a neurological disease

“Homozygous deletions implicate non-coding epigenetic marks in Autism spectrum disorder “

Klaus Schmitz-Abe, Guzman Sanchez-Schmitz, Ryan N. Doan, R. Sean Hill, Maria Chahrour, Bhaven K. Mehta, Sarah Servattalab, Bulent Ataman, Anh-Thu N. Lam, Eric M. Morrow, Michael E. Greenberg, Timothy W. Yu, Christopher A. Walsh and Kyriacos Markianos

Klaus.Schmitz-Abe@childrens.harvard.edu

Supplemental Information (Tables)

	Type	AGRE & HMCA Affy 6.0 & 5.0 & 500K	AGRE & HMCA Affy 6.0 & 5.0 & 500K	AGRE & HMCA Affy 6.0 & 5.0 & 500K	Simons Simplex Illumina 1M
		CNVs (Alg>=2)	CNVs (Alg>=3)	CNVs (Alg=4)	CNVs
Common CNVs	ALL CNVs	104,101	**36,141**	9,479	**37,361**
	Deletions	69,794	**23,080**	6,232	**24,452**
	Duplications	34,307	**13,061**	3,247	**12,909**
Rare CNVs	ALL CNVs	13,486	**5,136**	1,189	**6,570**
	Deletions	6,184	**3,076**	672	**3,802**
	Duplications	7,302	**2,060**	517	**2,768**
		CNVs/sam (Alg>=2)	CNVs/sam (Alg>=3)	CNVs/sam (Alg=4)	CNVs/sam
Common CNVs	ALL CNVs	27.58	**9.57**	2.51	**9.63**
	Deletions	18.49	**6.11**	1.65	**6.30**
	Duplications	9.09	**3.46**	0.86	**3.33**
Rare CNVs	ALL CNVs	3.57	**1.36**	0.31	**1.69**
	Deletions	1.64	**0.81**	0.18	**0.98**
	Duplications	1.93	**0.55**	0.14	**0.71**
	**Samples**	3,775	3,775	3,775	3,881

Table S1a: CNV call rates as a function of the number of concordant algorithms. Total number of CNVs and the total number of CNVs per sample for the Simons Simplex Collection (SSC), as well as HMCA and AGRE. For the SSC, we used only CNVs with 15 probes or more. Same compatibility is showed when we separately aggregate rates for cases and controls (Table S1b). Throughout the manuscript we perform statistical analysis requiring concordance between 3 of four algorithms. More details about QC and its thresholds filters are available in Methods: A,B,C.

Table S1b: CNV call rates as a function of the number of concordant algorithms (affected vs. unaffected samples). Total number of CNVs and the total number of CNVs for the Simons Simplex Collection (SSC), as well as HMCA and AGRE. For the SSC, we used only CNVs with 15 probes or more. See Table S1a for the join of both affected and unaffected samples.

		Type	AGRE & HMCA Affy 6.0 & 5.0 & 500K	AGRE & HMCA Affy 6.0 & 5.0 & 500K	AGRE & HMCA Affy 6.0 & 5.0 & 500K	Simons Simplex Illumina 1M
Affected individuals			CNVs (Alg>=2)	CNVs (Alg>=3)	CNVs (Alg=4)	CNVs
Affected individuals	Common CNVs	ALL CNVs	45,474	14,952	3,200	10,064
Affected individuals	Common CNVs	Deletions	31,285	9,542	2,095	6,481
Affected individuals	Common CNVs	Duplications	14,189	5,410	1,105	3,583
Affected individuals	Rare CNVs	ALL CNVs	5,695	2,287	428	1,844
Affected individuals	Rare CNVs	Deletions	2,760	1,366	230	1,074
Affected individuals	Rare CNVs	Duplications	2,935	921	198	770
Affected individuals			CNVs/sam (Alg>=2)	CNVs/sam (Alg>=3)	CNVs/sam (Alg=4)	CNVs/sam
Affected individuals	Common CNVs	ALL CNVs	26.21	8.62	1.84	9.80
Affected individuals	Common CNVs	Deletions	18.03	5.50	1.21	6.31
Affected individuals	Common CNVs	Duplications	8.18	3.12	0.64	3.49
Affected individuals	Rare CNVs	ALL CNVs	3.28	1.32	0.25	1.80
Affected individuals	Rare CNVs	Deletions	1.59	0.79	0.13	1.05
Affected individuals	Rare CNVs	Duplications	1.69	0.53	0.11	0.75
Affected individuals		**Samples**	1,735	1,735	1,735	1,027
Unaffected siblings			CNVs (Alg>=2)	CNVs (Alg>=3)	CNVs (Alg=4)	CNVs
Unaffected siblings	Common CNVs	ALL CNVs	58,627	21,189	6,279	27,297
Unaffected siblings	Common CNVs	Deletions	38,509	13,538	4,137	17,971
Unaffected siblings	Common CNVs	Duplications	20,118	7,651	2,142	9,326
Unaffected siblings	Rare CNVs	ALL CNVs	7,791	2,849	761	4,726
Unaffected siblings	Rare CNVs	Deletions	3,424	1,710	442	2,728
Unaffected siblings	Rare CNVs	Duplications	4,367	1,139	319	1,998
Unaffected siblings			CNVs/sam (Alg>=2)	CNVs/sam (Alg>=3)	CNVs/sam (Alg=4)	CNVs/sam
Unaffected siblings	Common CNVs	ALL CNVs	28.74	10.39	3.08	9.56
Unaffected siblings	Common CNVs	Deletions	18.88	6.64	2.03	6.30
Unaffected siblings	Common CNVs	Duplications	9.86	3.75	1.05	3.27
Unaffected siblings	Rare CNVs	ALL CNVs	3.82	1.40	0.37	1.66
Unaffected siblings	Rare CNVs	Deletions	1.68	0.84	0.22	0.96
Unaffected siblings	Rare CNVs	Duplications	2.14	0.56	0.16	0.70
Unaffected siblings		**Samples**	2,040	2,040	2,040	2,854

Table. Inherited mutations identified in ASDs. Severe (nonsense, frameshift) (A) and hypomorphic (missense) (B) mutations in known disease genes were identified in 11 ASD families. M: male, F: female, ID: intellectual disability.

(A) Severe mutations

Mutation	Known disease association	Family	Structure	Consanguinity	# Affected	# Unaffected	Linkage	Primary phenotype	Additional phenotypes
MECP2 p.E483X	Rett syndrome, ASD	AU-5400	Multiplex	No	2 (2M)	-	Yes	Autism	-
NLGN4X p.Q329X	Non-syndromic X-linked ID and/or ASD	AU-5700	Simplex	Yes	1 (M)	1	Yes	Autism	-
PAH p.198_205del	Phenylketonuria	AU-13100	Simplex	Yes	1 (M)	2	Yes	Autism	ID
PAH p.Q235X	Phenylketonuria	AU-4100	Multiplex	Yes	2 (2F)	-	Yes	Autism	-
VPS13B p.A3943fs	Cohen syndrome	AU-21100	Simplex	Yes	1 (M)	3	Yes	Autism	ID, dysmorphic features, hyperextensible joints

(B) Hypomorphic mutations

Mutation	Known disease association	Family	Structure	Consanguinity	#Affected	# Unaffected	Linkage	Primary phenotype	Additional phenotypes
AMT p.I308F	Nonketotic hyperglycinemia	AU-1700	Multiplex	Yes	3 (2M, 1F)	2	Yes	Autism	ID, seizures
AMT p.D198G	Nonketotic hyperglycinemia	AU-11800	Simplex	Yes	1 (M)	1	Yes	Autism	ID, seizures
PEX7 p.W75C	Rhizomelic chondrodysplasia punctata	AU-3500	Multiplex	Yes	3 (2M, 1F)	3	Yes	PDD-NOS	ID, seizures, cataracts
POMGNT1 p.R367H	Muscle–eye–brain disease	AU-13300	Simplex	Yes	1 (M)	1	Yes	Autism	ID
SYNE1 p.L3206M	Autosomal Recessive Cerebellar Ataxia Type 1, Arthrogryposis Congenita, ASD, Bipolar Disease	AU-1600	Multiplex	Yes	4 (1M, 3F)	1	Yes	Autism	ID
VPS13B p.S824A	Cohen syndrome	AU-17800	Simplex	Yes	1 (M)	1	Yes	Autism	ID, dysmorphic features, hyperextensible joints

Table S1c: Likely pathogenic point mutations in the HMCA collection (Yu et al., 2013).

Families in in the HMCA collection underwent whole exome sequencing (WES) to identify inherited causes of autism, resulting in the identification of 5 severe and 6 hypomorphic mutations in 11 families. Overall, whole exome sequencing was performed for 81% of the families in this study.

		Rate of rare *de Novo* CNVs					
		AGRE	SSC	HMCA	HMCA	HMCA	AGRE, SSC and HMCA
	Type	**Multiplex families**	**Simplex families**	**mixed**	**High homozygosity**	**Low homozygosity**	**mixed**
	# of Trios-Cases (Controls)	1240 (80)	1027 (798)	185 (132)	132 (99)	53 (33)	2452 (1010)
Rare *de novo* (P>=15)	Cases ratio	15.0 ± 1.0%	17.9 ± 1.2%	12.9 ± 2.4%	10.6 ± 2.6%	18.8 ± 5.3%	16.1 ± 0.7%
	Control ratio	12.5 ± 3.7%	13.4 ± 1.2%	11.3 ± 2.7%	11.1 ± 3.1%	12.1 ± 5.6%	13.0 ± 1.0%
	**p value**	**NS**	**0.005**	**NS**	**NS**	**NS**	**0.013**
Rare *de novo* (P>=25)	Cases ratio	7.1 ± 0.7%	9.5 ± 0.9%	5.9 ± 1.7%	3.7 ± 1.6%	11.3 ± 4.3%	9.5 ± 0.5%
	Control ratio	5.0 ± 2.4%	5.7 ± 0.8%	5.3 ± 1.9%	5.0 ± 2.2%	6.0 ± 4.1%	5.6 ± 0.7%
	**p value**	0.088	**0.002**	**NS**	**NS**	**NS**	**0.00005**
Rare *de novo* (P>=50)	Cases ratio	4.2 ± 0.5%	5.2 ± 0.7%	2.1 ± 1.0%	0.7 ± 0.7%	5.6 ± 3.1%	4.5 ± 0.4%
	Control ratio	2.5 ± 1.7%	2.8 ± 0.5%	2.2 ± 1.3%	2.0 ± 1.4%	3.0 ± 2.9%	2.7 ± 0.5%
	**p value**	**NS**	**0.008**	**NS**	**NS**	**NS**	**0.009**

Table S2: Fraction of individuals with rare *de novo* CNVs. Micro deletions and duplications (CNV1 & CNV3) not present in parents from the HMCA, AGRE, and SSC collection displayed in Figures 1b and S2 (autosomal chromosomes). Statistical evidence (Fisher test, one sided, one entry per sample) is shown only if they are significant ($p<0.05$). Fractions were calculated prior to validation. In Sanders et al. 2011, 53% of *de novo* predictions based on ≥20 probes experimentally validated.

a)

	Affected individuals	Unaffected siblings
Validate	28	5
Not Validate	12	5
Validation Rate	70%	50%

b)

		Old boundaries			New boundaries			Difference (Kb)
sample	chr	start	end	Size (Kb)	start	end	Size (Kb)	
AU-16801	1	189959917	189963457	3.541	189959475	189966392	6.918	-3.377
AU-18001	1	191484518	191664314	179.797	191473007	191651989	178.983	0.814
AU-18101	2	227342788	227346068	3.281	227341510	227347608	6.099	-2.818
AU-3101	3	143608408	144494787	886.38	143637504	144490663	853.16	33.22
AU-7401	6	154122658	154126883	4.226	154121271	154131343	10.073	-5.847
AU-7201	7	159049679	159064264	14.586	159049219	159062333	13.115	1.471
AU-22901	8	15947142	16022845	75.704	15937585	16026094	88.51	-12.806
AU-19401	8	34805607	34838312	32.706	34800058	34843011	42.954	-10.248
AU-19601	12	112434504	112437514	3.011	112432874	112438514	5.641	-2.63
AU-2601	22	30340356	30369441	29.086	30336496	30366791	30.296	-1.21

Table S3: qPCR validation.

a) **Validation of biallelic deletions with qPCR.**

b) **Refinement of CNV boundaries.** In order to refine the spatial resolution of our CNV predictions, we ran qPCR with probes flanking the predicted boundaries. This analysis was undertaken for 10 of the 28 biallelic deletions presented in Table 2. Overall differences in predicted vs. measured CNV size were very small, in the range of 1-12 kb (except for one large deletion which was predicted at 886kb and measured at 853 kb).

		Homozygous deletions (CNV0)					
		AGRE	SSC	HMCA	HMCA	HMCA	AGRE, SSC and HMCA
	Type	Multiplex families	Simplex families	mixed	High homozygosity	Low homozygosity	mixed
	# of Cases (Controls)	1360 (89)	979 (760)	199 (131)	133 (97)	66 (34)	2538 (980)
CNV0	Cases ratio	17.3 ± 1.0%	32.5 ± 1.5%	39.7 ± 3.4%	43.6 ± 4.3%	31.8 ± 5.7%	24.9 ± 0.8%
	Control ratio	17.9 ± 4.0%	33.6 ± 1.7%	29.7 ± 3.9%	25.7 ± 4.4%	41.1 ± 8.4%	26.7 ± 1.4%
	**p value**	**NS**	**NS**	**0.042**	**0.004**	**NS**	**NS**
Rare CNV0	Cases ratio	5.8 ± 0.6%	2.6 ± 0.5%	17.0 ± 2.6%	21.8 ± 3.5%	7.5 ± 3.2%	5.5 ± 0.4%
	Control ratio	2.2 ± 1.5%	2.2 ± 0.5%	7.6 ± 2.3%	9.2 ± 2.9%	2.9 ± 2.9%	2.9 ± 0.5%
	**p value**	0.107	**NS**	**0.009**	**0.008**	**NS**	**0.0006**
Validate Rare CNV0	Cases ratio			12.5 ± 2.3%	17.2 ± 3.2%	3.03 ± 2.1%	
	Control ratio			3.8 ± 1.6%	4.1 ± 2.0%	2.94 ± 2.9%	
	**p value**			**0.004**	**0.001**	**NS**	

Table S4a: Rate of homozygous deletions (CNV0) from the HMCA, AGRE, and SSC collection in autosomal chromosomes presented in Figures 2b and S4. Statistical evidence, as the Fisher test (one side, one entry per sample) is shown only if they are significant ($p<0.05$).

		Homozygosity correction (CNV0)		
		HMCA	HMCA	HMCA
	Type	**mixed**	**High homozygosity**	**Low homozygosity**
	**Correction ratio**	**1.15**	**1.27**	**1**
Validate Rare CNV0s	% of cases	12.5 ± 2.3%	17.2 ± 3.2%	3.0 ± 2.1%
	% of controls	4.58 ± 1.8%	6.1 ± 2.4%	2.9 ± 2.9%
	**p value**	**0.01**	**0.009**	**NS**

Table S4b: Homozygosity correction in validate rare homozygous deletions.

Adjusting for elevated background rates of homozygosity in affected individuals (Figure 2b and Table S4a). This is a rather simplistic correction since increasing overall homozygosity does not necessarily move a proportional number of individuals from the "not harboring rare CNV0" to the "harboring rare CNV0" category. However it serves as a reasonable estimate of the effect size. Here we increase the number of unaffected progeny that harbor rare CNV0 in proportion to the excess homozygosity observed in affected children.

a)

		sample	Gender	Consan-guineous	Linkage	Homo region	Segre-gation	chr	start	Size (Kb)	ENCODE histone peak	Primary Neuron peak	Protein disrupting	Intronic	Genes on the left	Genes on the right
Autosome chromosomes	Affected individuals	AU-16801	Male	Y	0.93	Y	Y	1	189959475	6.9	High					**FAM5C**
		AU-18001	Male	Y	0.80	Y	Y	1	191473007	179	low	low			**FAM5C**	RGS18, RGS21
		AU-8101*	Male	Y	0.89	Y	Y	2	167346017	47.1	High	High			**SCN7A**	
		AU-20201	Male	Y	0.84	Y	Y	2	184794451	8					DUSP19, **NCKAP1**, NUP35	**ZNF804A**
		AU-18101	Male	Y	0.80	y	Y	2	227341510	6.1	High	low			NYAP2	**IRS1**, RHBDD1
		AU-19503	Male	Y		Y		2	242915454	119.2	High				CXXC11, **D2HGDH**, DTYMK, GAL3ST2, ING5, **NEU4**, **PDCD1**	
		AU-18101	Male	Y				3	1782524	5.1					**CNTN6**	**CNTN4**
		AU-5801*	Male	Y		Y	Y	3	75394265	149.8	High	High			**CNTN3**	**ZNF717**
		AU-3101*	Male	Y	0.89	Y	Y	3	143637504	853.2	High	High	**C3orf58**		**SLC9A9**	
		AU-7001*	Male	Y	0.89	Y	Y	4	134871302	321.4	High	High	**PABPC4L**			
		AU-5501	Male	Y				5	9904421	20.6	High				**TAS2R1**	**CCT5**, CMBL, FAM173B, MARCH6
		AU-7401	Male	Y	0.89	Y	Y	6	154121271	10.1		low			**FBXO5**, MTRF1L, **RGS17**	IPCEF1, **OPRM1**
		AU-19501	Male	Y	1.88	Y	Y	7	16900135	15.3	low		AGR3		AGR2, **BZW2**, **TSPAN13**	
		AU-19503	Male	Y	1.88	Y	Y	7	16900135	15.3	low		AGR3		AGR2, **BZW2**, **TSPAN13**	
		AU-22901	Male	Y	0.85	Y	Y	7	16900135	15.3	low		AGR3		AGR2, **BZW2**, **TSPAN13**	
		AU-9901	Male	Y				7	80157064	141.8	low	low	**CD36**		GNAT3	**SEMA3C**
		AU-7201	Female	Y	0.85	y	Y	7	159049219	13.1		no data			**VIPR2**, **WDR60**	
		AU-22901	Male	Y	0.85	Y	Y	8	15937585	88.5	low		**MSR1**			
		AU-3301	Male	Y		y		8	18852675	9.6	High	low		PSD3		
		AU-19401	Female	Y	0.93	Y	Y	8	34800058	43	High					**UNC5D**
		AU-18301	Female	Y		Y	Y	10	81512254	85.7	High	High			AK302451, EIF5AL1, SFTPA1, SFTPA2, ZCCHC24	PLAC9, SFTPD, **TMEM254**
		AU-19601	Male		0.96	Y	Y	12	112432874	5.6	low			TMEM116	**ALDH2**, **MAPKAPK5**	**ERP29**, HECTD4, NAA25, TRAFD1
		AU-1210	Male	Y		Y		14	28475766	25	low	low				**FOXG1**
		AU-20401	Male	Y		Y		14	47966854	2.6				**MDGA2**		
		AU-1801	Male					20	52643162	20.2	low	low	BCAS1			**CYP24A1**, PFDN4
		AU-6501	Male	Y	0.93	y	Y	20	52643162	20.2	low	low	BCAS1			**CYP24A1**, PFDN4
		AU-20702	Male	Y		Y		21	18802512	19.7	low	High				BTG3, **CXADR**
		AU-2601	Male	Y		Y	Y	22	30336496	30.3	High	High	MTMR3		ASCC2, CABP7, **NF2**, **UQCR10**, ZMAT5	HORMAD2
	Unaffected siblings	AU-17302	Female	Y	na	Y	na	2	208971007	1.8					PLEKHM3	**C2orf80**, **CRYGA**, **CRYGB**, **CRYGC**, **CRYGD**, **IDH1**, **PIKFYVE**
		AU-13502	Female	Y	na		na	5	9905081	16.2					**TAS2R1**	**CCT5**, CMBL, **FAM173B**, **MARCH6**
		AU-22503	Female		na	y	na	5	102044030	10.3	High	High			SLCO6A1	PAM
		AU-22902	Female	Y	na	y	na	8	4717499	88.7		low		**CSMD1**		
		AU-7402	Male	Y	na	y	na	10	13056103	5					CAMK1D, CCDC3	MCM10, **OPTN**, **PHYH**, UCMA

* = publish in 2008 (Morrow, E. M., et al.)

Table S5a: Rare homozygous deletions (CNV0) observed in affected individuals and unaffected siblings in the HMCA collection after validation using qPCR. The list also include homozygosity information for each sample.

b)

Autosome chromosomes

Family	Affected (unaffected)	Consan-guineous	Linkage	Homo region	chr	start	Size (Kb)	ENCODE histone peak	Primary Neuron peak	Protein disrupting	Intronic	Genes on the left	Genes on the right
AU-16800	1 (0)	Y	0.93	Y	1	189959475	6.9	High					***FAM5C***
AU-18000	1 (0)	Y	0.80	Y	1	191473007	178.9	low	low			***FAM5C***	*RGS18*, *RGS21*
AU-8100*	1 (2)	Y	0.89	Y	2	167346017	47	High	High			***SCN7A***	
AU-20200	1 (1)	Y	0.84	Y	2	184794451	7.9					*DUSP19*, ***NCKAP1***, *NUP35*	***ZNF804A***
AU-18100	1 (0)	Y	0.80	y	2	227341510	6.1	High	low			*NYAP2*	***IRS1***, *RHBDD1*
AU-5800*	1 (2)	Y	na	Y	3	75394265	149.8	High	High			***CNTN3***	***ZNF717***
AU-3100*	1 (2)	Y	0.89	Y	3	143637504	853.1	High	High	***C3orf58***		***SLC9A9***	
AU-7000*	1 (2)	Y	0.89	Y	4	134871302	321.3	High	High	***PABPC4L***			
AU-7400	1 (2)	Y	0.89	Y	6	154121271	10		low			***FBXO5***, *MTRF1L*, ***RGS17***	*IPCEF1*, ***OPRM1***
AU-19500	1 (1) Aff0	Y	1.88	Y	7	16900135	15.3	low		*AGR3*		*AGR2*, ***BZW2***, ***TSPAN13***	
AU-22900	1 (1)	Y	0.85	Y	7	16900135	15.3	low		*AGR3*		*AGR2*, ***BZW2***, ***TSPAN13***	
AU-7200	1 (1)	Y	0.85	y	7	159049219	13.1		no data			***VIPR2***, ***WDR60***	
AU-22900	1 (1)	Y	0.85	Y	8	15937585	88.5	low		***MSR1***			
AU-19400	1 (0) Aff0	Y	0.93	Y	8	34800058	42.9	High					***UNC5D***
AU-18300^	1 (2)	Y	na	Y	10	81512254	85.6	High	High			*AK302451*, *EIF5AL1*, *SFTPA1*, *SFTPA2*, *ZCCHC24*	*PLAC9*, *SFTPD*, ***TMEM254***
AU-19600	1 (1)		0.96	Y	12	112432874	5.6	low			*TMEM116*	***ALDH2***, ***MAPKAPK5***	***ERP29***, *HECTD4*, *NAA25*, *TRAFD1*
AU-6500	1 (0)	Y	0.93	y	20	52643162	20.1	low	low	*BCAS1*			***CYP24A1***, *PFDN4*
AU-2600^	1 (0)	Y	na	Y	22	30336496	30.2	High	High	*MTMR3*		*ASCC2*, *CABP7*, ***NF2***, ***UQCR10***, *ZMAT5*	*HORMAD2*

Aff0 = affection is different to the proband

^ = Families with one of the parents not available

* = publish in 2008 (Morrow, E. M., et al.)

Table S5b: Homozygous deletions that segregate within families and are never observed in unaffected subjects. Validated autosomal biallelic deletions segregate perfectly with disease in 17 families from Table 2 (18 affected individuals). In 14 of those (15 affecteds), the deletions interrupt ENCODE histone peaks.

Not Validated Rare CNV0s — ENCODE histone peak threshold

	HMCA						HMCA (High homozygosity)					
ENCODE histone peak threshold	Affected / unaffected	Affected individuals (>=)	Unaffected siblings (>=)	Unaffected siblings (>)	Unaffected siblings (<=)	p value	Affected / unaffected	Affected individuals (>=)	Unaffected siblings (>=)	Unaffected siblings (>)	Unaffected siblings (<=)	p value
	AGRE						SSC					
	82 / 2						27 / 20					
20	42 / 1	99.57%	94.08%	48.18%	51.82%	0.516	11 / 12	99.83%	70.07%	50.77%	49.23%	0.491
25	32 / 1	99.99%	90.38%	38.84%	61.16%	0.611	10 / 11	99.49%	63.97%	44.32%	55.68%	0.554
30	32 / 1	99.67%	86.06%	30.94%	69.06%	0.688	9 / 10	99.03%	60.52%	40.64%	59.36%	0.587
35	31 / 1	98.44%	82.36%	26.1%	73.9%	0.727	5 / 5	99.99%	99.18%	93.67%	6.33%	0.063
40	26 / 0	99.77%	100%	78.77%	21.23%	0.211	5 / 3	99.93%	99.94%	89.13%	10.87%	0.108

Validated Rare CNV0s — ENCODE histone peak threshold

ENCODE histone peak threshold	HMCA						HMCA (High homozygosity)					
	28 / 5						26 / 4					
20	23 / 1	12.21%	99.52%	91.85%	8.15%	0.009	21 / 0	21.35%	100%	98.95%	1.05%	0.002
25	20 / 1	30.86%	98.73%	85.18%	14.82%	0.045	18 / 0	48.06%	100%	97.59%	2.41%	0.011
30	19 / 1	24.27%	97.48%	77.27%	22.73%	0.055	17 / 0	42%	100%	95.65%	4.35%	0.018
35	17 / 1	37.69%	96.01%	70.6%	29.4%	0.110	16 / 0	41.43%	100%	93.6%	6.4%	0.026
40	14 / 1	72.56%	94.27%	64.16%	35.84%	0.260	14 / 0	61.7%	100%	91.22%	8.78%	0.054

← Threshold used in Figure 2d

Table S6: Monte Carlo probabilities for biallelic deletions overlapping with ENCODE histone peaks.

The enrichment/depletion of coincidences observed for affected/unaffected individuals (Tables 2 or S5a) is robust to the choice of ENCODE histone peak thresholds (ENCODE histone peaks > 20,25,…,40). We examined ChIP-seq data gathered from 9 cell lines (GM12878, H1 ES, HepG2, HMEC, HSMM, HUVEC, K562, NHEK and NHLF) as part of the ENCODE project (Ernest et al., 2011). We run 100,000 Monte Carlo simulations for each experiment excluded sex chromosomes, centromeres and regions not cover by SNP Arrays (Figure S6d and Table S10). For more details about this table, please see description in Figure 2d or Methods: J,K.

ID	Epigenome name	Group
1	.-adipose-adipose_nuclei	Adipose
2	.-mesench-adipose_derived_mesenchymal_stem_cell_cultured_cells	
3	.-mesench-mesenchymal_stem_cell_derived_adipocyte_cultured_cells	
4	.-blood_&_t-cell-cd3_primary_cells_cord_bi	Blood and T cell
5	.-blood_&_t-cell-cd3_primary_cells_peripheral_uw	
6	.-blood_&_t-cell-cd4+_cd25+_cd127-_treg_primary_cells	
7	.-blood_&_t-cell-cd4+_cd25-_cd45ra+_naive_primary_cells	
8	.-blood_&_t-cell-cd4+_cd25-_cd45ro+_memory_primary_cells	
9	.-blood_&_t-cell-cd4+_cd25-_il17+_pma-ionomcyin_stimulated_th17_primary_cells	
10	.-blood_&_t-cell-cd4+_cd25-_il17-_pma-ionomycin_stimulated_macs_purified_th_primary_cells	
11	.-blood_&_t-cell-cd4+_cd25int_cd127+_tmem_primary_cells	
12	.-blood_&_t-cell-cd4+_cd25-_th_primary_cells	
13	.-blood_&_t-cell-cd4_memory_primary_cells	
14	.-blood_&_t-cell-cd4_naive_primary_cells	
15	.-blood_&_t-cell-cd8_memory_primary_cells	
16	.-blood_&_t-cell-cd8_naive_primary_cells	
17	.-blood_&_t-cell-peripheral_blood_mononuclear_primary_cells	
18	.-mesench-bone_marrow_derived_mesenchymal_stem_cell_cultured_cells	Bone marrow
19	.-mesench-chondrocytes_from_bone_marrow_derived_mesenchymal_stem_cell_cultured_cells	
20	.-brain-brain_angular_gyrus	Brain
21	.-brain-brain_anterior_caudate	
22	.-brain-brain_cingulate_gyrus	
23	.-brain-brain_germinal_matrix	
24	.-brain-brain_hippocampus_middle	
25	.-brain-brain_inferior_temporal_lobe	
26	.-brain-brain_mid_frontal_lobe	
27	.-brain-brain_substantia_nigra	
28	.-digestive-colonic_mucosa	Digestive
29	.-digestive-duodenum_mucosa	
30	.-digestive-esophagus	
31	.-digestive-fetal_intestine_large	
32	.-digestive-fetal_intestine_small	
33	.-digestive-fetal_stomach	
34	.-digestive-gastric	
35	.-digestive-rectal_mucosa.donor_29	
36	.-digestive-rectal_mucosa.donor_31	
37	.-digestive-sigmoid_colon	
38	.-digestive-small_intestine	
39	.-digestive-stomach_mucosa	

Table S7a: 127 epigenomes (ChromHMM v1.10, Ernst J. et al., 2015) and study group definitions. The 15 ChromHMM states are derived using the core set of 5 chromatin marks assayed in 127 epigenomes (H3K4me3, 216H3K4me1, H3K36me3, H3K27me3, H3K9me3).

ID	Epigenome name	Group
40	.-epithelial-breast_myoepithelial_cells	Epithelial
41	.-epithelial-breast_vhmec	
42	.-epithelial-penis_foreskin_fibroblast_primary_cells_skin01	
43	.-epithelial-penis_foreskin_fibroblast_primary_cells_skin02	
44	.-epithelial-penis_foreskin_keratinocyte_primary_cells_skin02	
45	.-epithelial-penis_foreskin_keratinocyte_primary_cells_skin03	
46	.-epithelial-penis_foreskin_melanocyte_primary_cells_skin01	
47	.-epithelial-penis_foreskin_melanocyte_primary_cells_skin03	
48	.-brain-fetal_brain_female	Fetal
49	.-brain-fetal_brain_male	
50	.-other-fetal_adrenal_gland	
51	.-other-fetal_kidney	
52	.-other-fetal_lung	
53	.-other-fetal_placenta	
54	.-heart-aorta	Heart
55	.-heart-fetal_heart	
56	.-heart-left_ventricle	
57	.-heart-right_atrium	
58	.-heart-right_ventricle	
59	.-hsc_&_b-cell-cd14_primary_cells	HSC and B cell
60	.-hsc_&_b-cell-cd15_primary_cells	
61	.-hsc_&_b-cell-cd19_primary_cells_cord_bi	
62	.-hsc_&_b-cell-cd19_primary_cells_peripheral_uw	
63	.-hsc_&_b-cell-cd34_cultured_cells	
64	.-hsc_&_b-cell-cd34_primary_cells	
65	.-hsc_&_b-cell-cd56_primary_cells	
66	.-hsc_&_b-cell-mobilized_cd34_primary_cells_female	
67	.-hsc_&_b-cell-mobilized_cd34_primary_cells_male	
68	.-other-adult_liver	Liver
69	.-muscle-fetal_muscle_leg	Muscle
70	.-muscle-fetal_muscle_trunk	
71	.-muscle-psoas_muscle	
72	.-muscle-skeletal_muscle_female	
73	.-muscle-skeletal_muscle_male	
74	.-myosat-muscle_satellite_cultured_cells	
75	.-sm._muscle-colon_smooth_muscle	
76	.-sm._muscle-duodenum_smooth_muscle	
77	.-sm._muscle-rectal_smooth_muscle	
78	.-sm._muscle-stomach_smooth_muscle	
79	.-neurosph-neurosphere_cultured_cells_cortex_derived	Neuron
80	.-neurosph-neurosphere_cultured_cells_ganglionic_eminence_derived	
81	.-es-deriv-h1_derived_neuronal_progenitor_cultured_cells	
82	.-es-deriv-h9_derived_neuronal_progenitor_cultured_cells	
83	.-es-deriv-h9_derived_neuron_cultured_cells	
84	.-other-lung	Lung
85	.-other-ovary	Ovary
86	.-other-pancreas	Pancreas
87	.-other-pancreatic_islets	
88	.-other-placenta_amnion	Placenta amnion
89	.-other-spleen	Spleen

ID	Epigenome name	Group
90	.-thymus-fetal_thymus	Thymus
91	.-thymus-thymus	
92	.-ipsc-ips-15b_cell_line	iPSC
93	.-ipsc-ips-18_cell_line	
94	.-ipsc-ips-20b_cell_line	
95	.-ipsc-ips_df_19.11_cell_line	
96	.-ipsc-ips_df_6.9_cell_line	
97	.-esc-4star	Cell line
98	.-esc-es-i3_cell_line	
99	.-esc-es-wa7_cell_line	
100	.-esc-h1_cell_line	
101	.-esc-h9_cell_line	
102	.-esc-hues48_cell_line	
103	.-esc-hues64_cell_line	
104	.-esc-hues6_cell_line	
105	.-es-deriv-h1_bmp4_derived_mesendoderm_cultured_cells	
106	.-es-deriv-h1_bmp4_derived_trophoblast_cultured_cells	
107	.-es-deriv-h1_derived_mesenchymal_stem_cells	
108	.-es-deriv-hesc_derived_cd184+_endoderm_cultured_cells	
109	.-es-deriv-hesc_derived_cd56+_ectoderm_cultured_cells	
110	.-es-deriv-hesc_derived_cd56+_mesoderm_cultured_cells	
111	.-imr90-imr90_cell_line	
112	.-encode2012-a549_etoh_0.02pct_lung_carcinoma	Encode 2012
113	.-encode2012-dnd41_tcell_leukemia	
114	.-encode2012-gm12878_lymphoblastoid	
115	.-encode2012-hela-s3_cervical_carcinoma	
116	.-encode2012-hepg2_hepatocellular_carcinoma	
117	.-encode2012-hmec_mammary_epithelial	
118	.-encode2012-hsmm_skeletal_muscle_myoblasts	
119	.-encode2012-hsmmtube_skeletal_muscle_myotubes_derived_from_hsmm	
120	.-encode2012-huvec_umbilical_vein_endothelial_cells	
121	.-encode2012-k562_leukemia	
122	.-encode2012-monocytes-cd14+_ro01746	
123	.-encode2012-nh-a_astrocytes	
124	.-encode2012-nhdf-ad_adult_dermal_fibroblasts	
125	.-encode2012-nhek-epidermal_keratinocytes	
126	.-encode2012-nhlf_lung_fibroblasts	
127	.-encode2012-osteoblasts	

**ENCODE UCSC**
H3K4Me1, H3K4Me3, H3K27Ac histone marks
**hg19**
GM12878
**HepG2***
**HMEC**
HSMM
HUVEC
K562
NHEK
NHLF
H1-hESC

* H3K4Me1 mark not available

HepG2 & HMEC → We used LiftOver from UCSC (hg18 to hg19)

				All	Encode	Neuron	Brain															
	CNV	Sample	Size (Kb)	Number of epigenomes Ids (127 in total)	Number of epigenomes Ids (16 in total)	Neuron epigenomes Ids (5 in total)	Brain epigenomes Ids (8 in total)	(1) Active TSS	(2) Flanking Active TSS	(3) Transcription at gene 5' & 3'	(4) Strong transcription	(5) Weak transcription	(6) Genic enhancers	(7) Enhancers	(8) ZNF genes & repeats	(9) Heterochromatin	(10) Bivalent/Poised TSS	(11) Flanking Bivalent TSS/Enh	(12) Bivalent enhancer	(13) Repressed PolyComb	(14) Weak Repressed PolyComb	(15) Quiescent/Low
Affected individuals	1	AU-16801	6.9																			
	2	AU-18001	179.0	66	7	79,80,81,82,83	20	1														
	3	AU-8101	47.1	105	14	79,80,81,82,83	20,21,22,23,24,25,26,27	7	3					2			2	1			4	
	4	AU-20201	8.0	6		83																
	5	AU-18101	6.1	79	12	81,82,83	21							1								
	6	AU-19503	119.2	127	16	79,80,81,82,83	20,21,22,23,24,25,26,27	8	3		1	8		8			1			1	7	
	7	AU-18101	5.1																			
	8	AU-5801	149.8	127	16	79,80,81,82,83	20,21,22,23,24,25,26,27	8	6		5	8		8	1		1	1			3	
	9	AU-3101	853.2	127	16	79,80,81,82,83	20,21,22,23,24,25,26,27	8	7			7	2	8	2		1					
	10	AU-7001	321.4	113	16	79,80,81,82,83	20,21,22,23,24,25,26,27	6						2			5	1		3	7	
	11	AU-5501	20.6	42	10	82,83																
	12	AU-7401	10.1	9			23							1								
	13	AU-19501	15.3	28	5																	
	14	AU-19503	15.3	28	5																	
	15	AU-22901	15.3	28	5																	
	16	AU-9901	141.8	98	14	79,80,81,82,83	20,21,24,26,27							3							3	
	17	AU-7201	13.1	35	4		20,21,22,24,26														5	
	18	AU-22901	88.5	30	7	82,83																
	19	AU-3301	9.6	97	14	79,80,81,82,83	21,23,25,26,27					1		5							1	
	20	AU-19401	43.0	36	3	79,80,81,82,83	23,25							1							1	
	21	AU-18301	85.7	126	16	79,80,81,82,83	20,21,22,23,24,25,26,27	8	2		1	4		2								
	22	AU-19601	5.6	74	7	82,83	20,21,23,24,25,26,27					7		1								
	23	AU-1210	25.0	12		82,83	23							1								
	24	AU-20401	2.6	1																		
	25	AU-1801	20.2	73	12	79,80,82	20,21,22,23,24,25,26,27	7	2			3		7								
	26	AU-6501	20.2	73	12	79,80,82	20,21,22,23,24,25,26,27	7	2			3		7								
	27	AU-20702	19.7	76	12	79,80,81,82,83	20,21,22,23,24,25,26,27		4			8		8								
	28	AU-2601	30.3	121	15	79,80,82,83	20,21,22,23,24,25,26,27					8		8								
Unaffected siblings	1	AU-17302	1.8	59	9	82	24,27														2	
	2	AU-13502	16.2	24	6																	
	3	AU-22503	10.3	47	8	82,83																
	4	AU-22902	88.7	32	2	79,80,83																
	5	AU-7402	5.0	55	12	82,83																

Table S7b: Brain and Neuron epigenomes overlapping homozygous deletions (Tables 2 or S5a).

For brain epigenomes, we present 15 states obtained from ChromHMM model (Ernst J. et al., 2015), which is based on a multivariate Hidden Markov Model. Corresponding IDs are shown in Table S7a.

c) Epigenome(s)	# Roadmap files	Cases with p<0.05	(1) Active TSS	(2) Flanking Active TSS	(3) Transcription at gene 5' & 3'	(4) Strong transcription	(5) Weak transcription	(6) Genic enhancers	(7) Enhancers	(8) ZNF genes & repeats	(9) Heterochromatin	(10) Bivalent/ Poised TSS	(11) Flanking Bivalent TSS/Enh	(12) Bivalent enhancer	(13) Repressed PolyComb	(14) Weak Repressed PolyComb	(15) Quiescent/ low	(1) (2) & (10) All TSS	(3) (4) & (5) All transcription	(6) (7) (12) All enhancers	(1) (2) (3) (4) (5) (6) (7)(10)(11) & (12) All TSS, transcription and enhancers
Brain	8	5	0.2200	0.2120	0.8840	0.3640	0.0398	0.5910	0.0087	0.7025	1	0.5350	0.5390	0.6827	0.5250	0.3595	1	0.1461	0.0390	0.0083	0.0038
Neuron	2	2	0.3675	0.3366	0.9390	0.3751	0.2882	0.5280	0.0403	0.9705	1	0.8745	0.8225	0.7279	0.5410	0.2235	1	0.3122	0.2819	0.0356	0.1519
Heart	5	0	0.2445	0.3197	0.7705	0.3548	0.1307	0.5455	0.1237	0.5610	1	0.8640	0.8620	0.7438	0.5405	0.8595	1	0.2166	0.1297	0.1210	0.0601
Lung	1	0	0.3585	0.6959	0.9735	0.4533	0.1378	0.5320	0.1674	0.5385	1	0.9830	0.9600	0.9022	0.8845	0.9930	1	0.3665	0.1296	0.1543	0.0679
Liver	1	0	0.8500	0.8402	0.9605	0.3895	0.0774	0.7380	0.0870	0.8305	1	0.9820	0.9455	0.8757	0.7000	0.3685	1	0.7379	0.0761	0.1073	0.1580
Spleen	1	0	0.5090	0.6934	0.9555	0.5226	0.1308	0.6985	0.1911	0.7785	1	0.9810	0.9480	0.9014	0.9360	0.7865	1	0.4803	0.1242	0.1671	0.0716
Bone marrow	2	0	0.3575	0.1081	0.8860	0.3253	0.0737	0.6475	0.2393	0.8510	1	0.8525	0.8540	0.7875	0.7010	0.8375	1	0.1107	0.0657	0.2228	0.1252
Pancreas	2	0	0.3185	0.4203	0.9705	0.5264	0.2381	0.7035	0.2915	0.9635	1	0.9250	0.9910	0.0684	0.7910	0.4535	1	0.2421	0.2370	0.2822	0.1203
Muscle	10	1	0.2210	0.1459	0.7610	0.2712	0.1375	0.4555	0.0769	0.6215	1	0.5410	0.5290	0.3952	0.6930	0.6915	1	0.1151	0.1367	0.0672	0.0382
Ovary	1	0	0.3590	0.5338	0.9885	0.4319	0.2839	0.8070	0.2706	0.8590	1	0.9695	0.9700	0.9507	0.8640	0.3640	1	0.3449	0.2802	0.2875	0.2078
Placenta amnion	1	0	0.5245	0.7007	0.9905	0.5945	0.5576	0.9075	0.4879	0.9830	1	0.9625	0.9225	0.8634	0.8270	0.5180	1	0.5072	0.5501	0.5500	0.6700
Fetal	6	0	0.3090	0.2871	0.8705	0.2733	0.0908	0.5160	0.5912	0.5075	1	0.5415	0.5400	0.9512	0.8915	0.9065	1	0.2210	0.0903	0.6607	0.7576
Digestive	12	0	0.4640	0.2709	0.7940	0.2315	0.1174	0.4265	0.2822	0.4115	1	0.5400	0.6655	0.4824	0.3190	0.7705	1	0.5128	0.1170	0.2827	0.2107
Epithelial	8	0	0.2975	0.1673	0.6935	0.1971	0.0764	0.3265	0.2758	0.4540	1	0.3710	0.5260	0.4190	0.8645	0.9400	1	0.1124	0.0751	0.2464	0.1422
Thymus	2	0	0.3560	0.3637	0.9160	0.4120	0.1427	0.7115	0.1697	0.7630	1	0.7830	0.7795	0.7230	0.6260	0.3395	1	0.2448	0.1345	0.1519	0.0777
Adipose	3	0	0.2425	0.5975	0.8590	0.3182	0.1943	0.5040	0.0851	0.7105	1	0.8045	0.4990	0.5359	0.7040	0.7790	1	0.5139	0.1843	0.0824	0.0557
Blood and T cell	14	0	0.3570	0.3237	0.6700	0.2983	0.1053	0.5645	0.1298	0.2325	1	0.7635	0.5340	0.5381	0.6590	0.7835	1	0.2902	0.1030	0.1056	0.0509
HSC and B cell	9	0	0.4210	0.6614	0.8140	0.2511	0.2778	0.4960	0.2258	0.3660	1	0.5075	0.6600	0.3698	0.6825	0.6040	1	0.6009	0.2700	0.1976	0.1116
iPSC	5	0	0.3255	0.1660	0.7470	0.4178	0.5891	0.9140	0.8615	0.3235	1	0.5335	0.5265	0.4884	0.3650	0.6380	1	0.1073	0.5897	0.8957	0.9312
Cell line	15	0	0.1450	0.0994	0.7355	0.2142	0.7547	0.3120	0.8418	0.4240	1	0.5170	0.5075	0.5881	0.8405	0.9455	1	0.0560	0.7545	0.8874	0.9741
Encode 2012	16	2	0.1685	0.0343	0.5705	0.1865	0.0667	0.2865	0.8526	0.4435	1	0.5040	0.5185	0.3693	0.9125	0.9700	1	0.0270	0.0654	0.8454	0.7745
All epigenomes	127	0	0.2065	0.3478	0.3515	0.0722	0.9395	0.4095	0.9810	0.1660	1	0.2965	0.2915	0.5747	0.9345	0.9235	1	0.2830	0.9398	0.9854	0.9969
Angular gyrus	1	0	0.2445	0.3694	0.9760	0.5648	0.1547	0.8090	0.1750	0.9310	1	0.9140	0.9455	0.9300	0.8420	0.3455	1	0.1536	0.1504	0.1610	0.0536
Anterior caudate	1	1	0.3260	0.3502	0.9555	0.4959	0.1239	0.7700	0.1310	0.8985	1	0.9060	0.9240	0.9239	0.8600	0.3525	1	0.2328	0.1181	0.1194	0.0352
Cingulate gyrus	1	0	0.3600	0.4529	0.9665	0.4900	0.1544	0.7895	0.1579	0.9205	1	0.8660	0.8900	0.8738	0.7495	0.3000	1	0.3065	0.1477	0.1367	0.0537
Germinal Matrix	1	3	0.5020	0.3710	0.9500	0.5114	0.1465	0.8495	0.0169	0.8410	1	0.6925	0.7685	0.8179	0.6930	0.3625	1	0.3448	0.1389	0.0204	0.0163
Hippocampus middle	1	1	0.3530	0.3885	0.9565	0.4998	0.1292	0.7640	0.1247	0.8835	1	0.4100	0.4145	0.8505	0.7625	0.6935	1	0.2746	0.1228	0.1052	0.0399
Inferior temporal lobe	1	1	0.3495	0.4258	0.9560	0.5093	0.1500	0.7620	0.1660	0.9010	1	0.3590	0.9025	0.9026	0.8040	0.3340	1	0.2420	0.1428	0.1471	0.0480
Dorsolateral prefrontal cortex	1	0	0.3590	0.5360	0.9610	0.5585	0.1680	0.7960	0.1710	0.9335	1	0.8830	0.9130	0.9011	0.8220	0.4010	1	0.3315	0.1626	0.1634	0.0613
Substantia nigra	1	1	0.3560	0.4443	0.9710	0.5691	0.1388	0.8155	0.1402	0.9075	1	0.9245	0.9370	0.9092	0.8410	0.8580	1	0.2493	0.1352	0.1254	0.0478
Fetal brain female	1	0	0.3685	0.4850	0.9420	0.4829	0.4661	0.8460	0.6561	0.8265	1	0.7875	0.8395	0.8601	0.6655	0.4125	1	0.3372	0.4532	0.6111	0.2934
Fetal brain male	1	0	0.5935	0.5766	0.9870	0.7079	0.4511	0.9425	0.6898	0.4110	1	0.9260	0.8760	0.9704	0.9285	0.7370	1	0.4799	0.4501	0.7201	0.6220
Neurospheres, cortex derived	1	2	0.5260	0.4722	0.9715	0.4114	0.2780	0.7805	0.0457	0.9840	1	0.9295	0.8365	0.7461	0.5620	0.2730	1	0.3657	0.2784	0.0420	0.1823
Neurospheres, ganglionic eminence derived	1	0	0.3680	0.3492	0.9475	0.4390	0.3760	0.4720	0.0705	0.7650	1	0.9120	0.8845	0.8647	0.7370	0.3835	1	0.3172	0.3674	0.0670	0.2002
Neuronal progenitors, H1 derived	1	1	0.3705	0.5245	0.9840	0.6872	0.0984	0.9190	0.0913	0.7005	1	0.9150	0.9420	0.9248	0.9060	0.6970	1	0.3690	0.0973	0.0876	0.0290
Neuronal progenitors, H9 derived	1	4	0.3540	0.8415	0.8775	0.2488	0.0401	0.7830	0.0141	0.5365	1	0.8750	0.8770	0.4989	0.5835	0.6905	1	0.6973	0.0463	0.0307	0.0779
Cultured neurons, H9 derived	1	2	0.5250	0.4137	0.9090	0.3274	0.1411	0.7530	0.0186	0.5370	1	0.9100	0.8710	0.7639	0.5690	0.6310	1	0.4895	0.1901	0.0361	0.0640

Colour		
(light green)	==	P<=0.05
(yellow)	==	P<=0.04
(orange)	==	P<=0.03
(blue)	==	p<=0.01

Tables S7c-e: P-values for coincidence between homozygous deletions and epigenetic marks. We use 127 profiles provided by the Epigenome Roadmap Project to test enrichment/depletion of coincidences in affected/unaffected individuals. Epigenetic marks are represented as states of the 15-state ChromHMM model (Ernst J. et al., 2015). For more details about this tables, please see description in Figures 3, S6a-b or Methods: J,M.

c) **Noncoding CNVs defined by KnownGene annotation:** 17 CNVs for affected samples and 5 CNVs for unaffected samples (Tables 2, S5a or S9).

d) **Noncoding CNVs defined by RefGene annotation:** 19 CNVs for affected samples and 5 CNVs for unaffected samples (Tables 2, S5a or S9).

e) **Noncoding CNVs defined by Ensembl Gene annotation:** 18 CNVs for affected samples and 5 CNVs for unaffected samples (Tables 2, S5a or S9).

d) Epigenome(s)	# Roadmap files	Cases with p<0.05	(1) Active TSS	(2) Flanking Active TSS	(3) Transcription at gene 5' & 3'	(4) Strong transcription	(5) Weak transcription	(6) Genic enhancers	(7) Enhancers	(8) ZNF genes & repeats	(9) Heterochromatin	(10) Bivalent/ Poised TSS	(11) Flanking Bivalent TSS/Enh	(12) Bivalent enhancer	(13) Repressed PolyComb	(14) Weak Repressed PolyComb	(15) Quiescent/ low	(1) (2) & (10) All TSS	(3) (4) & (5) All transcription	(6) (7) (12) All enhancers	(1) (2) (3) (4) (5) (6) (7)(10)(11) & (12) All TSS, transcription and enhancers
Brain	8	5	0.2125	0.2330	0.8840	0.3621	0.0405	0.5910	0.0108	0.7025	1	0.5275	0.5280	0.6829	0.5250	0.3535	1	0.1653	0.0398	0.0101	0.0044
Neuron	2	2	0.3550	0.3295	0.9390	0.3729	0.3323	0.5145	0.0469	0.9705	1	0.8745	0.8225	0.7278	0.5410	0.2235	1	0.3070	0.3215	0.0399	0.1572
Heart	5	0	0.2325	0.3136	0.7705	0.3533	0.1347	0.5455	0.1424	0.5610	1	0.8640	0.8620	0.7437	0.5405	0.8335	1	0.2093	0.1336	0.1363	0.0621
Lung	1	0	0.3425	0.6961	0.9735	0.4487	0.1359	0.5195	0.1562	0.5285	1	0.9830	0.9600	0.9023	0.8845	0.9930	1	0.3520	0.1280	0.1461	0.0654
Liver	1	0	0.8410	0.8663	0.9605	0.3870	0.1040	0.7380	0.1374	0.8305	1	0.9820	0.9455	0.8756	0.7000	0.3375	1	0.8258	0.0977	0.1600	0.1916
Spleen	1	0	0.5030	0.6933	0.9555	0.5226	0.1292	0.6985	0.1900	0.7785	1	0.9810	0.9480	0.9013	0.9360	0.7865	1	0.4757	0.1228	0.1665	0.0713
Bone marrow	2	0	0.3465	0.1556	0.8860	0.3171	0.0780	0.6475	0.2658	0.8510	1	0.8525	0.8540	0.7875	0.7945	0.9135	1	0.1474	0.0689	0.2398	0.1320
Pancreas	2	0	0.3125	0.4178	0.9705	0.5264	0.2512	0.7035	0.3112	0.9635	1	0.9250	0.9910	0.0658	0.7910	0.5615	1	0.2308	0.2500	0.2989	0.1273
Muscle	10	1	0.2130	0.1387	0.7610	0.2706	0.1319	0.4555	0.0617	0.6215	1	0.5315	0.5210	0.3951	0.6930	0.7610	1	0.1114	0.1311	0.0565	0.0349
Ovary	1	0	0.3480	0.5259	0.9885	0.4282	0.2539	0.8070	0.3633	0.8590	1	0.9695	0.9700	0.9505	0.8640	0.3625	1	0.3363	0.2516	0.3729	0.2302
Placenta amnion	1	0	0.5175	0.7006	0.9905	0.5945	0.5433	0.9075	0.4278	0.9830	1	0.9625	0.9225	0.8632	0.8270	0.5090	1	0.5013	0.5366	0.4892	0.6356
Fetal	6	0	0.3040	0.2833	0.8705	0.2691	0.0986	0.5160	0.6281	0.5015	1	0.5320	0.5310	0.9446	0.8850	0.8710	1	0.2194	0.0980	0.7144	0.7851
Digestive	12	0	0.4235	0.2680	0.7940	0.2292	0.1245	0.4265	0.3159	0.4090	1	0.5300	0.6655	0.4825	0.3180	0.8455	1	0.4814	0.1241	0.3100	0.2194
Epithelial	8	0	0.2925	0.1554	0.6935	0.1958	0.0808	0.3185	0.2911	0.4505	1	0.3565	0.5180	0.4163	0.8305	0.9450	1	0.1007	0.0794	0.2552	0.1440
Thymus	2	0	0.3400	0.3468	0.9160	0.4120	0.1356	0.7115	0.1583	0.7630	1	0.7830	0.7795	0.7228	0.6260	0.3395	1	0.2266	0.1285	0.1442	0.0765
Adipose	3	0	0.2310	0.5863	0.8590	0.3170	0.2095	0.4965	0.0996	0.7105	1	0.8045	0.4870	0.5260	0.6855	0.8620	1	0.4939	0.1974	0.0930	0.0592
Blood and T cell	14	0	0.3470	0.3166	0.6700	0.2921	0.1023	0.5645	0.1284	0.2170	1	0.7635	0.5230	0.5383	0.6770	0.8030	1	0.2857	0.1001	0.1049	0.0505
HSC and B cell	9	0	0.4185	0.6568	0.8140	0.2479	0.2757	0.4960	0.2323	0.3505	1	0.4950	0.6600	0.3554	0.6735	0.7050	1	0.5875	0.2679	0.2013	0.1121
iPSC	5	0	0.3195	0.2149	0.7470	0.4178	0.6398	0.9140	0.9059	0.3170	1	0.5255	0.5195	0.5280	0.4485	0.6530	1	0.1444	0.6397	0.9310	0.9524
Cell line	15	0	0.1370	0.1210	0.7355	0.2123	0.7771	0.3060	0.8679	0.3890	1	0.5105	0.5015	0.7194	0.8280	0.9705	1	0.0675	0.7768	0.9072	0.9797
Encode 2012	16	2	0.1650	0.0378	0.5705	0.1817	0.0671	0.2815	0.8644	0.4435	1	0.5040	0.5185	0.3694	0.9375	0.9835	1	0.0290	0.0658	0.8536	0.7773
All epigenomes	127	0	0.2020	0.3611	0.3515	0.0713	0.9483	0.4000	0.9826	0.1455	1	0.2925	0.2875	0.6648	0.9490	0.9390	1	0.2872	0.9485	0.9867	0.9972
Angular gyrus	1	0	0.2275	0.4701	0.9760	0.5648	0.1605	0.8090	0.1832	0.9310	1	0.9140	0.9455	0.9302	0.8420	0.3350	1	0.2366	0.1559	0.1667	0.0585
Anterior caudate	1	1	0.3190	0.3981	0.9555	0.4959	0.1325	0.7700	0.1409	0.8985	1	0.9060	0.9240	0.9238	0.8600	0.3415	1	0.2677	0.1256	0.1263	0.0389
Cingulate gyrus	1	0	0.3500	0.4529	0.9665	0.4834	0.1600	0.7895	0.1590	0.9205	1	0.8660	0.8900	0.8737	0.7495	0.2945	1	0.3015	0.1528	0.1373	0.0542
Germinal Matrix	1	3	0.4965	0.3579	0.9500	0.5114	0.1638	0.8495	0.0289	0.8410	1	0.6925	0.7685	0.8177	0.6930	0.3625	1	0.3368	0.1536	0.0326	0.0211
Hippocampus middle	1	1	0.3435	0.3867	0.9565	0.4998	0.1390	0.7640	0.1268	0.8835	1	0.3985	0.4020	0.8507	0.7625	0.6800	1	0.2710	0.1313	0.1063	0.0407
Inferior temporal lobe	1	0	0.3405	0.4418	0.9560	0.5093	0.1551	0.7620	0.1727	0.9010	1	0.3480	0.9025	0.9024	0.8040	0.3260	1	0.2875	0.1472	0.1511	0.0515
Dorsolateral prefrontal cortex	1	0	0.3490	0.5358	0.9610	0.5585	0.1760	0.7960	0.2053	0.9335	1	0.8830	0.9130	0.9009	0.8220	0.3980	1	0.3245	0.1699	0.1890	0.0686
Substantia nigra	1	0	0.3465	0.4643	0.9710	0.5691	0.1521	0.8155	0.1438	0.9075	1	0.9245	0.9370	0.9091	0.8410	0.8580	1	0.3081	0.1477	0.1277	0.0513
Fetal brain female	1	0	0.3555	0.4802	0.9420	0.4829	0.4436	0.8460	0.6559	0.8265	1	0.7875	0.8395	0.8600	0.6655	0.4095	1	0.3298	0.4332	0.6110	0.2861
Fetal brain male	1	0	0.5935	0.5765	0.9870	0.7079	0.4849	0.9425	0.7793	0.3985	1	0.9260	0.8760	0.9701	0.9285	0.7345	1	0.4799	0.4836	0.7822	0.6323
Neurospheres, cortex derived	1	1	0.5190	0.4680	0.9715	0.4083	0.3484	0.7805	0.0576	0.9840	1	0.9295	0.8365	0.7462	0.5620	0.2730	1	0.3545	0.3412	0.0498	0.1928
Neurospheres, ganglionic eminence derived	1	0	0.3555	0.3404	0.9475	0.4390	0.4375	0.4575	0.0865	0.7650	1	0.9120	0.8845	0.8649	0.7370	0.3835	1	0.3116	0.4205	0.0789	0.2167
Neuronal progenitors, H1 derived	1	1	0.3565	0.5127	0.9840	0.6872	0.1024	0.9190	0.1028	0.7005	1	0.9150	0.9420	0.9248	0.9060	0.6970	1	0.3569	0.1012	0.0975	0.0316
Neuronal progenitors, H9 derived	1	1	0.3375	0.8308	0.8775	0.2334	0.0626	0.7830	0.0283	0.5255	1	0.8750	0.8770	0.7783	0.5835	0.6905	1	0.6746	0.0657	0.0552	0.1106
Cultured neurons, H9 derived	1	1	0.5175	0.6562	0.9090	0.3190	0.2202	0.7530	0.0325	0.5260	1	0.9100	0.8710	0.7642	0.5690	0.6310	1	0.7001	0.2768	0.0581	0.0868

Colour		
(light green)	==	P<=0.05
(yellow)	==	P<=0.04
(orange)	==	P<=0.03
(blue)	==	p<=0.01

e) Epigenome(s)	# Roadmap files	Cases with p<0.05	(1) Active TSS	(2) Flanking Active TSS	(3) Transcription at gene 5' & 3'	(4) Strong transcription	(5) Weak transcription	(6) Genic enhancers	(7) Enhancers	(8) ZNF genes & repeats	(9) Heterochromatin	(10) Bivalent/ Poised TSS	(11) Flanking Bivalent TSS/Enh	(12) Bivalent enhancer	(13) Repressed PolyComb	(14) Weak Repressed PolyComb	(15) Quiescent/ low	(1) (2) & (10) All TSS	(3) (4) & (5) All transcription	(6) (7) (12) All enhancers	(1) (2) (3) (4) (5) (6) (7)(10)(11) & (12) All TSS, transcription and enhancers
Brain	8	5	0.1740	0.2120	0.8840	0.2969	0.0397	0.5910	0.0099	0.6065	1	0.2700	0.3995	0.6827	0.5080	0.3335	1	0.1503	0.0390	0.0093	0.0041
Neuron	2	2	0.2110	0.2439	0.9390	0.3566	0.2832	0.5965	0.0430	0.9705	1	0.4850	0.5605	0.6025	0.5155	0.2235	1	0.2454	0.2770	0.0375	0.1538
Heart	5	0	0.1680	0.2441	0.7705	0.3421	0.1310	0.5455	0.1321	0.5410	1	0.8640	0.5065	0.6076	0.4405	0.7760	1	0.1721	0.1300	0.1281	0.0611
Lung	1	0	0.1525	0.6110	0.9735	0.4080	0.1213	0.5975	0.1174	0.4255	1	0.9830	0.9600	0.9022	0.4405	0.9385	1	0.1753	0.1151	0.1174	0.0585
Liver	1	0	0.5360	0.8135	0.9605	0.2992	0.0717	0.7380	0.0983	0.8305	1	0.9820	0.9455	0.8757	0.7000	0.3530	1	0.7065	0.0706	0.1204	0.1634
Spleen	1	0	0.3115	0.6087	0.9555	0.5000	0.1168	0.3710	0.1788	0.5950	1	0.9810	0.9480	0.9014	0.9360	0.7865	1	0.3300	0.1119	0.1594	0.0697
Bone marrow	2	0	0.2320	0.1129	0.8860	0.2920	0.0730	0.5810	0.2436	0.8510	1	0.8525	0.8540	0.5889	0.6325	0.8070	1	0.1178	0.0652	0.2256	0.1262
Pancreas	2	0	0.2455	0.3365	0.9705	0.4258	0.2382	0.6000	0.3005	0.6540	1	0.9250	0.9910	0.0994	0.7910	0.4275	1	0.1685	0.2371	0.2898	0.1235
Muscle	10	1	0.1725	0.1159	0.7610	0.2481	0.1248	0.4030	0.0578	0.5775	1	0.2295	0.4485	0.3697	0.6825	0.6985	1	0.1012	0.1241	0.0537	0.0339
Ovary	1	0	0.2260	0.5832	0.9885	0.3102	0.1873	0.8070	0.2809	0.8590	1	0.9695	0.9700	0.9507	0.4840	0.3525	1	0.2392	0.1874	0.2975	0.2086
Placenta amnion	1	0	0.2870	0.7006	0.9905	0.5479	0.4370	0.9075	0.2949	0.9830	1	0.9625	0.9225	0.8634	0.8270	0.4085	1	0.3097	0.4347	0.3616	0.5515
Fetal	6	0	0.2465	0.2404	0.8705	0.2135	0.0939	0.4280	0.6205	0.3055	1	0.4245	0.4385	0.7467	0.8055	0.8150	1	0.2044	0.0933	0.7031	0.7790
Digestive	12	0	0.2995	0.2300	0.7940	0.1963	0.1185	0.4200	0.3021	0.3305	1	0.2110	0.6655	0.4254	0.2840	0.7905	1	0.3864	0.1181	0.2985	0.2157
Epithelial	8	0	0.2420	0.1166	0.5900	0.1773	0.0776	0.3000	0.2864	0.3345	1	0.1825	0.4490	0.3234	0.7100	0.9415	1	0.0779	0.0763	0.2523	0.1434
Thymus	2	0	0.1435	0.1545	0.9160	0.3697	0.1025	0.7115	0.1150	0.5970	1	0.7830	0.7795	0.6034	0.5755	0.3375	1	0.1179	0.0988	0.1153	0.0711
Adipose	3	0	0.1655	0.5314	0.8590	0.3096	0.1951	0.4330	0.0921	0.7105	1	0.2675	0.5960	0.2266	0.4615	0.7605	1	0.4405	0.1849	0.0875	0.0573
Blood and T cell	14	1	0.2285	0.2380	0.4195	0.2095	0.0870	0.5310	0.1200	0.1355	1	0.7635	0.3850	0.5196	0.6460	0.7805	1	0.2358	0.0856	0.1005	0.0490
HSC and B cell	9	0	0.3385	0.6077	0.8140	0.2012	0.2660	0.4185	0.2275	0.3060	1	0.6070	0.6005	0.3234	0.6625	0.7715	1	0.5203	0.2589	0.1986	0.1117
iPSC	5	0	0.2475	0.1744	0.6075	0.4109	0.6150	0.7295	0.8999	0.2460	1	0.4490	0.4560	0.5128	0.4245	0.6510	1	0.1187	0.6154	0.9266	0.9499
Cell line	15	0	0.1155	0.1087	0.7355	0.1873	0.7727	0.2300	0.8660	0.3440	1	0.4555	0.4560	0.7113	0.7650	0.9595	1	0.0629	0.7725	0.9058	0.9794
Encode 2012	16	2	0.1510	0.0357	0.5705	0.1460	0.0668	0.2725	0.8613	0.4380	1	0.4435	0.5055	0.3672	0.9085	0.9725	1	0.0280	0.0655	0.8512	0.7765
All epigenomes	127	0	0.2010	0.3585	0.3380	0.0673	0.9480	0.3605	0.9826	0.1415	1	0.2410	0.2395	0.5961	0.9425	0.9370	1	0.2869	0.9482	0.9867	0.9972
Angular gyrus	1	0	0.1265	0.3288	0.9760	0.5301	0.1508	0.8090	0.1736	0.9310	1	0.9140	0.9455	0.9300	0.8420	0.3805	1	0.1551	0.1467	0.1599	0.0540
Anterior caudate	1	1	0.2480	0.3887	0.9555	0.4783	0.1201	0.7700	0.1313	0.8985	1	0.9060	0.9240	0.9239	0.8600	0.3115	1	0.2330	0.1147	0.1197	0.0359
Cingulate gyrus	1	0	0.2300	0.4471	0.9665	0.4222	0.1507	0.7895	0.1576	0.9205	1	0.8660	0.8900	0.8738	0.7495	0.2200	1	0.2446	0.1444	0.1366	0.0537
Germinal Matrix	1	3	0.3175	0.4430	0.9500	0.4904	0.1425	0.8495	0.0216	0.5260	1	0.4180	0.6045	0.8179	0.5955	0.3435	1	0.2423	0.1352	0.0255	0.0182
Hippocampus middle	1	1	0.2370	0.3955	0.9565	0.4817	0.1251	0.7640	0.1246	0.8835	1	0.5195	0.5215	0.8505	0.7625	0.5015	1	0.2338	0.1190	0.1052	0.0401
Inferior temporal lobe	1	1	0.2415	0.4091	0.9560	0.5093	0.1468	0.7620	0.1651	0.9010	1	0.4655	0.9025	0.9026	0.8040	0.3040	1	0.2404	0.1398	0.1465	0.0485
Dorsolateral prefrontal cortex	1	0	0.2305	0.5197	0.9610	0.5263	0.1626	0.7960	0.1673	0.9335	1	0.8830	0.9130	0.9011	0.8220	0.3770	1	0.2446	0.1575	0.1606	0.0611
Substantia nigra	1	1	0.2350	0.4226	0.9710	0.5691	0.1331	0.8155	0.1402	0.9075	1	0.9245	0.9370	0.9092	0.8410	0.8400	1	0.2463	0.1298	0.1254	0.0484
Fetal brain female	1	0	0.2005	0.4480	0.9420	0.4675	0.3491	0.5210	0.6555	0.8265	1	0.7875	0.8395	0.8601	0.5850	0.3845	1	0.2439	0.3470	0.6109	0.2716
Fetal brain male	1	0	0.5650	0.5765	0.9870	0.7079	0.4877	0.9425	0.7232	0.5160	1	0.9260	0.8760	0.8362	0.8720	0.7205	1	0.4728	0.4864	0.7466	0.6260
Neurospheres, cortex derived	1	2	0.2875	0.3331	0.9715	0.3831	0.2684	0.7805	0.0498	0.9840	1	0.3360	0.5430	0.6005	0.5310	0.2725	1	0.2167	0.2686	0.0450	0.1855
Neurospheres, ganglionic eminence derived	1	0	0.2080	0.3333	0.9475	0.4299	0.3614	0.5625	0.0959	0.7650	1	0.3910	0.8845	0.4910	0.5885	0.3790	1	0.2455	0.3538	0.0709	0.2048
Neuronal progenitors, H1 derived	1	1	0.1920	0.3534	0.9840	0.6872	0.0978	0.9190	0.0956	0.4045	1	0.9150	0.9420	0.9248	0.3920	0.6080	1	0.2101	0.0967	0.0914	0.0301
Neuronal progenitors, H9 derived	1	2	0.1415	0.4860	0.8775	0.2081	0.0388	0.7830	0.1499	0.5895	1	0.8750	0.8770	0.5888	0.5475	0.6890	1	0.4101	0.0441	0.1080	0.0889
Cultured neurons, H9 derived	1	2	0.2865	0.6202	0.9090	0.2923	0.1466	0.7530	0.0265	0.5935	1	0.9100	0.8710	0.5964	0.5370	0.6300	1	0.4647	0.1920	0.0496	0.0770

	==	P<=0.05
	==	P<=0.04
	==	P<=0.03
	==	p<=0.01

a)

Affected individuals

#	Gene name	chromosome	CNV0 coordinates (#1)		CNV0 coordinates (#2)		Gene coordinates		# of hits and CNV0 location				Distance between CNV0 and Gene	
			start	end	start	end	start	end	exonic	intronic	left	right	left	right
1	FAM5C	1	189,959,475	189,966,393	191,473,007	191,651,990	190,066,797	190,446,759			1	1	1,026,248	100,404
2	RGS18	1	191,473,007	191,651,990			192,127,592	192,154,945				1		475,602
3	RGS21	1	191,473,007	191,651,990			192,286,122	192,336,414				1		634,132
4	SCN7A	2	167,346,017	167,393,108			167,260,083	167,343,481			1		2,536	
5	DUSP19	2	184,794,451	184,802,442			183,943,287	183,964,722			1		829,729	
6	NCKAP1	2	184,794,451	184,802,442			183,789,579	183,903,586			1		890,865	
7	NUP35	2	184,794,451	184,802,442			183,984,750	184,026,408			1		768,043	
8	ZNF804A	2	184,794,451	184,802,442			185,463,093	185,804,214				1		660,651
9	NYAP2	2	227,341,510	227,347,609			226,265,602	226,595,471			1		746,039	
10	IRS1	2	227,341,510	227,347,609			227,596,033	227,663,506				1		248,424
11	RHBDD1	2	227,341,510	227,347,609			227,700,671	227,863,923				1		353,062
12	CXXC11	2	242,915,454	243,034,687			242,811,886	242,815,482			1		99,972	
13	D2HGDH	2	242,915,454	243,034,687			242,674,030	242,708,231			1		207,223	
14	DTYMK	2	242,915,454	243,034,687			242,615,157	242,626,383			1		289,071	
15	GAL3ST2	2	242,915,454	243,034,687			242,716,240	242,743,702			1		171,752	
16	ING5	2	242,915,454	243,034,687			242,641,456	242,668,896			1		246,558	
17	NEU4	2	242,915,454	243,034,687			242,750,160	242,758,739			1		156,715	
18	PDCD1	2	242,915,454	243,034,687			242,792,033	242,801,058			1		114,396	
19	CNTN6	3	1,782,524	1,787,582			1,134,620	1,445,278			1		337,246	
20	CNTN4	3	1,782,524	1,787,582			2,140,550	3,099,645				1		352,968
21	CNTN3	3	75,394,265	75,544,090			74,311,722	74,570,343			1		823,922	
22	ZNF717	3	75,394,265	75,544,090			75,786,029	75,834,255				1		241,939
23	C3orf58	3	143,637,504	144,490,664			143,690,640	143,711,210	1					
24	SLC9A9	3	143,637,504	144,490,664			142,984,064	143,567,373			1		70,131	
25	PABPC4L	4	134,871,302	135,192,678			135,117,489	135,122,903	1					
26	TAS2R1	5	9,904,421	9,925,065			9,629,109	9,630,463			1		273,958	
27	CCT5	5	9,904,421	9,925,065			10,250,282	10,266,501				1		325,217
28	CMBL	5	9,904,421	9,925,065			10,277,707	10,308,168				1		352,642
29	FAM173B	5	9,904,421	9,925,065			10,225,620	10,250,021				1		300,555
30	MARCH6	5	9,904,421	9,925,065			10,353,751	10,440,500				1		428,686
31	FBXO5	6	154,121,271	154,131,344			153,291,658	153,304,740			1		816,531	
32	MTRF1L	6	154,121,271	154,131,344			153,308,400	153,323,925			1		797,346	
33	RGS17	6	154,121,271	154,131,344			153,332,032	153,452,389			1		668,882	
34	IPCEF1	6	154,121,271	154,131,344			154,475,618	154,831,753				1		344,274
35	OPRM1	6	154,121,271	154,131,344			154,331,631	154,568,001				1		200,287

Tables S8a-b: Genomic distance between all homozygous deletions and the neighboring genes.

a) **76 genes for affected individuals** presented in Tables 2 or S5a.

b) **22 genes for unaffected individuals** presented in Table S5a.

Affected individuals

#	Gene name	chromosome	CNV0 coordinates (#1)		CNV0 coordinates (#2)		Gene coordinates		# of hits and CNV0 location				Distance between CNV0 and Gene	
			start	end	start	end	start	end	exonic	intronic	left	right	left	right
36	AGR3	7	16,900,135	16,915,437			16,899,030	16,921,613	3					
37	AGR2	7	16,900,135	16,915,437			16,832,264	16,844,738			3		55,397	
38	BZW2	7	16,900,135	16,915,437			16,685,759	16,746,148			3		153,987	
39	TSPAN13	7	16,900,135	16,915,437			16,793,351	16,824,161			3		75,974	
40	CD36	7	80,157,064	80,298,888			79,998,891	80,308,593	1					
41	GNAT3	7	80,157,064	80,298,888			80,087,987	80,141,242			1		15,822	
42	SEMA3C	7	80,157,064	80,298,888			80,371,854	80,551,675				1		72,966
43	VIPR2	7	159,049,219	159,062,334			158,820,866	158,937,649			1		111,570	
44	WDR60	7	159,049,219	159,062,334			158,649,269	158,738,883			1		310,336	
45	MSR1	8	15,937,585	16,026,095			15,965,387	16,050,300	1					
46	PSD3	8	18,852,675	18,862,258			18,384,813	18,871,196		1				
47	UNC5D	8	34,800,058	34,843,012			35,092,975	35,652,181				1		249,963
48	AK302451	10	81,512,254	81,597,918			81,266,126	81,270,678			1		241,576	
49	EIF5AL1	10	81,512,254	81,597,918			81,272,357	81,276,192			1		236,062	
50	SFTPA1	10	81,512,254	81,597,918			81,370,695	81,375,199			1		137,055	
51	SFTPA2	10	81,512,254	81,597,918			81,315,608	81,320,163			1		192,091	
52	ZCCHC24	10	81,512,254	81,597,918			81,142,083	81,205,383			1		306,871	
53	PLAC9	10	81,512,254	81,597,918			81,892,258	81,904,784				1		294,340
54	SFTPD	10	81,512,254	81,597,918			81,697,496	81,708,861				1		99,578
55	TMEM254	10	81,512,254	81,597,918			81,838,402	81,852,307				1		240,484
56	TMEM116	12	112,432,874	112,438,515			112,369,087	112,451,023		1				
57	ALDH2	12	112,432,874	112,438,515			112,204,691	112,247,789			1		185,085	
58	MAPKAPK5	12	112,432,874	112,438,515			112,280,032	112,331,228			1		101,646	
59	ERP29	12	112,432,874	112,438,515			112,451,152	112,461,024				1		12,637
60	HECTD4	12	112,432,874	112,438,515			112,597,992	112,819,896				1		159,477
61	NAA25	12	112,432,874	112,438,515			112,464,493	112,546,635				1		25,978
62	TRAFD1	12	112,432,874	112,438,515			112,563,349	112,591,408				1		124,834
63	FOXG1	14	28,475,766	28,500,776			29,236,278	29,239,483				1		735,502
64	MDGA2	14	47,966,854	47,969,469			47,308,828	48,144,157		1				
65	BCAS1	20	52,643,162	52,663,360			52,560,079	52,687,304	2					
66	CYP24A1	20	52,643,162	52,663,360			52,769,988	52,790,516				2		106,628
67	PFDN4	20	52,643,162	52,663,360			52,824,502	52,836,492				2		161,142
68	BTG3	21	18,802,512	18,822,175			18,965,968	18,985,268				1		143,793
69	CXADR	21	18,802,512	18,822,175			18,885,224	18,965,897				1		63,049
70	MTMR3	22	30,336,496	30,366,792			30,279,158	30,426,857	1					
71	ASCC2	22	30,336,496	30,366,792			30,184,597	30,234,293			1		102,203	
72	CABP7	22	30,336,496	30,366,792			30,116,344	30,127,820			1		208,676	
73	NF2	22	30,336,496	30,366,792			29,999,545	30,094,589			1		241,907	
74	UQCR10	22	30,336,496	30,366,792			30,163,358	30,166,402			1		170,094	
75	ZMAT5	22	30,336,496	30,366,792			30,126,945	30,163,000			1		173,496	
76	HORMAD2	22	30,336,496	30,366,792			30,476,453	30,573,062				1		109,661

b)

Unaffected siblings

#	Gene name	chromosome	CNV0 coordinates (#1)		CNV0 coordinates (#2)		Gene coordinates		# of hits and CNV0 location				Distance between CNV0 and Gene	
			start	end	start	end	start	end	exonic	intronic	left	right	left	right
1	PLEKHM3	2	208,971,007	208,972,807			208,686,012	208,890,284			1		80,723	
2	C2orf80	2	208,971,007	208,972,807			209,030,071	209,054,773				1		57,264
3	CRYGA	2	208,971,007	208,972,807			209,025,464	209,028,297				1		52,657
4	CRYGB	2	208,971,007	208,972,807			209,007,297	209,010,877				1		34,490
5	CRYGC	2	208,971,007	208,972,807			208,992,861	208,994,554				1		20,054
6	CRYGD	2	208,971,007	208,972,807			208,986,331	209,028,297				1		13,524
7	IDH1	2	208,971,007	208,972,807			209,100,953	209,119,806				1		128,146
8	PIKFYVE	2	208,971,007	208,972,807			209,130,991	209,223,475				1		158,184
9	TAS2R1	5	9,905,081	9,921,281			9,629,109	9,630,463			1		274,618	
10	CCT5	5	9,905,081	9,921,281			10,250,282	10,266,501				1		329,001
11	CMBL	5	9,905,081	9,921,281			10,277,707	10,308,168				1		356,426
12	FAM173B	5	9,905,081	9,921,281			10,225,620	10,250,021				1		304,339
13	6-Mar	5	9,905,081	9,921,281			10,353,751	10,440,500				1		432,470
14	SLCO6A1	5	102,044,030	102,054,330			101,707,649	101,834,720			1		209,310	
15	PAM	5	102,044,030	102,054,330			102,201,527	102,366,808				1		147,197
16	CSMD1	8	4,717,499	4,806,199			2,792,875	4,852,328		1				
17	CAMK1D	10	13,056,103	13,061,103			12,391,583	12,871,733			1		184,370	
18	CCDC3	10	13,056,103	13,061,103			12,938,625	13,043,704			1		12,399	
19	MCM10	10	13,056,103	13,061,103			13,203,554	13,253,104				1		142,451
20	OPTN	10	13,056,103	13,061,103			13,142,082	13,180,276				1		80,979
21	PHYH	10	13,056,103	13,061,103			13,319,796	13,342,130				1		258,693
22	UCMA	10	13,056,103	13,061,103			13,263,767	13,276,328				1		202,664

a)

	CNV #	chr	Start	End	Size (Kb)	Gene	Deletion of the entire TAD boundary				Deletion of one or more TAD boundaries			
							N	TAD ids (hg19)	N	TAD ids (hg38)	N	TAD ids (hg19)	N	TAD ids (hg38)
Affected individuals	1	1	189,959,475	189,966,393	6.9		0	.	0	.	0	.	0	.
	2	1	191,473,007	191,651,990	179		0	.	0	.	1	(35)	0	.
	3	2	167,346,017	167,393,108	47.1		0	.	0	.	7	(16),(17),(18),(21),(22),(29),(32)	5	(23),(29),(31),(45),(48)
	4	2	184,794,451	184,802,442	8		0	.	0	.	0	.	0	.
	5	2	227,341,510	227,347,609	6.1		0	.	0	.	0	.	0	.
	6	2	242,915,454	243,034,687	119.2		0	.	0	.	0	.	0	.
	7	3	1,782,524	1,787,582	5.1		0	.	0	.	0	.	0	.
	8	3	75,394,265	75,544,090	149.8		0	.	1	(51)	6	(1),(3),(8),(24),(26),(31)	16	(2),(3),(4),(12),(13),(15),(16),(23),(30),(34),(35),(40),(41),(42),(47),(51)
	9	3	143,637,504	144,490,664	853.2	***C3orf58***	0	.	1	(49)	4	(30),(33),(34),(35)	2	(46),(49)
	10	4	134,871,302	135,192,678	321.4	***PABPC4L***	0	.	1	(49)	0	.	2	(29),(49)
	11	5	9,904,421	9,925,065	20.6		0	.	0	.	0	.	0	.
	12	6	154,121,271	154,131,344	10.1		0	.	0	.	0	.	0	.
	13	7	16,900,135	16,915,437	15.3	***AGR3***	0	.	0	.	0	.	0	.
	14	7	16,900,135	16,915,437	15.3	***AGR3***	0	.	0	.	0	.	0	.
	15	7	16,900,135	16,915,437	15.3	***AGR3***	0	.	0	.	0	.	0	.
	16	7	80,157,064	80,298,888	141.8	***CD36***	0	.	0	.	3	(3),(16),(18)	4	**(24)**,(26),(31),(32)
	17	7	159,049,219	159,062,334	13.1		0	.	0	.	0	.	0	.
	18	8	15,937,585	16,026,095	88.5	***MSR1***	0	.	0	.	1	(25)	0	.
	19	8	18,852,675	18,862,258	9.6		0	.	0	.	0	.	0	.
	20	8	34,800,058	34,843,012	43		0	.	0	.	1	(19)	1	(36)
	21	10	81,512,254	81,597,918	85.7		0	.	0	.	1	(6)	0	.
	22	12	112,432,874	112,438,515	5.6		0	.	0	.	0	.	1	(21)
	23	14	28,475,766	28,500,776	25		0	.	0	.	0	.	0	.
	24	14	47,966,854	47,969,469	2.6		0	.	0	.	0	.	0	.
	25	20	52,643,162	52,663,360	20.2	***BCAS1***	0	.	0	.	0	.	1	(5)
	26	20	52,643,162	52,663,360	20.2	***BCAS1***	0	.	0	.	0	.	1	(5)
	27	21	18,802,512	18,822,175	19.7		0	.	0	.	0	.	0	.
	28	22	30,336,496	30,366,792	30.3	***MTMR3***	0	.	0	.	0	.	0	.
												.		.
Unaffected siblings	1	2	208,971,007	208,972,807	1.8		0	.	0	.	0	.	0	.
	2	5	9,905,081	9,921,281	16.2		0	.	0	.	0	.	0	.
	3	5	102,044,030	102,054,330	10.3		0	.	0	.	0	.	0	.
	4	8	4,717,499	4,806,199	88.7		0	.	0	.	0	.	0	.
	5	10	13,056,103	13,061,103	5		0	.	0	.	0	.	0	.

Table S9: Homozygous deletions in TADs (Topologically Associated Domains).

a) Homozygous deletions overlapping TAD boundaries from the 3D Genome Browser database (Yue Lab, Northwestern University).

b) File information for TAD ids (TAD publications available at http://promoter.bx.psu.edu/hi-c/publications.html).

b)

TAD id	File information (hg19)
1	A549_raw-rep1_TADs.txt"
2	AdrenalGland_Donor-AD2-raw_TADs.txt"
3	Aorta_STL002_Leung2015-raw_TADs.txt"
4	Bladder_Donor-BL1-raw_TADs.txt"
5	Bowel_Small_Donor-SB2-raw_TADs.txt"
6	Caki2_raw-rep1_TADs.txt"
7	Cortex_DLPFC_Donor-CO-raw_TADs.txt"
8	G401_raw-rep1_TADs.txt"
9	GM12878_Lieberman-raw_TADs.txt"
10	H1-ESC_Dixon2015-raw_TADs.txt"
11	H1-MES_Dixon2015-raw_TADs.txt"
12	H1-MSC_Dixon2015-raw_TADs.txt"
13	H1-NPC_Dixon2015-raw_TADs.txt"
14	H1-TRO_Dixon2015-raw_TADs.txt"
15	HMEC_Lieberman-raw_TADs.txt"
16	HUVEC_Lieberman-raw_TADs.txt"
17	IMR90_Lieberman-raw_TADs.txt"
18	K562_Lieberman-raw_TADs.txt"
19	KBM7_Lieberman-raw_TADs.txt"
20	Liver_STL011_Leung2015-raw_TADs.txt"
21	LNCaP_raw-rep1_TADs.txt"
22	Lung_Donor-LG1-raw_TADs.txt"
23	Muscle_Psoas_Donor-PO1-raw_TADs.txt"
24	NCIH460_raw-rep1_TADs.txt"
25	NHEK_Lieberman-raw_TADs.txt"
26	PANC1_raw-rep1_TADs.txt"
27	Pancreas_Donor-PA2-raw_TADs.txt"
28	RPMI7951_raw-rep1_TADs.txt"
29	SJCRH30_raw-rep1_TADs.txt"
30	SKMEL5_raw-rep1_TADs.txt"
31	SKNDZ_raw-rep1_TADs.txt"
32	SKNMC_raw-rep1_TADs.txt"
33	Spleen_Donor-PX1-raw_TADs.txt"
34	T470_raw-rep1_TADs.txt"
35	Thymus_STL001_Leung2015-raw_TADs.txt"
36	VentricleLeft_STL003_Leung2015-raw_TADs.txt"
37	VentricleRight_Donor-RV3-raw_TADs.txt"

TAD id	File information (hg38)
1	A549_raw-merged_TADs.txt"
2	Adrenal_Schmitt2016-raw_TADs.txt"
3	Aorta_STL002.Leung_2015-raw.domains"
4	Aorta_STL002_Leung_2015-raw_TADs.txt"
5	Bladder_Schmitt2016-raw_TADs.txt"
6	Bowel_Small_Schmitt2016-raw_TADs.txt"
7	Caki2_raw-merged_TADs.txt"
8	Cortex_DLPFC_Schmitt2016-raw_TADs.txt"
9	Epidermal_Keratinocyte_day0.Rubin_2017-raw.domains"
10	Epidermal_Keratinocyte_day3.Rubin_2017-raw.domains"
11	Epidermal_Keratinocyte_day6.Rubin_2017-raw.domains"
12	G401_raw-merged_TADs.txt"
13	GM12878_Lieberman_2009-raw_TADs.txt"
14	GM12878_Rao_2014-raw_TADs.txt"
15	H1-ESC_Dixon_2015-raw_TADs.txt"
16	H1-ESC.Jin_2013-raw.domains"
17	H1-MES_Dixon_2015-raw_TADs.txt"
18	H1-MSC_Dixon_2015-raw_TADs.txt"
19	H1-NPC_Dixon_2015-raw_TADs.txt"
20	H1-TRO_Dixon_2015-raw_TADs.txt"
21	HCT-116_RAD21-mAC_auxin_6hr.Rao_2017-raw.domains"
22	HCT-116_RAD21-mAC_no_auxin.Rao_2017-raw.domains"
23	HepG2.ENCODE3-raw.domains"
24	Hippocampus_Schmitt2016-raw_TADs.txt"
25	HMEC_Rao_2014-raw_TADs.txt"
26	HUVEC_Rao_2014-raw_TADs.txt"
27	IMR90_Flavopiridol.Jin_2013-raw.domains"
28	IMR90.Jin_2013-raw.domains"
29	IMR90_Rao_2014-raw_TADs.txt"
30	IMR90_TNF-a.Jin_2013-raw.domains"
31	K562_Lieberman_2009-raw_TADs.txt"
32	K562_Rao_2014-raw_TADs.txt"
33	KBM7_Rao_2014-raw_TADs.txt"
34	Liver_STL011.Leung_2015-raw.domains"
35	Liver_STL011_Leung_2015-raw_TADs.txt"
36	LNCAP_raw-merged_TADs.txt"
37	Lung_Schmitt2016-raw_TADs.txt"
38	NCIH460_raw-merged_TADs.txt"
39	NHEK_Rao_2014-raw_TADs.txt"
40	Ovary_Schmitt2016-raw_TADs.txt"
41	PANC1_raw-merged_TADs.txt"
42	Pancreas_Schmitt2016-raw_TADs.txt"
43	Psoas_Schmitt2016-raw_TADs.txt"
44	RPMI7951_raw-merged_TADs.txt"
45	SJCRH30_raw-merged_TADs.txt"
46	SKMEL5_raw-merged_TADs.txt"
47	SKNDZ_raw-merged_TADs.txt"
48	SKNMC_raw-merged_TADs.txt"
49	Spleen_Schmitt2016-raw_TADs.txt"
50	T470_raw-merged_TADs.txt"
51	Thymus_STL001.Leung_2015-raw.domains"
52	VentricleLeft_STL003.Leung_2015-raw.domains"
53	VentricleLeft_STL003_Leung_2015-raw_TADs.txt"
54	Ventricle_Right_Schmitt2016-raw_TADs.txt"

	CNV #	chr	Start	End	Size (Kb)	KnownGene		RefGene		Ensembl Gene	
						Function	Gene name	Function	Gene name	Function	Gene name
Affected individuals	1	1	189,959,475	189,966,393	6.9	intergenic	NONE,FAM5C	intergenic	NONE,BRINP3	ncRNA_exonic	ENSG00000230987
	2	1	191,473,007	191,651,990	179.0	intergenic	LOC440704,RGS18	intergenic	LOC440704,RGS18	intergenic	ENSG00000233882,ENSG00000223344
	3	2	167,346,017	167,393,108	47.1	UTR5	SCN7A	intergenic	SCN7A,XIRP2	UTR5	ENSG00000136546
	4	2	184,794,451	184,802,442	8.0	intergenic	NUP35,ZNF804A	intergenic	NUP35,MIR548AE1	ncRNA_exonic	ENSG00000234172
	5	2	227,341,510	227,347,609	6.1	intergenic	LOC646736,MIR5702	intergenic	LOC646736,MIR5702	intergenic	ENSG00000235070,ENSG00000263363
	6	2	242,915,454	243,034,687	119.2	exonic	BC101234	ncRNA_exonic	LINC01237,LOC102723927,LOC728323	ncRNA_exonic	ENSG00000220804,ENSG00000226423,ENSG00000232002,ENSG00000233806,ENSG00000237940,ENSG00000261186
	7	3	1,782,524	1,787,582	5.1	intergenic	CNTN6,CNTN4	intergenic	CNTN6,CNTN4	intergenic	ENSG00000214074,ENSG00000214073
	8	3	75,394,265	75,544,090	149.8	exonic	FAM86DP	ncRNA_exonic	FAM86DP	ncRNA_exonic	ENSG00000179799,ENSG00000230655,ENSG00000238278,ENSG00000239959,ENSG00000240665,ENSG00000242953,ENSG00000243674,ENSG00000244026,ENSG00000244699,ENSG00000263782,ENSG00000266685,ENSG00000272690
	9	3	143,637,504	144,490,664	853.2	exonic	C3orf58	exonic	C3orf58	exonic	ENSG00000181744
	10	4	134,871,302	135,192,678	321.4	exonic	PABPC4L	exonic	PABPC4L	exonic	ENSG00000254535
	11	5	9,904,421	9,925,065	20.6	upstream	LOC285692	upstream	LOC285692	upstream	ENSG00000249781
	12	6	154,121,271	154,131,344	10.1	intergenic	RGS17,OPRM1	intergenic	MIR7641-2,OPRM1	intergenic	ENSG00000199246,ENSG00000217085
	13	7	16,900,135	16,915,437	15.3	exonic	AGR3	exonic	AGR3	exonic	ENSG00000173467
	14	7	16,900,135	16,915,437	15.3	exonic	AGR3	exonic	AGR3	exonic	ENSG00000173467
	15	7	16,900,135	16,915,437	15.3	exonic	AGR3	exonic	AGR3	exonic	ENSG00000173467
	16	7	80,157,064	80,298,888	141.8	exonic	CD36	exonic	CD36	exonic	ENSG00000135218
	17	7	159,049,219	159,062,334	13.1	intergenic	VIPR2,NONE	intergenic	VIPR2,NONE	intergenic	ENSG00000229435,NONE
	18	8	15,937,585	16,026,095	88.5	exonic	MSR1	exonic	MSR1	exonic	ENSG00000038945
	19	8	18,852,675	18,862,258	9.6	intronic	PSD3	intronic	PSD3	ncRNA_exonic	ENSG00000253335,ENSG00000270255
	20	8	34,800,058	34,843,012	43.0	intergenic	Metazoa_SRP,UNC5D	intergenic	LINC01288,UNC5D	ncRNA_exonic	ENSG00000265560
	21	10	81,512,254	81,597,918	85.7	ncRNA_exonic	AX747158,DQ586890,LOC642361	ncRNA_exonic	LOC642361,NUTM2B-AS1	ncRNA_exonic	ENSG00000225484,ENSG00000226381,ENSG00000272447
	22	12	112,432,874	112,438,515	5.6	intronic	TMEM116	intronic	TMEM116	intronic	ENSG00000198270
	23	14	28,475,766	28,500,776	25.0	intergenic	BC148262,DD413682	intergenic	LINC00645,FOXG1-AS1	intergenic	ENSG00000223164,ENSG00000197358
	24	14	47,966,854	47,969,469	2.6	intronic	MDGA2	intronic	MDGA2	intronic	ENSG00000139915,ENSG00000272781
	25	20	52,643,162	52,663,360	20.2	exonic	BCAS1	exonic	BCAS1	exonic	ENSG00000064787
	26	20	52,643,162	52,663,360	20.2	exonic	BCAS1	exonic	BCAS1	exonic	ENSG00000064787
	27	21	18,802,512	18,822,175	19.7	ncRNA_exonic	C21orf37	ncRNA_exonic	LINC01549	exonic	ENSG00000232560
	28	22	30,336,496	30,366,792	30.3	UTR5	MTMR3	UTR5	MTMR3	ncRNA_exonic	ENSG00000221736
Unaffected siblings	1	2	208,971,007	208,972,807	1.8	intergenic	PLEKHM3,LOC100507443	intergenic	PLEKHM3,LOC100507443	downstream	ENSG00000229150
	2	5	9,905,081	9,921,281	16.2	intergenic	LOC285692,FAM173B	intergenic	LOC285692,FAM173B	intergenic	ENSG00000249781,ENSG00000249807
	3	5	102,044,030	102,054,330	10.3	intergenic	AX747345,PAM	intergenic	LINC00491,PAM	intergenic	ENSG00000250682,ENSG00000145730
	4	8	4,717,499	4,806,199	88.7	intronic	CSMD1	intronic	CSMD1	intronic	ENSG00000183117
	5	10	13,056,103	13,061,103	5.0	ncRNA_intronic	AK311458	intronic	CCDC3	intronic	ENSG00000151468

Table S10: Gene annotation. We present both function and gene names for homozygous deletions in Tables 2 or S5a using 3 different gene annotation sets (KnownGene, RefGene and Ensembl).

chr1:799462-1003628	204,167
chr1:1211291-1478152	266,862
chr1:1510800-1663830	153,031
chr1:1888368-2027900	139,533
chr1:2553757-2713326	159,570
chr1:3822702-3999636	176,935
chr1:10031305-10155282	123,978
chr1:12910135-13775025	864,891
chr1:17045645-17246170	200,526
chr1:24148987-24382233	233,247
chr1:25549736-25753637	203,902
chr1:25768936-25892879	123,944
chr1:26940680-27041713	101,034
chr1:29870823-30061851	191,029
chr1:35161801-35303774	141,974
chr1:38271494-38395946	124,453
chr1:46119350-46223085	103,736
chr1:51477087-51580636	103,550
chr1:52059407-52281483	222,077
chr1:103757080-104066999	309,920
chr1:104128772-104309131	180,360
chr1:108745750-108854165	108,416
chr1:108889276-109013457	124,182
chr1:109527860-109651309	123,450
chr1:120523901-121172839	648,939
chr1:121291079-144045338	22,754,260
chr1:144052045-144549928	497,886
chr1:144551841-144865626	313,786
chr1:145243315-145366694	123,380
chr1:145727682-146475218	747,537
chr1:147382549-147716689	334,141
chr1:147825661-148949493	1,123,833
chr1:149254942-149660939	405,998
chr1:149711558-149876123	164,566
chr1:150812953-150927151	114,199
chr1:155424064-155605734	181,671
chr1:183412989-183515427	102,439
chr1:203411858-203582330	170,473
chr1:205921858-206595009	673,152
chr1:223706933-223838673	131,741
chr1:224061068-224186259	125,192
chr1:235130525-235273193	142,669
chr1:243147434-243280884	133,451
chr1:248605657-248795851	190,195
chr1:248866902-249068928	202,027
chr2:4992042-5181372	189,331
chr2:16205385-16358714	153,330
chr2:27876665-27995780	119,116
chr2:63355519-63473442	117,924
chr2:87108210-87951609	843,400
chr2:87988855-88293961	305,107
chr2:89152367-89285769	133,403
chr2:89330819-89547862	217,044
chr2:89621111-90045247	424,137
chr2:90275543-95358798	5,083,256
chr2:95420293-95537571	117,279
chr2:96014890-96195729	180,840
chr2:97764449-97865828	101,380
chr2:97881266-98013813	132,548
chr2:98020522-98269109	248,588
chr2:106999829-107104691	104,863
chr2:110086490-110257184	170,695
chr2:110290159-110425135	134,977
chr2:110459504-110834560	375,057
chr2:110980345-111426337	445,993
chr2:112003162-112213243	210,082
chr2:112500034-112607821	107,788
chr2:113089266-113209442	120,177
chr2:114162982-114422970	259,989
chr2:130783695-130967895	184,201
chr2:131143135-131480603	337,469
chr2:131955246-132081111	125,866
chr2:149653890-149805870	151981
chr2:197617105-197741264	124160
chr2:203343875-203468658	124784
chr2:203570486-203693558	123073
chr2:242516104-242918670	402567
chr2:243014629-243199373	184745
chr3:37302755-37407090	104336
chr3:38186416-38293903	107488
chr3:46772877-46895375	122499
chr3:47659378-47780716	121339
chr3:50932323-51037257	104935
chr3:52334592-52435859	101268
chr3:66000289-66293687	293399
chr3:67908343-68065200	156858
chr3:76225039-76399198	174160
chr3:90264055-93548478	3284424
chr3:93681488-93819127	137640
chr3:120350504-120464659	114156
chr3:136364510-136466598	102089
chr3:137911686-138025135	113450
chr3:162504666-162606746	102081
chr3:195121109-195262271	141163
chr3:195262520-195433542	171023
chr3:197328214-197441583	113370
chr3:197833757-198022430	188674
chr4:552861-666211	113351
chr4:2517446-2631428	113983
chr4:3762122-3975052	212931
chr4:8719588-9372302	652715
chr4:26622609-26758117	135509
chr4:34206499-34336069	129571
chr4:49063478-52698756	3635279
chr4:69189047-69384605	195559
chr4:69489671-69591248	101578
chr4:87418443-87523393	104951
chr4:92906930-93007984	101055
chr4:100792951-100907575	114625
chr4:113488133-113595868	107736
chr4:123088889-123238963	150075
chr4:128766348-128899032	132960
chr4:132610112-132764480	154369
chr4:190777760-190915649	137890
chr4:190930893-191154276	223384
chr5:12765291-12977506	212216
chr5:13032210-13205664	173455
chr5:21405841-21528803	122963
chr5:27088528-27191707	103180
chr5:34157057-34258539	101483
chr5:45888663-46099552	210890
chr5:46174863-49560858	3385996
chr5:72181403-72298132	116730
chr5:138551147-138666371	115225
chr5:138773089-138949361	176273
chr5:175468054-175571150	103097
chr5:176904875-177377647	472773
chr5:180326616-180452272	125657
chr5:180696888-180915260	218373
chr6:1-165390	165390
chr6:9144886-9257156	112271
chr6:27534527-27649762	115236
chr6:31933517-32044833	111317
chr6:58039238-58146053	106816
chr6:58154540-58290664	136125
chr6:58777523-61972224	3194702
chr6:62098281-62236966	138686
chr6:95673908-95841231	167324
chr6:115399990-115587010	187021
chr6:157467156-157915203	448048
chr6:167838992-168300292	461301
chr6:170905976-171115067	209092
chr7:199502-795226	595725
chr7:1428475-1530464	101990
chr7:6753693-6963688	209996
chr7:45794954-45908688	113,735
chr7:56427044-56544710	117,667
chr7:56829510-57120696	291,187
chr7:57919979-62080740	4,160,762
chr7:62190867-62380542	189,676
chr7:62706185-62836932	130,748
chr7:62906128-63156418	250,291
chr7:64557393-64728077	170,685
chr7:64917977-65243595	325,619
chr7:65264523-65373850	109,328
chr7:72083746-72207442	123,697
chr7:72268293-72755519	487,227
chr7:74133928-75058379	924,452
chr7:76565749-76720581	154,833
chr7:99815246-99938863	123,618
chr7:100017453-100198385	180,933
chr7:100527710-100632789	105,080
chr7:102091734-102358319	266,586
chr7:102358613-102464619	106,007
chr7:130151082-130326836	175,755
chr7:143299256-143572285	273,030
chr7:143866531-144074928	208,398
chr7:149906046-150008329	102,284
chr7:153728143-153886614	158,472
chr7:154261960-154373157	111,198
chr8:1-190567	190,567
chr8:7011074-8069130	1,058,057
chr8:12002716-12220248	217,533
chr8:12242031-12402225	160,195
chr8:35826544-35928166	101,623
chr8:37758537-37971941	213,405
chr8:42851138-42951983	100,846
chr8:43701111-46924210	3,223,100
chr8:46944912-47046131	101,220
chr8:48102785-48218379	115,595
chr8:48506627-48642877	136,251
chr8:54962394-55063436	101,043
chr8:64030672-64171121	140,450
chr8:85901856-86027231	125,376
chr8:86550475-86841228	290,754
chr8:89610884-89712999	102,116
chr8:99636228-99748514	112,287
chr8:100191782-100293742	101,961
chr8:145281307-145653005	371,699
chr9:38771830-43529276	4,757,447
chr9:43553499-44768658	1,215,160
chr9:44873199-66516365	21,643,167
chr9:66526472-71015732	4,489,261
chr9:91908782-92010795	102,014
chr9:92340882-92454785	113,904
chr9:92510618-92679570	168,953
chr9:133059820-133229189	169,370
chr9:136964449-137102068	137,620
chr9:139134980-139270875	135,896
chr9:139795503-139897163	101,661
chr9:140003920-140376667	372,748
chr9:141087365-141213431	126,067
chr10:1-111954	111,954
chr10:17777535-18230258	452,724
chr10:38677022-38921351	244,330
chr10:39024545-42636466	3,611,922
chr10:42682132-42835340	153,209
chr10:46216414-46943376	726,963
chr10:47136995-47560112	423,118
chr10:47742075-48318618	576,544
chr10:48654941-49363008	708,068
chr10:51110278-51492301	382,024
chr10:51586513-51785325	198,813
chr10:74334757-74442816	108,060
chr10:81251343-81572797	321,455
chr10:88956886-89160383	203,498
chr10:124275142-124455523	180,382
chr10:128602100-128770867	168,768
chr10:135434550-135534747	100,198
chr11:1-206766	206,766
chr11:1111473-1249371	137,899
chr11:48783496-48891876	108,381
chr11:50635121-51192869	557,749
chr11:51263687-51375309	111,623
chr11:51563635-54835622	3,271,988
chr11:54858307-55030214	171,908
chr11:61033226-61134531	101,306
chr11:66451961-66562247	110,287
chr11:69699979-69814929	114,951
chr11:71329634-71492617	162,984
chr11:89466731-89832040	365,310
chr11:96261899-96467131	205,233
chr12:1-166332	166,332
chr12:9621532-9731636	110,105
chr12:11222367-11380186	157,820
chr12:34760330-37857750	3,097,421
chr12:37988058-38317759	329,702
chr12:56131051-56232175	101,125
chr12:57656704-57763519	106,816
chr12:109331621-109432615	100,995
chr12:113744591-113848533	103,943
chr12:121925103-122036524	111,422
chr12:122473662-122612145	138,484
chr12:123822502-124043295	220,794
chr13:1-19199311	19,199,311
chr13:52782583-52960708	178,126
chr13:53046835-53152134	105,300
chr13:57137095-57246426	109,332
chr13:86740598-86913429	172,832
chr13:112343348-112505754	162,407
chr13:114318238-114468002	149,765
chr13:114634183-114752801	118,619
chr14:1-20203124	20,203,124
chr14:45636506-45742686	106,181
chr14:58635067-58750901	115,835
chr14:58823032-58965714	142,683
chr14:61465859-61582315	116,457
chr14:64173849-64275758	101,910
chr14:106167660-106273249	105,590
chr14:106328826-106614664	285,839
chr14:106764924-106917290	152,367
chr15:1-20167088	20,167,088
chr15:20191740-20422586	230,847
chr15:20616670-20852149	235,480
chr15:21200250-21909390	709,141
chr15:21929650-22320056	390,407
chr15:22588018-22777874	189,857
chr15:23222283-23654293	432,011
chr15:28530358-28969664	439,307
chr15:29013163-29212946	199,784
chr15:30354302-30936284	581,983
chr15:32511554-32924799	413,246
chr15:34779924-34893103	113,180
chr15:43933894-44047637	113,744
chr15:55683139-55816984	133,846
chr15:62172984-62274939	101,956
chr15:72836389-72975501	139,113
chr15:75520377-75643758	123,382
chr15:82580181-83221391	641,211
chr15:84827965-85088393	260,429
chr15:102393236-102531392	138,157
chr16:2101748-2270112	168,365
chr16:2502640-2712027	209,388
chr16:14760733-15085753	325,021
chr16:15154897-15496157	341,261
chr16:16353165-16633360	280,196
chr16:16730603-16858307	127,705
chr16:18264087-18779835	515,749
chr16:21347618-21534303	186,686
chr16:21740273-21901306	161,034
chr16:22421320-22558796	137,477
chr16:28294820-28490516	195,697
chr16:28600380-28837905	237,526
chr16:28909621-29016527	106,907
chr16:29042013-29146426	104,414
chr16:29412502-29652487	239,986
chr16:30018499-30320306	301,808
chr16:32010672-34202672	2,192,001
chr16:34989373-35090199	100,827
chr16:35139484-46507943	11,368,460
chr16:67605793-67798004	192,212
chr16:90163274-90354753	191,480
chr17:285675-410450	124,776
chr17:18367377-18532743	165,367
chr17:18917236-19168911	251,676
chr17:21550541-21717444	166,904
chr17:22145505-25343370	3,197,866
chr17:26337891-26454406	116,516
chr17:26905926-27009748	103,823
chr17:29265620-29466721	201,102
chr17:34477385-34821057	343,673
chr17:36244357-36410558	166,202
chr17:38457150-38583605	126,456
chr17:39096617-39235234	138,618
chr17:43541833-43651039	109,207
chr17:44364055-44789284	425,230
chr17:56021876-56131754	109,879
chr17:58343818-58459242	115,425
chr17:58701191-58828401	127,211
chr17:60492145-60636825	144,681
chr17:62754671-62919170	164,500
chr17:66022659-66154675	132,017
chr17:77538516-77654710	116,195
chr17:77898933-78007612	108,680
chr17:79693135-79943474	250,340
chr17:81012184-81195210	183,027
chr18:1-160886	160,886
chr18:14205994-14346306	140,313
chr18:15106726-18595139	3,488,414
chr18:52055115-52220941	165,827
chr19:1-261032	261,032
chr19:750172-865405	115,234
chr19:1175395-1310135	134,741
chr19:4936949-5041177	104,229
chr19:7293118-7415722	122,605
chr19:8634765-8754906	120,142
chr19:14130214-14338262	208,049
chr19:17973157-18074996	101,840
chr19:18972007-19083102	111,096
chr19:24505636-27960005	3,454,370
chr19:36366010-36471835	105,826
chr19:36758896-36871294	112,399
chr19:42506043-42676480	170,438
chr19:42706309-42807808	101,500
chr19:49085732-49189414	103,683
chr19:50593992-50699503	105,512
chr20:26288534-29493192	3,204,659
chr20:29529996-29846302	316,307
chr20:35362635-35467712	105,078
chr20:61174647-61275063	100,417
chr20:62906513-63025520	119,008
chr21:1-10865932	10,865,932
chr21:11167247-14369206	3,201,960
chr21:14425541-14595263	169,723
chr21:15005150-15110824	105,675
chr22:1-16053757	16,053,757
chr22:16110035-16877134	767,100
chr22:18640299-18890966	250,668
chr22:20323210-20732808	409,599
chr22:21461606-21806400	344,795
chr22:21809184-21928640	119,457
chr22:22952238-23063177	110,940
chr22:46266330-46421841	155,512
chr22:50314808-50440650	125,843
chr22:50688347-50837223	148,877

Table S11: Low marker density regions in autosomes excluded for Monte-Carlo simulation (hg19, 371 genomic regions, 281.05Mb, Affymetrix 500K, Figure S6d, Methods: J).
